# A Historical Analysis of Randomized Controlled Trials in Rotator Cuff Tears

**DOI:** 10.3390/ijerph17186863

**Published:** 2020-09-20

**Authors:** Vincenzo Candela, Umile Giuseppe Longo, Calogero Di Naro, Gabriella Facchinetti, Anna Marchetti, Gaia Sciotti, Giulia Santamaria, Ilaria Piergentili, Maria Grazia De Marinis, Ara Nazarian, Vincenzo Denaro

**Affiliations:** 1 Department of Orthopedic and Trauma Surgery, Campus Bio-Medico University, Trigoria, 00128 Rome, Italy; v.candela@unicampus.it (V.C.); c.dinaro@unicampus.it (C.D.N.); ilaria.piergentili94@gmail.com (I.P.); denaro@unicampus.it (V.D.); 2 Department of Orthopedic and Trauma Surgery, Research Unit Nursing Science, Campus Bio-Medico University, Trigoria, 00128 Rome, Italy; g.facchinetti@unicampus.it (G.F.); a.marchetti@unicampus.it (A.M.); g.sciotti@unicampus.it (G.S.); g.santamaria@unicampus.it (G.S.); m.demarinis@unicampus.it (M.G.D.M.); 3 Carl J. Shapiro Department of Orthopaedic Surgery and Center for Advanced Orthopaedic Studies, Beth Israel Deaconess Medical Center, Harvard Medical School, Boston, MA 02215, USA; anazaria@bidmc.harvard.edu

**Keywords:** randomized controlled trial, rotator cuff tear, modified Coleman methodology score, consolidated standards of reporting trials, quality

## Abstract

*Background and objectives:* Our research aimed to evaluate the quality of reporting of randomized controlled trials (RCTs) linked to rotator cuff (RC) tears. The present study analyzed factors connected to the quality of the RCTs and trends in the quality of reporting through time. *Materials and Methods:* The online databases used to search all RCTs on the topic of RC surgery completed until March 2020 were PubMed and Ovid (MEDLINE). The quality of reporting was evaluated using the modified Coleman methodology score (MCMS) and the consolidated standards of reporting trials (CONSORT). *Results:* The online search found 957 articles. Finally, 183 studies were included in the quantitative synthesis. A total of 97 (53%) of 183 studies had a level of evidence I and 86 (47%) of 183 studies had a level of evidence II, according to the Oxford Center of Evidence Based Medicine (EBM). A statistically significant difference in MCMS between articles written before 2010 and articles written after 2010 was found. Articles written after 2010 had, on average, the highest Coleman score. The average number of CONSORT checklist items for each article across all analyzed RCTs was 21.67. The 37 studies completed up to 2010 averaged a number of checklist items of 19.97 and the studies completed between 2011 and 2019 averaged a number of checklist items of 22.10. A statistically significant difference in the number of checklist items between articles written before 2010 and articles written after 2010 was found. Articles written after 2010 had on average more checklist items. However, low correlation (0.26) between the number of checklist items for each article and the respective Coleman score was found. On the other hand, articles with the CONSORT diagram had a significantly high Coleman score. *Conclusions:* An improvement in the quantity and quality of RCTs relating to RC surgery over the analyzed period was found.

## 1. Introduction

Rotator cuff (RC) tears are among the most frequent injuries of the tendon-bone junction in the human population [1]. RC repair is one of the most common surgical operations for shoulder disorders [2] or may be the most common of all shoulder surgeries [3].

Randomized controlled trials (RCTs) can be useful to improve the health of patients affected by RC tears. Randomization provides one single important advantage over all the other methodologies: it equipoises known and unknown prognostic elements among treatment groups. For this reason, it is very important to draft an accurate report of the results to make the analysis scientifically creditable [4].

Evidence based medicine (EBM) aims to optimize clinical care with the use of published literature, evaluating not only medicine studies, but also other disciplines such as nursing, dentistry, public health, and health policy [5].

RCTs must be conducted with careful attention because there are a lot of known and unknown confounding factors. It is useful to implement factors such as the allocation of randomization and to perform an intent-to-treat analysis of the patients minimizing the bias effects [6].

However, the quality of RCT reports was improved when CONSORT (consolidated standards of reporting trials) was introduced to reduce the problems arising from the inadequate non-pharmacologic treatments (NPTs) such as surgery, rehabilitation, or physiotherapy RCT reports [7].

Our systematic analysis aims to widely evaluate the quality of reporting of RCTs linked to RC tears. In particular, our research investigated potential factors linked to the quality of the RCTs and reports quality trends over time.

## 2. Materials and Methods

### 2.1. Search Strategy and Study Eligibility

A systematic analysis of the literature was made following the preferred reported items for systematic review and meta-analysis statement (PRISMA). RCTs and prospective cohort studies on the topic of RC surgery published until 1 March 2020 were included. The following combinations of keywords were used: “rotator cuff”; “rotator cuff tear”; “rotator cuff repair”; “pain after rotator cuff repair”; “rotator cuff physiotherapy”; and “rotator cuff surgery”. All articles were initially screened for relevance by title and abstract excluding articles without an abstract and obtaining the full-text article if the abstract did not allow the investigators to assess the defined inclusion and exclusion criteria. A cross-reference search of the selected articles was also performed to obtain other relevant articles for the study. Inclusion criteria were: level of evidence I or II according to the Oxford Center of EBM; studies on human patients; English-language; and focus on RC tears. Exclusion criteria were: review articles; animal and cadaveric studies; commentary reports; pilot studies; meta-analysis; and conference papers. The articles (titles, abstracts and full-text) were screened independently by two researchers: two investigators (G.S and C.D.N) separately performed a careful reading of all papers and extracted data. In this review, the CONSORT checklist was used independently to assess each trial by the two reviewers, with particular attention to those items that were not addressed in the Coleman score. The CONSORT checklist is a series of 25 items and 37 checklist items focused on reporting how the trial was designed, analyzed, and interpreted. For each item, a yes/no answer, depending on the completeness of the information reported in the study is given. The flow diagram just displays the progress of all participants through the trial and is one of the items in the CONSORT checklist.

### 2.2. Data Abstraction

The data extracted were recorded using Word and Excel (Microsoft). Full name of the first author, year of publication, number of patients enrolled, number of patients lost at follow-up, level of evidence of the study, location of the study, age of patients, sex of patients, financial support, number of centers involved, the presence of CONSORT flow diagram, sample size, trial type and topic, mean follow-up, type of rehabilitation, and journal of publication were recorded.

### 2.3. Methodological Quality Assessment

The methodological quality of the included articles was independently evaluated by two reviewers using the modified Coleman methodology score (MCMS). The MCMS is characterized by the presence of eleven criteria evaluated through a scoring system between 0 and 100, where 100 indicates a high-quality study without chances, bias, and other confounding factors [8]. The MCMS was used for the statistical analysis to evaluate the correlation between the variables involved.

### 2.4. Assessment of Agreement

The data from the full-text selected articles were extracted independently by two authors (G.S., C.D.N.) and checked by a third author (G.F.). To ascertain the validity of the included studies, two reviewers independently evaluated the methodological quality and reliability of the findings through MCMS.

### 2.5. Statistical Analysis

To compare the MCMS of articles written before 2010 and articles written after 2010 and to find statistically significant differences in terms of MCMS, between studies containing a CONSORT diagram and studies without the flow-chart of the independent samples *t*-test was used. The inter-rater reliability of grading the Coleman score and the association between the number of checklist items and the Coleman score for each article with Pearson’s correlation were evaluated. The inter-rater reliability of grading the CONSORT checklist with percent agreement between raters was evaluated.

## 3. Results

### 3.1. Study Characteristics

**Figure 1:**
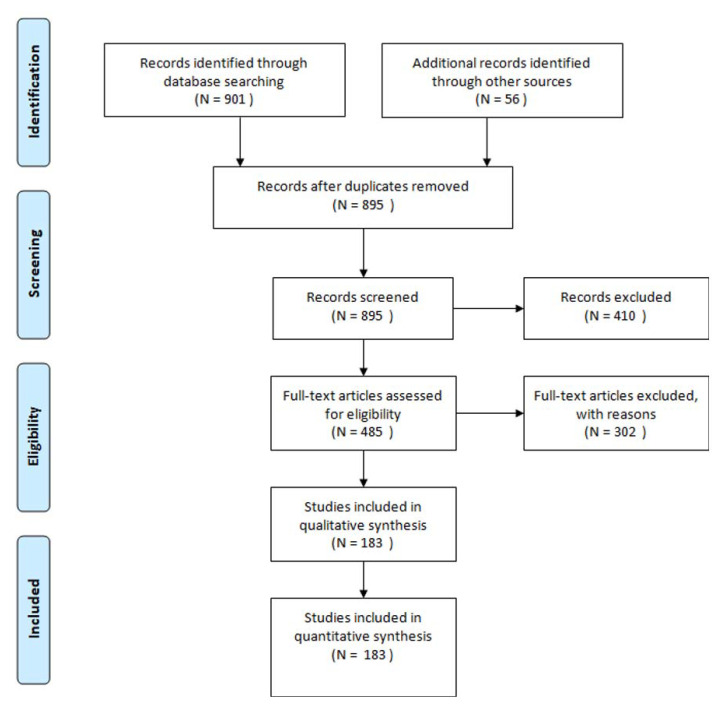
Preferred *R*eporting *I*tems for *S*ystematic Reviews and *M*eta-*A*nalyses (PRISMA) 2009 Flow diagram.

The selection process is illustrated in Figure 1. The literature search and cross-referencing resulted in a total of 957 articles. Finally, 183 studies were included in quantitative synthesis [1,2,3,4,5,6,7,8,9,10,11,12,13,14,15,16,17,18,19,20,21,22,23,24,25,26,27,28,29,30,31,32,33,34,35,36,37,38,39,40,41,42,43,44,45,46,47,48,49,50,51,52,53,54,55,56,57,58,59,60,61,62,63,64,65,66,67,68,69,70,71,72,73,74,75,76,77,78,79,80,81,82,83,84,85,86,87,88,89,90,91,92,93,94,95,96,97,98,99,100,101,102,103,104,105,106,107,108,109,110,111,112,113,114,115,116,117,118,119,120,121,122,123,124,125,126,127,128,129,130,131,132,133,134,135,136,137,138,139,140,141,142,143,144,145,146,147,148,149,150,151,152,153,154,155,156,157,158,159,160,161,162,163,164,165,166,167,168,169,170,171,172,173,174,175,176,177,178,179,180,181,182,183].

A total of 14,138 patients were initially analyzed in the involved studies. One thousand one hundred fifty patients were lost at the final follow-up [1,2,3,4,5,6,7,8,9,10,11,12,13,14,15,16,17,18,19,20,21,22,23,24,25,26,27,28,29,30,31,32,33,34,35,36,37,38,39,40,41,42,43,44,45,46,47,48,49,50,51,52,53,54,55,56,57,58,59,60,61,62,63,64,65,66,67,68,69,70,71,72,73,74,75,76,77,78,79,80,81,82,83,84,85,86,87,88,89,90,91,92,93,94,95,96,97,98,99,100,101,102,103,104,105,106,107,108,109,110,111,112,113,114,115,116,117,118,119,120,121,122,123,124,125,126,127,128,129,130,131,132,133,134,135,136,137,138,139,140,141,142,143,144,145,146,147,148,149,150,151,152,153,154,155,156,157,158,159,160,161,162,163,164,165,166,167,168,169,170,171,172,173,174,175,176,177,178,179,180,181,182,183]. A total of 97 (53%) of 183 studies had a level of evidence I and 86 (47%) of 183 studies had a level of evidence II, according to the Oxford Center of EBM. A total of 170 (93%) of the 183 studies were single-centered [3,9,10,11,12,13,14,15,16,17,18,19,20,21,22,23,24,25,26,27,28,29,30,32,33,34,37,38,39,40,41,42,43,44,45,46,47,48,49,50,51,52,53,54,55,56,57,59,60,61,62,63,64,65,66,67,68,69,70,71,72,73,74,75,76,77,78,79,80,81,82,83,84,85,86,87,88,89,90,91,92,93,94,95,96,97,98,99,100,101,102,103,104,105,106,107,108,109,110,111,112,113,114,115,116,117,118,119,120,121,122,123,124,125,126,127,128,129,130,131,132,133,134,135,136,137,138,139,140,141,142,143,144,146,147,148,149,150,151,152,153,154,155,156,157,158,159,160,162,163,164,165,166,167,168,169,170,171,172,173,174,175,176,177,178,179,180,181,182,183]; nine (5%) were multi-centered [4,5,7,31,35,36,58,145,161], and in four (2%) of the articles the data were not indicated [1,2,6,8].

Most of the included studies were published in the following three major journals: Arthroscopy (34 studies; 18.6%), The American Journal of Sports Medicine (30 studies; 16.4% of the total), and J Shoulder Elbow Surg (23 studies; 12.6%).

### 3.2. Topic

Among the 183 identified articles, six were focused on acromioplasty, 27 on the use of platelet rich plasma, platelet-rich fibrin matrix, leucocyte and platelet-rich fibrin and augmentation, 13 on single-row versus double-row repair, 54 on pain, five on conservative versus surgical repair, one on trauma-related rotator cuff tears, 31 on physiotherapy, six on the treatment of the long head of the biceps tendon disorder, four on mini-open versus arthroscopic repair, five on subacromial decompression, 10 on surgical techniques, four on post-operative immobilization, and 17 on other items linked to rotator cuff pathology (miscellaneous) (Supplementary Materials Tables S1–S13).

### 3.3. Modified Coleman Methodology Score (MCMS)

The inter-rater reliability of grading the Coleman score was 98.6%. The average MCMS across all analyzed RCTs was 71.40. The 37 studies completed up to 2010 averaged a MCMS of 68.54 and the studies completed between 2011 and 2019 averaged a MCMS of 72.12. (Table 1).

A statistically significant difference in MCMS between articles written before 2010 and articles written after 2010 was found (Table 2).

Articles with CONSORT diagram had the highest Coleman score (Table 3). Statistically significant differences in terms of MCMS between studies containing a CONSORT diagram or not were found (Table 4).

### 3.4. Trends

**Figure 2:**
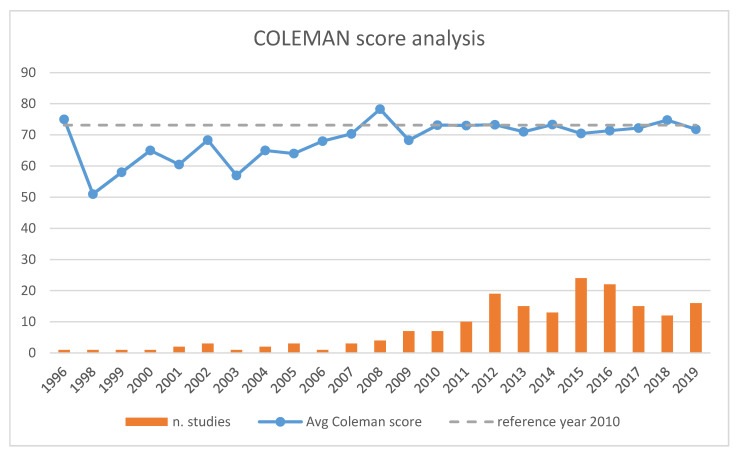
Coleman score analysis.

The majority of the identified studies had been produced since 2014. From about 2012, the number of RCTs published has increased significantly when compared to previous years (Figure 2).

### 3.5. Other Methodological Factors on the Consolidated Standards of Reporting Trials (CONSORT) Checklist

The inter-rater reliability of grading the CONSORT checklist was 97.4%.

The rate of missed checklist items of the CONSORT checklist for each trial, calculated as the ratio of number of checklist items with incomplete information to total checklist items for each trial, is shown in Supplementary Materials Table S14.

Items and checklist items most frequently lost were analyzed. Items “3b. Important changes to methods after trial commencement (such as eligibility criteria), with reasons” (182 missed, Trial Design item), “6b. Any changes to trial outcomes after the trial commenced, with reasons” (180 missed, Outcomes item), “7b. When applicable, explanation of any interim analyses and stopping guidelines” (181 missed, Sample Size item), “14b. Why the trial ended or was stopped” (183 missed, Recruitment item), “17b. For binary outcomes, presentation of both absolute and relative effect sizes is recommended” (178 missed, outcomes, and estimation item), and “18. Results of any other analyses performed including subgroup analyses and adjusted analyses, distinguishing pre-specified from exploratory” (181 missed, Ancillary analyses item) were the most frequently lost.

Low correlation (0.26) between the number of checklist items for each article and the respective Coleman score was found.

The average number of checklist items for each article across all analyzed RCTs was 21.67. The 37 studies completed until 2010 averaged a number of checklist items of 19.97 and the studies completed between 2011 and 2019 averaged a number of checklist items of 22.10 (Table 5).

A statistically significant difference in the number of checklist items between articles written before 2010 and articles written after 2010 was found (Table 6). Articles written after 2010 have on average more checklist items.

A CONSORT flow diagram that outlines the inclusion and exclusion of patients for the trial as well as follow-up rate was included in 47 studies (25.6% of total studies analyzed). Taking into consideration only the studies completed between 1996 and 2010, three included a CONSORT flow diagram. Next, the remaining 44 studies in which a flow diagram was found were completed between the years 2011 and 2019. All the analyzed studies completed until 2000 did not include a CONSORT flow diagram.

## 4. Discussion

Rotator cuff disease is among the most common musculoskeletal disorders [184]. It is a disabling condition with high prevalence rate [185], causing high direct and indirect costs [186]. The appropriate treatment for rotator cuff disease is debated. Rotator cuff repair is an option for patients with chronic, symptomatic full-thickness rotator cuff tear, but the quality of evidence is unconvincing. There is also little compelling evidence for conservative treatment [187]. RCTs are considered to be the most reliable form of scientific evidence in the hierarchy of evidence that influences healthcare policy and practice because RCTs reduce spurious causality and bias. Well done RCTs are the only way to find strong evidence on rotator cuff disease and treatment. This systematic review aimed to analyze factors connected to the quality of the RCTs and trends in the quality of reporting through time. The use of a CONSORT flow diagram, CONSORT checklist, and the MCMS have been found to be the most important predictive factor for a good quality RCT.

Rotator cuff repair can be performed with several techniques: transosseous simple suture, single-row suture anchor, double-row suture anchor, and transosseous-equivalent techniques. Current evidence has failed to prove a significant difference between single-row and double-row techniques in terms of clinical outcomes. The ideal rotator cuff repair technique should provide a good restoration of the footprint contact area and compression of the tendon on the footprint. Moreover, rotator cuff repair is not the only procedure undertaken to manage rotator cuff tears. Acromioplasty, long head of biceps tendon tenotomy, or tenodesis are surgical procedures often performed with rotator cuff repair. Acromioplasty is an arthroscopic surgical procedure made to prevent the impingement of the rotator cuff and consists of the decompression of subacromial space. Stem cell therapy is an emerging treatment for tendon disorders and can be used during arthroscopic rotator cuff repair, after rotator cuff repair, or instead of rotator cuff repair. The real efficacy of stem cell therapy for rotator cuff tears is nowadays debated. The 183 studies analyzed focused on this major topic concerning rotator cuffs. Pain resulted in being the most macro-area studied with 54 articles (29.5% of total RCTs), followed by physiotherapy (31 articles, 17% of total RCTs). Another popular topic is the use of stem cell therapy (27 articles, 15%).

The number of RCTs has increased over the last few years thanks to higher attention to the process of randomization and the prospective calculation of sample size. On the other hand, factors such as the description of concealment of patient allocation and the intention-to-treat analysis did not receive the same attention.

The MCMS, CONSORT flow diagram, and CONSORT checklist have been used to assess the quality of RCTs previously identified. This review showed a significant improvement in the quality in RCTs in the long term. This was confirmed by the studies completed until the year 2010 that had an average MCMS of 68.54 compared to the studies completed between 2011 and 2020 with an average of MCMS of 72.12. A statistically significant difference in MCMS between articles written before 2010 and articles written after 2010 was found. The main limitation of MCMS is its capacity to evaluate only the quality of reporting and not the quality of the study itself; hence high-quality studies that are reported poorly would receive a low score.

On the other hand, the CONSORT checklist is a series of 25 items focused on reporting how the trial was designed, analyzed, and interpreted. The CONSORT checklist was introduced to reduce the problems arising from the inadequate NPTs and to improve the quality of RCT reports [7]. The CONSORT statement is an evidence-based minimum set of recommendations including a checklist and flow diagram for reporting RCTs to improve the complete reporting of trials [57]. In 2008, the CONSORT Group developed an extension to the original statement that addressed methodological issues specific to trials of NPTs such as surgery, rehabilitation, or psychotherapy [59]. Thus, a modified checklist, aimed specifically at trials with non-pharmacologic interventions, was created, with a detailed explanation published in 2008 [73]. The factors outlined in this extension included descriptions of how such trials should outline the randomization procedure used, calculate sample sizes, report the blinding status, and outline the flow of participants, with flow diagrams strongly recommended [79].

The average number of CONSORT checklist items for each article across all analyzed RCTs was 21.67. The 37 studies completed until the year 2010 averaged a number of checklist items of 19.97 and the studies completed between 2011 and 2019 averaged a number of checklist items of 22.10. A statistically significant difference in the number of checklist items between articles written before 2010 and articles written after 2010 was found. Articles written after 2010 had on average more checklist items. However, low correlation (0.26) between the number of checklist items for each article and the respective Coleman score was found. On the other hand, articles with a CONSORT diagram had a significant high Coleman score.

Taking into consideration only the studies completed between 1996 and 2010, three included a CONSORT flow diagram. The remaining 44 studies in which we found a flow diagram were completed between the years 2011 and 2020. All of the analyzed studies completed up to 2000 did not include a CONSORT flow diagram.

There are several limitations related to the use of MCMS to measure the quality of RCTs. This index assesses the quality of reporting of the trials rather than the actual quality of the trials themselves. In fact, some trials may have used higher methodological safeguards that were not reported in the study. Moreover, certain factors that compose the MCMS can be only addressed to surgical trials (e.g., appropriate description of surgical technique and description of postoperative protocol). Therefore, by definition, non-surgical studies could never reach the maximum score of 100.

This study included only English-language trial and those indexed in PubMed and Ovid (MEDLINE); thus, our analysis did not address RCTs on RC surgery in other languages or those that were not indexed in these databases. Moreover, RCTs focused on shoulder pain with intact rotator cuff tendons were excluded [188]. High-certainty evidence shows that subacromial decompression does not provide clinically important benefits over placebo in pain, function, or health-related quality of life [189].

In this context, despite the “new” updated checklist specifics to evaluate a report of a non-pharmacological trial, it is often impossible to perform sham interventions or to blind patients and care providers.

Future research might investigate the impact that RCTs have had on clinical practices in RC surgery over time including the effect of specific methodological factors. It is also important to evaluate the quality of reporting in other areas of orthopedics to compare areas that require improvements.

## 5. Conclusions

The last decade was characterized by a significant increase in the quantity of RCTs evaluating RC repairs. The quality of RCT reporting (assessed through MCMS and other quality indexes) has shown a steady increase in results after 2010 compared to previous years. In particular, significant improvements in the reporting of the randomization process as well as prospective sample size calculation were noted. Statistically significant differences, in terms of MCMS, between studies containing a CONSORT diagram or not were found. Articles with a CONSORT diagram had the highest Coleman score. A low correlation between the number of checklist items for each article and the respective Coleman score was found.

## Figures and Tables

**Table 1:** Mean Coleman score in articles written before 2010 and articles written after 2010.

Years	Mean Coleman Score	Number of Studies	Standard Deviation
1996–2010	68.54	37	8927
2011–2019	72.12	146	7869
Total	71.40	183	8196

**Table 2:** Difference in modified Coleman methodology score (MCMS) between articles written before 2010 and articles written after 2010 (using Independent Samples *t*-test).

t	df	Significance (2-tailed)	Mean Difference	Std. Error Difference	95% Confidence Interval of the Difference Lower Upper	
−2231	51,079	0.030	−3583	1606	−6806	−0.359

**Table 3:** Difference in mean Coleman score between articles with and without a CONSORT diagram.

CONSORT Flow Diagram	Mean Coleman Score	Number of Studies	Standard Deviation
NO	70.46	136	8409
YES	74.11	47	6941
Total	71.40	183	8196

**Table 4:** Differences in terms of MCM, between studies containing a CONSORT diagram or not (using the Independent Samples *t*-test).

t	df	Significance (2-tailed)	Mean Difference	Std. Error Difference	95% Confidence Interval of the Difference Lower Upper	
2931	96,069	0.004	3643	1243	1176	6111

**Table 5:** Mean number of checklist items in articles written before 2010 and articles written after 2010.

Years	Mean Number of Checklist Items	Number of Studies	Standard Deviation
1996–2010	19.97	37	3594
2011–2019	22.10	146	2822
Total	21.67	183	3103

**Table 6:** Difference in the number of checklist items between articles written before 2010 and articles written after 2010 (using the Independent Samples *t*-test).

t	df	Significance (2-tailed)	Mean Difference	Std. Error Difference	95% Confidence Interval of the Difference Lower Upper	
−3342	47,839	0.002	−2123	0.635	−3400	−0.845

## Supplementary Materials

The following are available online at https://www.mdpi.com/1660-4601/17/18/6863/s1, Table S1: Acromioplasty; Table S2: PRP–PRFM-platelet-leukocyte membrane–mesenchymal stem cell–augmentation; Table S3: Single row–double row; Table S4: Pain; Table S5: Conservative vs surgical; Table S6: Trauma-related rotator cuff tears; Table S7: Physiotherapy; Table S8: Tenotomy and tenodesis; Table S9: Mini open and arthroscopic repair; Table S10: Subacromial decompression; Table S11: Surgical tecniques; Table S12: Miscellaneous; Table S13: Post-operative immobilization; Table S14: The rate of missed checklist items of consort checklist for each trial.

## References

[1] Abrams G.D., Gupta A.K., Hussey K.E., Tetteh E.S., Karas V., Bach B.R., Cole B.J., Romeo A.A., Verma N.N. (2014). Arthroscopic Repair of Full-Thickness Rotator Cuff Tears With and Without Acromioplasty: Randomized Prospective Trial With 2-Year Follow-up. Am. J. Sports Med..

[2] Dezaly C., Sirveaux F., Philippe R., Wein-Remy F., Sedaghatian J., Roche O., Molé D. (2011). Arthroscopic treatment of rotator cuff tear in the over-60s: Repair is preferable to isolated acromioplasty-tenotomy in the short term. Orthop. Traumatol. Surg. Res..

[3] Jacquot A., Dezaly C., Goetzmann T., Roche O., Sirveaux F., Molé D., French Society for Shoulder E.b.S. (2014). Is rotator cuff repair appropriate in patients older than 60 years of age? prospective, randomised trial in 103 patients with a mean four-year follow-up. Orthop. Traumatol. Surg. Res..

[4] MacDonald P., McRae S., Leiter J., Mascarenhas R., Lapner P. (2011). Arthroscopic rotator cuff repair with and without acromioplasty in the treatment of full-thickness rotator cuff tears: A multicenter, randomized controlled trial. J. Bone Join. Surg. Am. Vol..

[5] Mohtadi N.G., Hollinshead R.M., Sasyniuk T.M., Fletcher J.A., Chan D.S., Li F.X. (2008). A randomized clinical trial comparing open to arthroscopic acromioplasty with mini-open rotator cuff repair for full-thickness rotator cuff tears: Disease-specific quality of life outcome at an average 2-year follow-up. Am. J. Sports Med..

[6] Shin S.J., Oh J.H., Chung S.W., Song M.H. (2012). The efficacy of acromioplasty in the arthroscopic repair of small- to medium-sized rotator cuff tears without acromial spur: Prospective comparative study. Arthroscopy.

[7] Cai Y., Sun Z., Liao B., Zhanqiang S., Xiao T., Zhu P. (2018). Sodium Hyaluronate and Platelet-Rich Plasma for Partial-Thickness Rotator Cuff Tears. Med. Sci. Sports Exerc..

[8] Castricini R., Longo U.G., De Benedetto M., Panfoli N., Pirani P., Zini R., Maffulli N., Denaro V. (2011). Platelet-rich plasma augmentation for arthroscopic rotator cuff repair: A randomized controlled trial. Am. J. Sports Med..

[9] Aagaard K.E., Lunsjö K., Frobell R. (2019). Early repair of trauma-related full-thickness rotator cuff tears does not eliminate the problem of healing failure. Bone Joint J..

[10] Akbaba Y.A., Mutlu E.K., Altun S., Turkmen E., Birinci T., Celik D. (2019). The effectiveness of trigger point treatment in rotator cuff pathology: A randomized controlled double-blind study. J. Back Musculoskelet Rehabil..

[11] Alvarez C.M., Litchfield R., Jackowski D., Griffin S., Kirkley A. (2005). A prospective, double-blind, randomized clinical trial comparing subacromial injection of betamethasone and xylocaine to xylocaine alone in chronic rotator cuff tendinosis. Am. J. Sports Med..

[12] Analan P.D., Leblebici B., Adam M. (2015). Effects of therapeutic ultrasound and exercise on pain, function, and isokinetic shoulder rotator strength of patients with rotator cuff disease. J. Phys. Ther. Sci..

[13] Analay Akbaba Y., Kaya Mutlu E., Altun S., Celik D. (2018). Does the patients' expectations on kinesiotape affect the outcomes of patients with a rotator cuff tear? A randomized controlled clinical trial. Clin. Rehabil..

[14] Arias-Buría J.L., Valero-Alcaide R., Cleland J.A., Salom-Moreno J., Ortega-Santiago R., Atín-Arratibel M.A., Fernández-de-las-Peñas C. (2015). Inclusion of trigger point dry needling in a multimodal physical therapy program for postoperative shoulder pain: A randomized clinical trial. J. Manipulat. Physiol. Ther..

[15] Arndt J., Clavert P., Mielcarek P., Bouchaib J., Meyer N., Kempf J.F., the French Society for Shoulder & Elbow (SOFEC) (2012). Immediate passive motion versus immobilization after endoscopic supraspinatus tendon repair: A prospective randomized study. Orthop. Traumatol. Surg. Res..

[16] Avanzi P., Giudici L.D., Capone A., Cardoni G., Lunardi G., Foti G., Zorzi C. (2019). Prospective randomized controlled trial for patch augmentation in rotator cuff repair: 24-month outcomes. J. Shoulder Elb. Surg..

[17] Banerjee S.S., Pulido P., Adelson W.S., Fronek J., Hoenecke H.R. (2008). The efficacy of continuous bupivacaine infiltration following arthroscopic rotator cuff repair. Arthroscopy.

[18] Bang S.R., Yu S.K., Kim T.H. (2010). Can gabapentin help reduce postoperative pain in arthroscopic rotator cuff repair? A prospective, randomized, double-blind study. Arthroscopy.

[19] Barber F.A., Burns J.P., Deutsch A., Labbé M.R., Litchfield R.B. (2012). A prospective, randomized evaluation of acellular human dermal matrix augmentation for arthroscopic rotator cuff repair. Arthroscopy.

[20] Barber F.A., Herbert M.A. (2002). The effectiveness of an anesthetic continuous-infusion device on postoperative pain control. Arthroscopy.

[21] Baumgarten K.M., Osborn R., Schweinle W.E., Zens M.J., Helsper E.A. (2016). Are Pulley Exercises Initiated 6 Weeks After Rotator Cuff Repair a Safe and Effective Rehabilitative Treatment? A Randomized Controlled Trial. Am. J. Sports Med..

[22] Behr A., Freo U., Ori C., Westermann B., Alemanno F. (2012). Buprenorphine added to levobupivacaine enhances postoperative analgesia of middle interscalene brachial plexus block. J. Anesth..

[23] Belay E.S., Wittstein J.R., Garrigues G.E., Lassiter T.E., Scribani M., Goldner R.D., Bean C.A. (2019). Biceps tenotomy has earlier pain relief compared to biceps tenodesis: A randomized prospective study. Knee Surg. Sports Traumatol. Arth. Off. J. ESSKA.

[24] Berth A., Neumann W., Awiszus F., Pap G. (2010). Massive rotator cuff tears: Functional outcome after debridement or arthroscopic partial repair. J. Orthop. Traumatol..

[25] Bidwai A.S., Birch A., Temperley D., Odak S., Walton M.J., Haines J.F., Trail I. (2016). Medium- to long-term results of a randomized controlled trial to assess the efficacy of arthoscopic-subacromial decompression versus mini-open repair for the treatment of medium-sized rotator cuff tears. Shoulder Elb..

[26] Bigoni M., Gorla M., Guerrasio S., Brignoli A., Cossio A., Grillo P., Marinoni E.C. (2009). Shoulder evaluation with isokinetic strength testing after arthroscopic rotator cuff repairs. J. Shoulder Elb. Surg..

[27] Blanchard T.K., Bearcroft P.W., Constant C.R., Griffin D.R., Dixon A.K. (1999). Diagnostic and therapeutic impact of MRI and arthrography in the investigation of full-thickness rotator cuff tears. Eur. Radiol..

[28] Blum K., Chen A.L., Chen T.J., Waite R.L., Downs B.W., Braverman E.R., Kerner M.M., Savarimuthu S.M., DiNubile N. (2009). Repetitive H-wave device stimulation and program induces significant increases in the range of motion of post operative rotator cuff reconstruction in a double-blinded randomized placebo controlled human study. BMC Musculoskelet Disord..

[29] Boehm T.D., Werner A., Radtke S., Mueller T., Kirschner S., Gohlke F. (2005). The effect of suture materials and techniques on the outcome of repair of the rotator cuff: A prospective, randomised study. J. Bone Joint Surg. Br..

[30] Borgeat A., Aguirre J., Marquardt M., Mrdjen J., Blumenthal S. (2010). Continuous interscalene analgesia with ropivacaine 0.2% versus ropivacaine 0.3% after open rotator cuff repair: The effects on postoperative analgesia and motor function. Anesth. Analg..

[31] Burks R.T., Crim J., Brown N., Fink B., Greis P.E. (2009). A prospective randomized clinical trial comparing arthroscopic single- and double-row rotator cuff repair: Magnetic resonance imaging and early clinical evaluation. Am. J. Sports Med..

[32] Cai Y.Z., Zhang C., Jin R.L., Shen T., Gu P.C., Lin X.J., Chen J.D. (2018). Arthroscopic Rotator Cuff Repair With Graft Augmentation of 3-Dimensional Biological Collagen for Moderate to Large Tears: A Randomized Controlled Study. Am. J. Sports Med..

[33] Capito N.M., Cook J.L., Yahuaca B., Capito M.D., Sherman S.L., Smith M.J. (2017). Safety and efficacy of hyperosmolar irrigation solution in shoulder arthroscopy. J. Shoulder Elb. Surg..

[34] Carbonel I., Martinez A.A., Calvo A., Ripalda J., Herrera A. (2012). Single-row versus double-row arthroscopic repair in the treatment of rotator cuff tears: A prospective randomized clinical study. Int. Orthop..

[35] Carr A., Cooper C., Campbell M.K., Rees J., Moser J., Beard D.J., Fitzpatrick R., Gray A., Dawson J., Murphy J. (2017). Effectiveness of open and arthroscopic rotator cuff repair (UKUFF): A randomised controlled trial. Bone Joint J..

[36] Castagna A., Borroni M., Garofalo R., Rose G.D., Cesari E., Padua R., Conti M., Gumina S. (2015). Deep partial rotator cuff tear: Transtendon repair or tear completion and repair? A randomized clinical trial. Knee Surg. Sports Traumatol. Arth. Off. J. ESSKA.

[37] Chierichini A., Frassanito L., Vergari A., Santoprete S., Chiarotti F., Saccomanno M.F., Milano G. (2015). The effect of norepinephrine versus epinephrine in irrigation fluid on the incidence of hypotensive/bradycardic events during arthroscopic rotator cuff repair with interscalene block in the sitting position. Arthroscopy.

[38] Cho C.H., Lee S.W., Lee Y.K., Shin H.K., Hwang I. (2015). Effect of a sleep aid in analgesia after arthroscopic rotator cuff repair. Yonsei Med. J..

[39] Cho C.H., Song K.S., Jung G.H., Lee Y.K., Shin H.K. (2012). Early postoperative outcomes between arthroscopic and mini-open repair for rotator cuff tears. Orthopedics.

[40] Cho C.H., Song K.S., Min B.W., Lee K.J., Ha E., Lee Y.C., Lee Y.K. (2011). Multimodal approach to postoperative pain control in patients undergoing rotator cuff repair. Knee Surg. Sports Traumatol. Arth. Off. J. ESSKA.

[41] Cho N.S., Ha J.H., Rhee Y.G. (2007). Patient-controlled analgesia after arthroscopic rotator cuff repair: Subacromial catheter versus intravenous injection. Am. J. Sports Med..

[42] Choi S., Kim T., Kwon Y.S., Kang H. (2018). Intra-operative effect of interscalene brachial plexus block to arthroscopic rotator cuff repair surgery. Int. Orthop..

[43] Chou W.Y., Ko J.Y., Wang F.S., Huang C.C., Wong T., Wang C.J., Chang H.E. (2010). Effect of sodium hyaluronate treatment on rotator cuff lesions without complete tears: A randomized, double-blind, placebo-controlled study. J. Shoulder Elb. Surg..

[44] Coghlan J.A., Forbes A., McKenzie D., Bell S.N., Buchbinder R. (2009). Efficacy of subacromial ropivacaine infusion for rotator cuff surgery. A randomized trial. J. Bone Joint Surg. Am. Vol..

[45] Conti M., Garofalo R., Castagna A. (2015). Does a brace influence clinical outcomes after arthroscopic rotator cuff repair?. Musculoskelet Surg..

[46] Coory J.A., Parr A.F., Wilkinson M.P., Gupta A. (2019). Efficacy of suprascapular nerve block compared with subacromial injection: A randomized controlled trial in patients with rotator cuff tears. J. Shoulder Elb. Surg..

[47] Cuff D.J., Pupello D.R. (2012). Prospective randomized study of arthroscopic rotator cuff repair using an early versus delayed postoperative physical therapy protocol. J. Shoulder Elb. Surg..

[48] Culebras X., Van Gessel E., Hoffmeyer P., Gamulin Z. (2001). Clonidine combined with a long acting local anesthetic does not prolong postoperative analgesia after brachial plexus block but does induce hemodynamic changes. Anesth. Analg..

[49] D'Ambrosi R., Palumbo F., Paronzini A., Ragone V., Facchini R.M. (2016). Platelet-rich plasma supplementation in arthroscopic repair of full-thickness rotator cuff tears: A randomized clinical trial. Musculoskelet Surg..

[50] De Carli A., Vadalà A., Zanzotto E., Zampar G., Vetrano M., Iorio R., Ferretti A. (2012). Reparable rotator cuff tears with concomitant long-head biceps lesions: Tenotomy or tenotomy/tenodesis?. Knee Surg. Sports Traumatol. Arth. Off. J. ESSKA.

[51] De Roo P.J., Muermans S., Maroy M., Linden P., Van den Daelen L. (2015). Passive mobilization after arthroscopic rotator cuff repair is not detrimental in the early postoperative period. Acta Orthop. Belg..

[52] Delaunay L., Souron V., Lafosse L., Marret E., Toussaint B. (2005). Analgesia after arthroscopic rotator cuff repair: Subacromial versus interscalene continuous infusion of ropivacaine. Reg. Anesth. Pain Med..

[53] Desmet M., Vanneste B., Reynvoet M., Van Cauwelaert J., Verhelst L., Pottel H., Missant C., Van de Velde M. (2015). A randomised controlled trial of intravenous dexamethasone combined with interscalene brachial plexus blockade for shoulder surgery. Anaesthesia.

[54] Desroches A., Klouche S., Schlur C., Bauer T., Waitzenegger T., Hardy P. (2016). Suprascapular Nerve Block Versus Interscalene Block as Analgesia After Arthroscopic Rotator Cuff Repair: A Randomized Controlled Noninferiority Trial. Arthroscopy.

[55] Düzgün I., Baltacı G., Atay O.A. (2011). Comparison of slow and accelerated rehabilitation protocol after arthroscopic rotator cuff repair: Pain and functional activity. Acta Orthop. Traumatol. Turc..

[56] Düzgün İ., Baltacı G., Turgut E., Atay O.A. (2014). Effects of slow and accelerated rehabilitation protocols on range of motion after arthroscopic rotator cuff repair. Acta Orthop. Traumatol. Turc..

[57] Ebert J.R., Wang A., Smith A., Nairn R., Breidahl W., Zheng M.H., Ackland T. (2017). A Midterm Evaluation of Postoperative Platelet-Rich Plasma Injections on Arthroscopic Supraspinatus Repair: A Randomized Controlled Trial. Am. J. Sports Med..

[58] Flurin P.H., Hardy P., Abadie P., Desmoineaux P., Essig J., Joudet T., Sommaire C., Thelu C.E., French Arthroscopy Society (SFA) (2013). Rotator cuff tears after 70 years of age: A prospective, randomized, comparative study between decompression and arthroscopic repair in 154 patients. Orthop. Traumatol. Surg. Res..

[59] Flury M., Rickenbacher D., Schwyzer H.K., Jung C., Schneider M.M., Stahnke K., Goldhahn J., Audigé L. (2016). Does Pure Platelet-Rich Plasma Affect Postoperative Clinical Outcomes After Arthroscopic Rotator Cuff Repair? A Randomized Controlled Trial. Am. J. Sports Med..

[60] Franceschi F., Longo U.G., Ruzzini L., Rizzello G., Maffulli N., Denaro V. (2008). No advantages in repairing a type II superior labrum anterior and posterior (SLAP) lesion when associated with rotator cuff repair in patients over age 50: A randomized controlled trial. Am. J. Sports Med..

[61] Franceschi F., Papalia R., Del Buono A., Vasta S., Costa V., Maffulli N., Denaro V. (2013). Articular-sided rotator cuff tears: Which is the best repair? A three-year prospective randomised controlled trial. Int. Orthop..

[62] Franceschi F., Papalia R., Franceschetti E., Palumbo A., Del Buono A., Paciotti M., Maffulli N., Denaro V. (2016). Double-Row Repair Lowers the Retear Risk After Accelerated Rehabilitation. Am. J. Sports Med..

[63] Franceschi F., Ruzzini L., Longo U.G., Martina F.M., Zobel B.B., Maffulli N., Denaro V. (2007). Equivalent clinical results of arthroscopic single-row and double-row suture anchor repair for rotator cuff tears: A randomized controlled trial. Am. J. Sports Med..

[64] Ganokroj P., Matrakool L., Limsuwarn P., Sissaynarane T., Yimvassana C., Laoratanavoraphong S., Ratanawarinchai J. (2019). A Prospective Randomized Study Comparing the Effectiveness of Midlateral and Posterior Subacromial Steroid Injections. Orthopedics.

[65] Garofalo R., Conti M., Notarnicola A., Maradei L., Giardella A., Castagna A. (2010). Effects of one-month continuous passive motion after arthroscopic rotator cuff repair: Results at 1-year follow-up of a prospective randomized study. Musculoskelet Surg..

[66] Gartsman G.M., Drake G., Edwards T.B., Elkousy H.A., Hammerman S.M., O'Connor D.P., Press C.M. (2013). Ultrasound evaluation of arthroscopic full-thickness supraspinatus rotator cuff repair: Single-row versus double-row suture bridge (transosseous equivalent) fixation. Results of a prospective, randomized study. J. Shoulder Elb. Surg..

[67] Gartsman G.M., O'connor D.P. (2004). Arthroscopic rotator cuff repair with and without arthroscopic subacromial decompression: A prospective, randomized study of one-year outcomes. J. Shoulder Elb. Surg..

[68] Gervasi E., Maman E., Dekel A., Cautero E. (2016). Fluoroscopy-guided biodegradable spacer implantation using local anesthesia: Safety and efficacy study in patients with massive rotator cuff tears. Musculoskelet Surg..

[69] Ghandour T.M., Ibrahim A., Abdelrahman A.A., Elgammal A., Hammad M.H. (2019). Does The Type of Shoulder Brace Affect Postoperative Pain and Clinical Outcome After Arthroscopic Rotator Cuff Repair?. Arthroscopy.

[70] Gialanella B., Prometti P. (2011). Effects of corticosteroids injection in rotator cuff tears. Pain Med..

[71] Grasso A., Milano G., Salvatore M., Falcone G., Deriu L., Fabbriciani C. (2009). Single-row versus double-row arthroscopic rotator cuff repair: A prospective randomized clinical study. Arthroscopy.

[72] Greiner S., Ide J., Van Noort A., Mochizuki Y., Ochi H., Marraffino S., Sridharan S., Rudicel S., Itoi E. (2015). Local rhBMP-12 on an Absorbable Collagen Sponge as an Adjuvant Therapy for Rotator Cuff Repair–A Phase 1, Randomized, Standard of Care Control, Multicenter Study: Safety and Feasibility. Am. J. Sports Med..

[73] Gumina S., Campagna V., Ferrazza G., Giannicola G., Fratalocchi F., Milani A., Postacchini F. (2012). Use of platelet-leukocyte membrane in arthroscopic repair of large rotator cuff tears: A prospective randomized study. J. Bone Joint Surg. Am. Vol..

[74] Gumina S., Passaretti D., Gurzì M.D., Candela V. (2012). Arginine L-alpha-ketoglutarate, methylsulfonylmethane, hydrolyzed type I collagen and bromelain in rotator cuff tear repair: A prospective randomized study. Curr. Med. Res. Opin..

[75] Han S.S., Lee Y.H., Oh J.H., Aminzai S., Kim S.H. (2013). Randomized, controlled trial of multimodal shoulder injection or intravenous patient-controlled analgesia after arthroscopic rotator cuff repair. Knee Surg. Sports Traumatol. Arth. Off. J. ESSKA.

[76] Hartrick C.T., Tang Y.S., Siwek D., Murray R., Hunstad D., Smith G. (2012). The effect of initial local anesthetic dose with continuous interscalene analgesia on postoperative pain and diaphragmatic function in patients undergoing arthroscopic shoulder surgery: A double-blind, randomized controlled trial. BMC Anesthesiol..

[77] Hayes K., Ginn K.A., Walton J.R., Szomor Z.L., Murrell G.A. (2004). A randomised clinical trial evaluating the efficacy of physiotherapy after rotator cuff repair. Aust. J. Physiother..

[78] Hollman F., Wolterbeek N., Zijl J.A.C., van Egeraat S.P.M., Wessel R.N. (2017). Abduction Brace Versus Antirotation Sling After Arthroscopic Cuff Repair: The Effects on Pain and Function. Arthroscopy.

[79] Holtby R., Christakis M., Maman E., MacDermid J.C., Dwyer T., Athwal G.S., Faber K., Theodoropoulos J., Woodhouse L.J., Razmjou H. (2016). Impact of Platelet-Rich Plasma on Arthroscopic Repair of Small- to Medium-Sized Rotator Cuff Tears: A Randomized Controlled Trial. Orthop. J. Sports Med..

[80] Hufeland M., Wicke S., Verde P.E., Krauspe R., Patzer T. (2019). Biceps tenodesis versus tenotomy in isolated LHB lesions: A prospective randomized clinical trial. Arch. Orthop. Trauma Surg..

[81] Iannotti J.P., Codsi M.J., Kwon Y.W., Derwin K., Ciccone J., Brems J.J. (2006). Porcine small intestine submucosa augmentation of surgical repair of chronic two-tendon rotator cuff tears. A randomized, controlled trial. J. Bone Joint Surg. Am. Vol..

[82] Ide J., Mochizuki Y., van Noort A., Ochi H., Sridharan S., Itoi E., Greiner S. (2017). Local rhBMP-12 on an Absorbable Collagen Sponge as an Adjuvant Therapy for Rotator Cuff Repair–A Phase 1, Randomized, Standard of Care Control, Multicenter Study: Part 2-A Pilot Study of Functional Recovery and Structural Outcomes. Orthop. J. Sports Med..

[83] Ikemoto R.Y., Murachovsky J., Prata Nascimento L.G., Bueno R.S., Oliveira Almeida L.H., Strose E., de Mello S.C., Saletti D. (2010). Prospective Randomized Study Comparing Two Anesthetic Methods for Shoulder Surgery. Rev. Bras. Ortop..

[84] Ilhanli I., Guder N., Gul M. (2015). Platelet-Rich Plasma Treatment With Physical Therapy in Chronic Partial Supraspinatus Tears. Iran. Red. Crescent Med. J..

[85] Jenssen K.K., Lundgreen K., Madsen J.E., Kvakestad R., Pripp A.H., Dimmen S. (2018). No Functional Difference Between Three and Six Weeks of Immobilization After Arthroscopic Rotator Cuff Repair: A Prospective Randomized Controlled Non-Inferiority Trial. Arthroscopy.

[86] Jo C.H., Shin J.S., Huh J. (2014). Multimodal analgesia for arthroscopic rotator cuff repair: A randomized, placebo-controlled, double-blind trial. Eur. J. Orthop. Surg. Traumatol..

[87] Jo C.H., Shin J.S., Lee Y.G., Shin W.H., Kim H., Lee S.Y., Yoon K.S., Shin S. (2013). Platelet-rich plasma for arthroscopic repair of large to massive rotator cuff tears: A randomized, single-blind, parallel-group trial. Am. J. Sports Med..

[88] Jo C.H., Shin J.S., Shin W.H., Lee S.Y., Yoon K.S., Shin S. (2015). Platelet-rich plasma for arthroscopic repair of medium to large rotator cuff tears: A randomized controlled trial. Am. J. Sports Med..

[89] Keener J.D., Galatz L.M., Stobbs-Cucchi G., Patton R., Yamaguchi K. (2014). Rehabilitation following arthroscopic rotator cuff repair: A prospective randomized trial of immobilization compared with early motion. J. Bone Joint Surg. Am. Vol..

[90] Khashan M., Dolkart O., Amar E., Chechik O., Sharfman Z., Mozes G., Maman E., Weinbroum A. (2016). Effect of preemptive intra-articular morphine and ketamine on pain after arthroscopic rotator cuff repair: A prospective, double-blind, randomized controlled study. Arch. Orthop. Trauma Surg..

[91] Kim J., Chung J., Ok H. (2011). Asymptomatic acromioclavicular joint arthritis in arthroscopic rotator cuff tendon repair: A prospective randomized comparison study. Arch. Orthop. Trauma Surg..

[92] Kim J.H., Ha D.H., Kim S.M., Kim K.W., Han S.Y., Kim Y.S. (2019). Does arthroscopic preemptive extensive rotator interval release reduce postoperative stiffness after arthroscopic rotator cuff repair?: A prospective randomized clinical trial. J. Shoulder Elb. Surg..

[93] Kim J.H., Koh H.J., Kim D.K., Lee H.J., Kwon K.H., Lee K.Y., Kim Y.S. (2018). Interscalene brachial plexus bolus block versus patient-controlled interscalene indwelling catheter analgesia for the first 48 hours after arthroscopic rotator cuff repair. J. Shoulder Elb. Surg..

[94] Kim J.Y., Lee J.S., Park C.W. (2012). Extracorporeal shock wave therapy is not useful after arthroscopic rotator cuff repair. Knee Surg. Sports Traumatol. Arth. Off. J. ESSKA.

[95] Kim J.Y., Song K.S., Kim W.J., Park Y.H., Kang H., Woo Y.C., Shin H.Y. (2016). Analgesic efficacy of two interscalene blocks and one cervical epidural block in arthroscopic rotator cuff repair. Knee Surg. Sports Traumatol. Arth. Off. J. ESSKA.

[96] Kim J.Y., Song S.H., Cho J.H., Cho H.R. (2017). Comparison of clinical efficacy among remifentanil, nicardipine, and remifentanil plus nicardipine continuous infusion for hypotensive anesthesia during arthroscopic shoulder surgery. J. Orthop. Surg..

[97] Kim Y.S., Chung S.W., Kim J.Y., Ok J.H., Park I., Oh J.H. (2012). Is early passive motion exercise necessary after arthroscopic rotator cuff repair?. Am. J. Sports Med..

[98] Kim Y.S., Lee H.J., Jin H.K., Kim S.E., Lee J.W. (2016). Conventional En Masse Repair Versus Separate Double-Layer Double-Row Repair for the Treatment of Delaminated Rotator Cuff Tears. Am. J. Sports Med..

[99] Kim Y.S., Lee H.J., Kim J.H., Noh D.Y. (2018). When Should We Repair Partial-Thickness Rotator Cuff Tears? Outcome Comparison Between Immediate Surgical Repair Versus Delayed Repair After 6-Month Period of Nonsurgical Treatment. Am. J. Sports Med..

[100] Klintberg I.H., Gunnarsson A.C., Svantesson U., Styf J., Karlsson J. (2009). Early loading in physiotherapy treatment after full-thickness rotator cuff repair: A prospective randomized pilot-study with a two-year follow-up. Clin. Rehabil..

[101] Ko S.H., Cho S.D., Lee C.C., Choi J.K., Kim H.W., Park S.J., Bae M.H., Cha J.R. (2017). Comparison of Arthroscopically Guided Suprascapular Nerve Block and Blinded Axillary Nerve Block vs. Blinded Suprascapular Nerve Block in Arthroscopic Rotator Cuff Repair: A Randomized Controlled Trial. Clin. Orthop. Surg..

[102] Ko S.H., Lee C.C., Friedman D., Park K.B., Warner J.J. (2008). Arthroscopic single-row supraspinatus tendon repair with a modified mattress locking stitch: A prospective, randomized controlled comparison with a simple stitch. Arthroscopy.

[103] Koh K.H., Kang K.C., Lim T.K., Shon M.S., Yoo J.C. (2011). Prospective randomized clinical trial of single- versus double-row suture anchor repair in 2- to 4-cm rotator cuff tears: Clinical and magnetic resonance imaging results. Arthroscopy.

[104] Koh K.H., Lim T.K., Shon M.S., Park Y.E., Lee S.W., Yoo J.C. (2014). Effect of immobilization without passive exercise after rotator cuff repair: Randomized clinical trial comparing four and eight weeks of immobilization. J. Bone Joint Surg. Am. Vol..

[105] Koh W.U., Kim H.J., Park H.S., Choi W.J., Yang H.S., Ro Y.J. (2016). A randomised controlled trial comparing continuous supraclavicular and interscalene brachial plexus blockade for open rotator cuff surgery. Anaesthesia.

[106] Kraeutler M.J., Reynolds K.A., Long C., McCarty E.C. (2015). Compressive cryotherapy versus ice-a prospective, randomized study on postoperative pain in patients undergoing arthroscopic rotator cuff repair or subacromial decompression. J. Shoulder Elb. Surg..

[107] Kukkonen J., Joukainen A., Lehtinen J., Mattila K.T., Tuominen E.K., Kauko T., Aärimaa V. (2014). Treatment of non-traumatic rotator cuff tears: A randomised controlled trial with one-year clinical results. Bone Joint J..

[108] Lam P.H., Hansen K., Keighley G., Hackett L., Murrell G.A. (2015). A Randomized, Double-Blinded, Placebo-Controlled Clinical Trial Evaluating the Effectiveness of Daily Vibration After Arthroscopic Rotator Cuff Repair. Am. J. Sports Med..

[109] Lamas J.R., García-Fernández C., Tornero-Esteban P., Lópiz Y., Rodriguez-Rodriguez L., Ortega L., Fernández-Gutiérrez B., Marco F. (2019). Adverse effects of xenogenic scaffolding in the context of a randomized double-blind placebo-controlled study for repairing full-thickness rotator cuff tears. Trials.

[110] Lambers Heerspink F.O., van Raay J.J., Koorevaar R.C., van Eerden P.J., Westerbeek R.E., van’t Riet E., van den Akker-Scheek I., Diercks R.L. (2015). Comparing surgical repair with conservative treatment for degenerative rotator cuff tears: A randomized controlled trial. J. Shoulder Elb. Surg..

[111] Lapner P.L., Sabri E., Rakhra K., McRae S., Leiter J., Bell K., Macdonald P. (2012). A multicenter randomized controlled trial comparing single-row with double-row fixation in arthroscopic rotator cuff repair. J. Bone Joint Surg. Am. Vol..

[112] Lastayo P.C., Wright T., Jaffe R., Hartzel J. (1998). Continuous passive motion after repair of the rotator cuff. A prospective outcome study. J. Bone Joint Surg. Am. Vol..

[113] Lee B.G., Cho N.S., Rhee Y.G. (2012). Effect of two rehabilitation protocols on range of motion and healing rates after arthroscopic rotator cuff repair: Aggressive versus limited early passive exercises. Arthroscopy.

[114] Lee H.J., Jeong J.Y., Kim C.K., Kim Y.S. (2016). Surgical treatment of lesions of the long head of the biceps brachii tendon with rotator cuff tear: A prospective randomized clinical trial comparing the clinical results of tenotomy and tenodesis. J. Shoulder Elb. Surg..

[115] Lee H.J., Kim Y.S., Park I., Ha D.H., Lee J.H. (2015). Administration of analgesics after rotator cuff repair: A prospective clinical trial comparing glenohumeral, subacromial, and a combination of glenohumeral and subacromial injections. J. Shoulder Elb. Surg..

[116] Lee J.J., Hwang J.T., Kim D.Y., Lee S.S., Hwang S.M., Lee N.R., Kwak B.C. (2017). Effects of arthroscopy-guided suprascapular nerve block combined with ultrasound-guided interscalene brachial plexus block for arthroscopic rotator cuff repair: A randomized controlled trial. Knee Surg. Sports Traumatol. Arth. Off. J. ESSKA.

[117] Lee J.J., Kim D.Y., Hwang J.T., Lee S.S., Hwang S.M., Kim G.H., Jo Y.G. (2014). Effect of ultrasonographically guided axillary nerve block combined with suprascapular nerve block in arthroscopic rotator cuff repair: A randomized controlled trial. Arthroscopy.

[118] Lee J.J., Yoo Y.S., Hwang J.T., Kim D.Y., Jeon S.J., Hwang S.M., Jang J.S. (2015). Efficacy of direct arthroscopy-guided suprascapular nerve block after arthroscopic rotator cuff repair: A prospective randomized study. Knee Surg. Sports Traumatol. Arth. Off. J. ESSKA.

[119] Liu J., Fan L., Zhu Y., Yu H., Xu T., Li G. (2017). Comparison of clinical outcomes in all-arthroscopic versus mini-open repair of rotator cuff tears: A randomized clinical trial. Medicine.

[120] Liu X.N., Noh Y.M., Yang C.J., Kim J.U., Chung M.H., Noh K.C. (2017). Effects of a Single-Dose Interscalene Block on Pain and Stress Biomarkers in Patients Undergoing Arthroscopic Rotator Cuff Repair: A Randomized Controlled Trial. Arthroscopy.

[121] Ma H.L., Chiang E.R., Wu H.T., Hung S.C., Wang S.T., Liu C.L., Chen T.H. (2012). Clinical outcome and imaging of arthroscopic single-row and double-row rotator cuff repair: A prospective randomized trial. Arthroscopy.

[122] Mahure S.A., Rokito A.S., Kwon Y.W. (2017). Transcutaneous electrical nerve stimulation for postoperative pain relief after arthroscopic rotator cuff repair: A prospective double-blinded randomized trial. J. Shoulder Elb. Surg..

[123] Malavolta E.A., Gracitelli M.E., Ferreira Neto A.A., Assunção J.H., Bordalo-Rodrigues M., de Camargo O.P. (2014). Platelet-rich plasma in rotator cuff repair: A prospective randomized study. Am. J. Sports Med..

[124] Malavolta E.A., Gracitelli M.E.C., Assunção J.H., Ferreira Neto A.A., Bordalo-Rodrigues M., de Camargo O.P. (2018). Clinical and Structural Evaluations of Rotator Cuff Repair With and Without Added Platelet-Rich Plasma at 5-Year Follow-up: A Prospective Randomized Study. Am. J. Sports Med..

[125] Malik T., Mass D., Cohn S. (2016). Postoperative Analgesia in a Prolonged Continuous Interscalene Block Versus Single-Shot Block in Outpatient Arthroscopic Rotator Cuff Repair: A Prospective Randomized Study. Arthroscopy.

[126] Mardani-Kivi M., Keyhani S., Ebrahim-Zadeh M.H., Hashemi-Motlagh K., Saheb-Ekhtiari K. (2019). Rotator cuff tear with concomitant long head of biceps tendon (LHBT) degeneration: What is the preferred choice? Open subpectoral versus arthroscopic intraarticular tenodesis. J. Orthop. Traumatol..

[127] Mazzocca A.D., Arciero R.A., Shea K.P., Apostolakos J.M., Solovyova O., Gomlinski G., Wojcik K.E., Tafuto V., Stock H., Cote M.P. (2017). The Effect of Early Range of Motion on Quality of Life, Clinical Outcome, and Repair Integrity After Arthroscopic Rotator Cuff Repair. Arthroscopy.

[128] Menek B., Tarakci D., Algun Z.C. (2019). The effect of Mulligan mobilization on pain and life quality of patients with Rotator cuff syndrome: A randomized controlled trial. J. Back Musculoskelet Rehabil..

[129] Merivirta R., Äärimaa V., Aantaa R., Koivisto M., Leino K., Liukas A., Kuusniemi K. (2013). Postoperative fentanyl patch versus subacromial bupivacaine infusion in arthroscopic shoulder surgery. Arthroscopy.

[130] Milano G., Grasso A., Salvatore M., Saccomanno M.F., Deriu L., Fabbriciani C. (2010). Arthroscopic rotator cuff repair with metal and biodegradable suture anchors: A prospective randomized study. Arthroscopy.

[131] Milano G., Grasso A., Salvatore M., Zarelli D., Deriu L., Fabbriciani C. (2007). Arthroscopic rotator cuff repair with and without subacromial decompression: A prospective randomized study. Arthroscopy.

[132] Milano G., Saccomanno M.F., Careri S., Taccardo G., De Vitis R., Fabbriciani C. (2013). Efficacy of marrow-stimulating technique in arthroscopic rotator cuff repair: A prospective randomized study. Arthroscopy.

[133] Moosmayer S., Lund G., Seljom U., Svege I., Hennig T., Tariq R., Smith H.J. (2010). Comparison between surgery and physiotherapy in the treatment of small and medium-sized tears of the rotator cuff: A randomised controlled study of 103 patients with one-year follow-up. J. Bone Joint Surg. Br..

[134] Moosmayer S., Lund G., Seljom U.S., Haldorsen B., Svege I.C., Hennig T., Pripp A.H., Smith H.J. (2019). At a 10-Year Follow-up, Tendon Repair Is Superior to Physiotherapy in the Treatment of Small and Medium-Sized Rotator Cuff Tears. J. Bone Joint Surg. Am. Vol..

[135] Nam J.H., Park S., Lee H.R., Kim S.H. (2018). Outcomes After Limited or Extensive Bursectomy During Rotator Cuff Repair: Randomized Controlled Trial. Arthroscopy.

[136] Nicholas S.J., Lee S.J., Mullaney M.J., Tyler T.F., Fukunaga T., Johnson C.D., McHugh M.P. (2016). Functional Outcomes After Double-Row Versus Single-Row Rotator Cuff Repair: A Prospective Randomized Trial. Orthop. J. Sports Med..

[137] Nicholson J.A., Searle H.K.C., MacDonald D., McBirnie J. (2019). Cost-effectiveness and satisfaction following arthroscopic rotator cuff repair: Does age matter?. Bone Joint J..

[138] Oh C.H., Oh J.H., Kim S.H., Cho J.H., Yoon J.P., Kim J.Y. (2011). Effectiveness of subacromial anti-adhesive agent injection after arthroscopic rotator cuff repair: Prospective randomized comparison study. Clin. Orthop. Surg..

[139] Oh J.H., Kim J.Y., Choi J.H., Park S.M. (2014). Is arthroscopic distal clavicle resection necessary for patients with radiological acromioclavicular joint arthritis and rotator cuff tears? A prospective randomized comparative study. Am. J. Sports Med..

[140] Oh J.H., Kim J.Y., Chung S.W., Park J.S., Kim D.H., Kim S.H., Yun M.J. (2014). Warmed irrigation fluid does not decrease perioperative hypothermia during arthroscopic shoulder surgery. Arthroscopy.

[141] Oh J.H., Lee Y.H., Kim S.H., Park J.S., Seo H.J., Kim W., Park H.B. (2016). Comparison of Treatments for Superior Labrum-Biceps Complex Lesions With Concomitant Rotator Cuff Repair: A Prospective, Randomized, Comparative Analysis of Debridement, Biceps Tenotomy, and Biceps Tenodesis. Arthroscopy.

[142] Osbahr D.C., Cawley P.W., Speer K.P. (2002). The effect of continuous cryotherapy on glenohumeral joint and subacromial space temperatures in the postoperative shoulder. Arthroscopy.

[143] Osti L., Buono A.D., Maffulli N. (2015). Pulsed electromagnetic fields after rotator cuff repair: A randomized, controlled study. Orthopedics.

[144] Osti L., Del Buono A., Maffulli N. (2013). Microfractures at the rotator cuff footprint: A randomised controlled study. Int. Orthop..

[145] Pandey V., Bandi A., Madi S., Agarwal L., Acharya K.K., Maddukuri S., Sambhaji C., Willems W.J. (2016). Does application of moderately concentrated platelet-rich plasma improve clinical and structural outcome after arthroscopic repair of medium-sized to large rotator cuff tear? A randomized controlled trial. J. Shoulder Elb. Surg..

[146] Park J.Y., Bang J.Y., Oh K.S. (2016). Blind suprascapular and axillary nerve block for post-operative pain in arthroscopic rotator cuff surgery. Knee Surg. Sports Traumatol. Arth. Off. J. ESSKA.

[147] Park Y.B., Koh K.H., Shon M.S., Park Y.E., Yoo J.C. (2015). Arthroscopic distal clavicle resection in symptomatic acromioclavicular joint arthritis combined with rotator cuff tear: A prospective randomized trial. Am. J. Sports Med..

[148] Perdreau A., Joudet T. (2015). Efficacy of multimodal analgesia injection combined with corticosteroids after arthroscopic rotator cuff repair. Orthop. Traumatol. Surg. Res..

[149] Piitulainen K., Häkkinen A., Salo P., Kautiainen H., Ylinen J. (2015). Does adding a 12-month exercise programme to usual care after a rotator cuff repair effect disability and quality of life at 12 months? A randomized controlled trial. Clin. Rehabil..

[150] Raab M.G., Rzeszutko D., O'Connor W., Greatting M.D. (1996). Early results of continuous passive motion after rotator cuff repair: A prospective, randomized, blinded, controlled study. Am. J. Orthop..

[151] Randelli P., Arrigoni P., Aliprandi A., Sdao S., Ragone V., D'Ambrosi R., Randelli F., Cabitza P., Banfi G. (2015). Repair versus shaving of partial-thickness articular-sided tears of the upper subscapularis tendon. A prospective randomized controlled trial. Joints.

[152] Randelli P., Arrigoni P., Ragone V., Aliprandi A., Cabitza P. (2011). Platelet rich plasma in arthroscopic rotator cuff repair: A prospective RCT study, 2-year follow-up. J. Shoulder Elb. Surg..

[153] Randelli P., Stoppani C.A., Zaolino C., Menon A., Randelli F., Cabitza P. (2017). Advantages of Arthroscopic Rotator Cuff Repair With a Transosseous Suture Technique: A Prospective Randomized Controlled Trial. Am. J. Sports Med..

[154] Raschhofer R., Poulios N., Schimetta W., Kisling R., Mittermaier C. (2017). Early active rehabilitation after arthroscopic rotator cuff repair: A prospective randomized pilot study. Clin. Rehabil..

[155] Rha D.W., Park G.Y., Kim Y.K., Kim M.T., Lee S.C. (2013). Comparison of the therapeutic effects of ultrasound-guided platelet-rich plasma injection and dry needling in rotator cuff disease: A randomized controlled trial. Clin. Rehabil..

[156] Roddey T.S., Olson S.L., Gartsman G.M., Hanten W.P., Cook K.F. (2002). A randomized controlled trial comparing 2 instructional approaches to home exercise instruction following arthroscopic full-thickness rotator cuff repair surgery. J. Orthop. Sports Phys. Ther..

[157] Rodeo S.A., Delos D., Williams R.J., Adler R.S., Pearle A., Warren R.F. (2012). The effect of platelet-rich fibrin matrix on rotator cuff tendon healing: A prospective, randomized clinical study. Am. J. Sports Med..

[158] Ruiz-Moneo P., Molano-Muñoz J., Prieto E., Algorta J. (2013). Plasma rich in growth factors in arthroscopic rotator cuff repair: A randomized, double-blind, controlled clinical trial. Arthroscopy.

[159] Salviz E.A., Xu D., Frulla A., Kwofie K., Shastri U., Chen J., Shariat A.N., Littwin S., Lin E., Choi J. (2013). Continuous interscalene block in patients having outpatient rotator cuff repair surgery: A prospective randomized trial. Anesth. Analg..

[160] Schwartzberg R.S., Reuss B.L., Rust R. (2013). Efficacy of continuous subacromial bupivacaine infusion for pain control after arthroscopic rotator cuff repair. J. Shoulder Elb. Surg..

[161] Senekovic V., Poberaj B., Kovacic L., Mikek M., Adar E., Markovitz E., Maman E., Dekel A. (2017). The biodegradable spacer as a novel treatment modality for massive rotator cuff tears: A prospective study with 5-year follow-up. Arch. Orthop. Trauma Surg..

[162] Seven M.M., Ersen O., Akpancar S., Ozkan H., Turkkan S., Yıldız Y., Koca K. (2017). Effectiveness of prolotherapy in the treatment of chronic rotator cuff lesions. Orthop. Traumatol. Surg. Res..

[163] Shams A., El-Sayed M., Gamal O., Ewes W. (2016). Subacromial injection of autologous platelet-rich plasma versus corticosteroid for the treatment of symptomatic partial rotator cuff tears. Eur. J. Orthop. Surg. Traumatol..

[164] Sheps D.M., Bouliane M., Styles-Tripp F., Beaupre L.A., Saraswat M.K., Luciak-Corea C., Silveira A., Glasgow R., Balyk R. (2015). Early mobilisation following mini-open rotator cuff repair: A randomised control trial. Bone Joint J..

[165] Sheps D.M., Silveira A., Beaupre L., Styles-Tripp F., Balyk R., Lalani A., Glasgow R., Bergman J., Bouliane M., Shoulder and Upper Extremity Research Group of Edmonton (SURGE) (2019). Early Active Motion Versus Sling Immobilization After Arthroscopic Rotator Cuff Repair: A Randomized Controlled Trial. Arthroscopy.

[166] Shibata Y., Midorikawa K., Emoto G., Naito M. (2001). Clinical evaluation of sodium hyaluronate for the treatment of patients with rotator cuff tear. J. Shoulder Elb. Surg..

[167] Shin S.J. (2012). A comparison of 2 repair techniques for partial-thickness articular-sided rotator cuff tears. Arthroscopy.

[168] Syed U.A.M., Aleem A.W., Wowkanech C., Weekes D., Freedman M., Tjoumakaris F., Abboud J.A., Austin L.S. (2018). Neer Award 2018: The effect of preoperative education on opioid consumption in patients undergoing arthroscopic rotator cuff repair: A prospective, randomized clinical trial. J. Shoulder Elb. Surg..

[169] Takada M., Fukusaki M., Terao Y., Yamashita K., Ando Y., Sumikawa K. (2009). Postoperative analgesic effect of preoperative intravenous flurbiprofen in arthroscopic rotator cuff repair. J. Anesth..

[170] Tetzlaff J.E., Brems J., Dilger J. (2000). Intraarticular morphine and bupivacaine reduces postoperative pain after rotator cuff repair. Reg. Anesth. Pain Med..

[171] Thackeray E.M., Swenson J.D., Gertsch M.C., Phillips K.M., Steele J.W., Burks R.T., Tashjian R.Z., Greis P.E. (2013). Diaphragm function after interscalene brachial plexus block: A double-blind, randomized comparison of 0.25% and 0.125% bupivacaine. J. Shoulder Elb. Surg..

[172] Tirefort J., Schwitzguebel A.J., Collin P., Nowak A., Plomb-Holmes C., Ladermann A. (2019). Postoperative Mobilization After Superior Rotator Cuff Repair: Sling Versus No Sling: A Randomized Prospective Study. J. Bone Joint Surg. Am. Vol..

[173] Torrens C., Miquel J., Santana F. (2019). Do we really allow patient decision-making in rotator cuff surgery? A prospective randomized study. J. Orthop. Surg. Res..

[174] van der Zwaal P., Thomassen B.J., Nieuwenhuijse M.J., Lindenburg R., Swen J.W., van Arkel E.R. (2013). Clinical outcome in all-arthroscopic versus mini-open rotator cuff repair in small to medium-sized tears: A randomized controlled trial in 100 patients with 1-year follow-up. Arthroscopy.

[175] Walsh M.R., Nelson B.J., Braman J.P., Yonke B., Obermeier M., Raja A., Reams M. (2018). Platelet-rich plasma in fibrin matrix to augment rotator cuff repair: A prospective, single-blinded, randomized study with 2-year follow-up. J. Shoulder Elb. Surg..

[176] Wang A., McCann P., Colliver J., Koh E., Ackland T., Joss B., Zheng M., Breidahl B. (2015). Do postoperative platelet-rich plasma injections accelerate early tendon healing and functional recovery after arthroscopic supraspinatus repair? A randomized controlled trial. Am. J. Sports Med..

[177] Watanabe K., Tokumine J., Yorozu T., Moriyama K., Sakamoto H., Inoue T. (2016). Particulate-steroid betamethasone added to ropivacaine in interscalene brachial plexus block for arthroscopic rotator cuff repair improves postoperative analgesia. BMC Anesthesiol..

[178] Weber S.C., Kauffman J.I., Parise C., Weber S.J., Katz S.D. (2013). Platelet-rich fibrin matrix in the management of arthroscopic repair of the rotator cuff: A prospective, randomized, double-blinded study. Am. J. Sports Med..

[179] Yamakado K. (2014). Efficacy of arthroscopically placed pain catheter adjacent to the suprascapular nerve (continuous arthroscopically assisted suprascapular nerve block) following arthroscopic rotator-cuff repair. Open. Access J. Sports Med..

[180] Yamamoto S., Yamaguchi H., Sakaguchi M., Yamashita S., Satsumae T. (2003). Preoperative droperidol improved postoperative pain relief in patients undergoing rotator-cuff repair during general anesthesia using intravenous morphine. J. Clin. Anesth..

[181] Yun M.J., Oh J.H., Yoon J.P., Park S.H., Hwang J.W., Kil H.Y. (2012). Subacromial patient-controlled analgesia with ropivacaine provides effective pain control after arthroscopic rotator cuff repair. Knee Surg. Sports Traumatol. Arth. Off. J. ESSKA.

[182] Zhang Q., Zhou J., Ge H., Cheng B. (2015). Tenotomy or tenodesis for long head biceps lesions in shoulders with reparable rotator cuff tears: A prospective randomised trial. Knee Surg. Sports Traumatol. Arth. Off. J. ESSKA.

[183] Zumstein M.A., Rumian A., Thélu C., Lesbats V., O'Shea K., Schaer M., Boileau P. (2016). SECEC Research Grant 2008 II: Use of platelet- and leucocyte-rich fibrin (L-PRF) does not affect late rotator cuff tendon healing: A prospective randomized controlled study. J. Shoulder Elb. Surg..

[184] Urwin M., Symmons D., Allison T., Brammah T., Busby H., Roxby M., Simmons A., Williams G. (1998). Estimating the burden of musculoskeletal disorders in the community: The comparative prevalence of symptoms at different anatomical sites, and the relation to social deprivation. Ann. Rheum. Dis..

[185] Roquelaure Y., Ha C., Leclerc A., Touranchet A., Sauteron M., Melchior M., Imbernon E., Goldberg M. (2006). Epidemiologic surveillance of upper-extremity musculoskeletal disorders in the working population. Arth. Rheum..

[186] Meislin R.J., Sperling J.W., Stitik T.P. (2005). Persistent shoulder pain: Epidemiology, pathophysiology, and diagnosis. Am. J. Orthop..

[187] Tashjian R.Z. (2011). AAOS clinical practice guideline: Optimizing the management of rotator cuff problems. J. Am. Acad. Orthop. Surg..

[188] Beard D.J., Rees J.L., Cook J.A., Rombach I., Cooper C., Merritt N., Shirkey B.A., Donovan J.L., Gwilym S., Savulescu J. (2018). Arthroscopic subacromial decompression for subacromial shoulder pain (CSAW): A multicentre, pragmatic, parallel group, placebo-controlled, three-group, randomised surgical trial. Lancet.

[189] Karjalainen T.V., Jain N.B., Page C.M., Lahdeoja T.A., Johnston R.V., Salamh P., Kavaja L., Ardern C.L., Agarwal A., Vandvik P.O. (2019). Subacromial decompression surgery for rotator cuff disease. Cochrane Database Syst. Rev..

