# Supplementary material for: A Historical Analysis of Randomized Controlled Trials in Rotator Cuff Tears
Source: Int J Environ Res Public Health. 2020 Sep 20;17(18):6863. doi: 10.3390/ijerph17186863 (PMC7558823; doi:10.3390/ijerph17186863)
Supplement: Supplementary file 1 [file ijerph-17-06863-s001.pdf]

Supplementary Materials

Table S1. Acromioplasty

AUTHOR	YEAR	CONSORT	AIM	FOLLOW-UP	COLEMAN SCORE	RESULT	REHABILITATION
				ACROMIOPLASTY			
ABRAMS ***[1]***	2014	YES	To report the short-term clinical outcomes of patients undergoing arthroscopic repair of full-thickness RC tears with and without acromioplasty	24 months	82	The results of this study demonstrate no difference in clinical outcomes after rotator cuff repair with or without acromioplasty at 2 years postoperatively.	It was instituted 1/ 2 weeks after surgery, passive motion only. Sling immobilization when patients were not performing physical therapy or a home exercise program was continued for 6 weeks after surgery. Active range of motion was begun at 6 weeks, and strengthening was deferred until 12 weeks postoperatively.
DEZALY ***[2]***	2011	NO	To compare clinical results for arthroscopic repair of RC tear and acromioplasty-tenotomy in patients aged over 60 years	12 months	73	The study demonstrated the interest of arthroscopic rotator cuff repair in patients aged over 60 years. The benefit of repair compared to isolated acromioplasty-tenotomy depended on tendon healing.	Postoperative course in both groups comprised early self-rehabilitation with partial immobilization in a simple sling for four weeks.
JACQUOT ***[3]***	2014	-	To compare surgical RC repair to simple decompression by acromioplasty and biceps tenotomy in patients older than 60 years of age	48 months	74	Arthroscopic rotator cuff repair provides better functional outcomes than simple decompression in patients older than 60 years and prevents cuff tear arthropathy with eccentric humeral head position in the medium term.	Partial immobilization was achieved by wearing a simple sling for 4 weeks. A passive self-rehabilitation program was taught to all patients on day 1, with simple oral analgesics. After 4 weeks, physiotherapy sessions were prescribed if needed.
MACDONALD ***[4]***	2011	NO	To compare functional and quality of-life indices and rates of revision surgery in arthroscopic RC repair with and without acromioplasty	24 months	85	There was no difference in functional and quality-of-life indices for patients who had rotator cuff repair with or without acromioplasty.	Passive or active-assisted shoulder range-of-motion exercises began 1 week after surgery. Active shoulder motion began at 8 weeks postoperatively with strengthening exercises and reintegration into normal activities at 12 weeks postoperatively.
MOHTADI ***[5]***	2008	NO	To evaluate the difference in disease-specific quality of life outcomes at 2 years between an open surgical repair (open) versus an arthroscopic	24 months	82	There was no difference in outcome at 1 and 2 years after surgery between the scope mini-open and open procedures. The quality of life of patients undergoing the arthroscopic acromioplasty with mini-open	Postoperatively, patients in the open and scope mini-open groups were placed in a shoulder immobilizer for 6 weeks. all patients were instructed to follow immediate passive range of motion exercises followed by active ROM exercises at 6 weeks and strengthening exercises starting at 8 weeks postoperatively.

AUTHOR	YEAR	CONSORT	AIM	FOLLOW-UP	COLEMAN SCORE	RESULT	REHABILITATION
				ACROMIOPLASTY			
			acromioplasty with mini-open repair for patients with full-thickness RC tears			rotator cuff repair improved statistically significantly and clinically at 3 months compared with the open group.	
SHIN SJ ***[6]***	2012	-	To compare arthroscopic RC repair with and without acromioplasty	35 months	69	Arthroscopic repair of small- to medium-sized rotator cuff tears provided pain relief and improved functional outcome with or without acromioplasty. Clinical outcomes were not significantly different, and acromioplasty may not be necessary in the operative treatment of patients with small- to medium-sized rotator cuff tears in the absence of acromial spurs.	A shoulder brace with 0° of external rotation and 30° of abduction was applied in all patients for four weeks or five weeks postoperatively. Pendulum exercises and gentle passive shoulder range-of-motion exercises commenced three days after surgery. Active- assisted shoulder range-of-motion exercises began after weaning from the brace, and resisted shoulder motion and strengthening exercises began at three months.

Table legend: RC: rotator cuff. MR: magnetic resonance.

Table S2. PRP – PRFM - platelet-leukocyte membrane – mesenchymal stem cell - augmentation

AUTHOR	YEAR	CONSORT	AIM	FOLLOW-UP	COLEMAN SCORE	RESULT	RIABILITATION
			PRP – PRFM **- PLATELET-LEUKOCYTE MEMBRANE – MESENCHYMAL STEM CELL - AUGMENTATION**				
CAI ***[7]***	2018	NO	Compare subacromial injections of normal saline (NS), Subacromial injection of Sodium Hyaluronate (SH), Subacromial injections of PRP and Subacromial injection of Sodium Hyaluronate, and PRP in the treatment of small to medium partial-thickness rotator cuff tears	12 months	82	This study provided the evidence of the efficacy of PRP injection in the healing of small to medium PTRCTs. Moreover, the combined injection of SH and PRP yielded a better clinical outcome than SH or PRP alone.	_
CASTRICINI ***[8]***	2011	YES	To compare rotator cuff repair without (n = 45) or with (n = 43)	16 months	80	The study does not support the use of autologous PRFM for augmentation of a double-row	The operated shoulder was immobilized for 3 weeks using a sling. Pendulum exercises were allowed starting from the first postoperative day. After that period, passive and

AUTHOR	YEAR	CONSORT	AIM	FOLLOW-UP	COLEMAN SCORE	RESULT	RIABILITATION
***PRP – PRFM* - PLATELET-LEUKOCYTE MEMBRANE – MESENCHYMAL STEM CELL - AUGMENTATION**							
			augmentation with autologous platelet-rich fibrin matrix (PRFM)			repair of a small or medium rotator cuff tear to improve the healing of the rotator cuff. The results are applicable to small and medium rotator cuff tears.	assisted active exercises were initiated for forward flexion and external rotation. After 6 weeks, patients began strengthening exercises of the RC and scapular stabilizer
D'AMBROSI ***[9]***	2016	NO	To evaluate the adjuvant effect of supplementation with PRP in the arthroscopic repair of full-thickness RC tears, compared to a control group treated with arthroscopic repair without PRP supplementation as regards pain and functionality	6 months	70	PRP leads to a reduction in pain during a short-term follow-up. Pain reduction allows for a more rapid recovery of mobilization and improvement in functionality.	The protocol provides immobilization of the arm in a brace, abducted to 20° for a month. From the 3week, pendular movements. From the 35th day, active assisted kinesis and active hydrokinses with progressive recovery of the ROM were started. At the sixth week, patients started muscle strengthening exercises
EBERT ***[10]***	2017	NO	To compare patients who underwent 2 ultrasound-guided injections of PRP to the tendon repair site at 7 and 14 days after double-row arthroscopic supraspinatus repair to patients without (control group)	42 months	73	Significant postoperative clinical improvements and high levels of patient satisfaction were observed in patients at the midterm review after supraspinatus repair. While pain-free, maximal abduction strength was greater in the midterm after PRP treatment, repeated applications of PRP delivered at 7 and 14 days after surgery provided no additional benefit to tendon integrity.	Postoperatively, all patients were immobilized in a sling for 6 weeks and underwent a standard rehabilitation program consisting of passive range of motion exercises. active assisted range of motion exercises and a progressive strengthening program.
FLURY ***[11]***	2016	NO	To investigate whether an intraoperative pure PRP injection, compared with a local anesthetic injection, improves patient-reported outcomes at 3 and 6 months after arthroscopic RC repair	24 months	83	Patients treated with pure PRP showed no significantly improved function at 3, 6, and 24 months after arthroscopic repair compared with control patients receiving ropivacaine; however, a similar pain reduction was documented in both groups.	All patients followed a standard postoperative physical therapy protocol comprising 3 phases: (1) 6 weeks of passive mobilization using an abduction pillow , (2) 4 weeks of active mobilization and coordination training, and (3) specific progressive resistance exercises for the operated shoulder.
GUMINA ***[12]***	2012	-	To evaluate the clinical and magnetic resonance imaging results of arthroscopic RC repair with and without the	13 months	83	The use of the platelet-leukocyte membrane in the treatment of rotator cuff tears improved repair integrity compared with repair without membrane.	Postoperatively, all patients used a sling with the shoulder internally rotated. Passive shoulder motion was initiated under supervision during the first week. The sling was removed at 4 weeks postoperatively and active-assisted motion was started at that time. Full active shoulder motion

AUTHOR	YEAR	CONSORT	AIM	FOLLOW-UP	COLEMAN SCORE	RESULT	RIABILITATION
***PRP – PRFM* - PLATELET-LEUKOCYTE MEMBRANE – MESENCHYMAL STEM CELL - AUGMENTATION**							
			use of platelet-leukocyte membrane in patients with a large postero-superior RC tear			However, the improvement in repair integrity was not associated with greater improvement in the functional outcome.	was allowed at six to eight weeks postoperatively, and strengthening exercises were initiated at fifteen weeks.
HOLTBY ***[13]***	2016	NO	To compare arthroscopic RC repair with and without PRP	6 months	75	The PRP biological augmentation for repair of small- to medium-sized rotator cuff tears has a short-term effect on perioperative pain without any significant impact on patient-oriented outcome measures or structural integrity of the repair compared with control group.	A shoulder immobilizer, removed for shower and exercise, was applied for 4 weeks. Early active-assisted forward flexion and pendulum motions were initiated on day 1 postoperatively. At 4 weeks, external rotation was added. Internal rotation and submaximal isometric exercises started at 6 weeks. Strength exercises against resistance were delayed until 12 weeks post-surgery.
ILHANLI ***[14]***	2015	NO	To assess the effectiveness of PRP injection in partial supraspinatus tears by comparing with physical therapy	12 months	64	When we compared with PT, PRP seemed to be a well-tolerated application whit the shoulder promising result in patients with chronic partial supraspinatus tears.	_
JO CH ***[15]***	2013	NO	To assess the efficacy of PRP comparing arthroscopic repair of large to massive rotator cuff tears PRP-augmented (PRP group) to conventional treatment (conventional group)	12 months	77	The application of PRP for large to massive rotator cuff repairs significantly improved structural outcomes, as evidenced by a decreased retear rate and increased CSA of the supraspinatus compared with repairs without PRP augmentation. While there was no significant difference in clinical outcomes after 1-year follow-up, better structural outcomes in the PRP group might suggest improved clinical outcomes at longer term follow-up.	The shoulder was immobilized for 4 to 6 weeks using an abduction brace Shrugging, protraction, and retraction of shoul- der girdles; intermittent exercise of the elbow, wrist, and hand; and external rotation of the arm to neutral with the brace were encouraged as tolerated, usually immediately after surgery. Further passive ROM and active-assisted ROM exercises were allowed after gradual weaning off the abduction brace from 4 to 6 weeks after surgery. Patients began strengthening exercises after 3 months.

AUTHOR	YEAR	CONSORT	AIM	FOLLOW-UP	COLEMAN SCORE	RESULT	RIABILITATION
***PRP – PRFM* - PLATELET-LEUKOCYTE MEMBRANE – MESENCHYMAL STEM CELL - AUGMENTATION**							
JO CH ***[16]***	2015	NO	To assess the efficacy of PRP comparing arthroscopic repair of large to massive rotator cuff tears PRP-augmented (PRP group) to conventional treatment (conventional group)	12 months	79	Compared with repairs without PRP augmentation, the current PRP preparation and application methods for medium to large rotator cuff repairs significantly improved the quality, as evidenced by a decreased retear rate and increased CSA of the supraspinatus, but not the speed of healing. However, further studies may be needed to investigate the effects of PRP on the speed of healing without risking the quality.	The shoulder was immobilized for 4 weeks using an abduction brace. Shrugging, protraction, and retraction of shoulder girdles; Further passive range of motion (ROM) and active assisted ROM exercises were allowed after the patient was gradually weaned off the abduction brace from 4 to 6 weeks after surgery. Patients began strengthening exercises after 3 months.
MALAVOLTA ***[17]***	2014	NO	To compare arthroscopic RC repair with and without PRP injection	24 months	77	Platelet-rich plasma prepared by apheresis and applied in the liquid state with thrombin did not promote better clinical results at 24-month follow-up. Given the numbers available for analysis, the retear rate also did not change.	The shoulder was immobilized for 6 weeks. No motion was allowed during the first 3 weeks; passive exercise was permitted thereafter. Active assisted and active free exercises were started after week 6, when sling use was discontinued. Muscle strengthening was started at week 12.

AUTHOR	YEAR	CONSORT	AIM	FOLLOW-UP	COLEMAN SCORE	RESULT	RIABILITATION
***PRP – PRFM* - PLATELET-LEUKOCYTE MEMBRANE – MESENCHYMAL STEM CELL - AUGMENTATION**							
MALAVOLTA ***[18]***	2018	NO	To compare single-row repair of small to medium supraspinatus tears with PRP applied in the tendon-to-bone interface to arthroscopic RC repair without PRP	60 months	70	PRP obtained by apheresis and applied in a liquid form with the addition of thrombin at the end of single-row repair of supraspinatus tears did not promote better clinical or structural result at 60- months follow up	The shoulder was immobilizated for 6 weeks. No motion was allowed during the first 3 week, passive exercise was permitted. Acive- assisted and active free exercise were started after 6 weeks, when sling use was discontinued. Muscle strengthening was started at 12 weeks
PANDEY ***[19]***	2016	NO	To compare single-row repair with PRP application to single-row repair without PRP	24 months	84	Application of moderately concentrated PRP improves clinical and structural outcome in large cuff tears. PRP also enhances vascularity around the repair site in the early phase.	The shoulder was immobilized for 4 weeks in an arm sling. During the first 4 weeks, patients were encouraged to perform elbow, wrist, and finger mobilization and isometric scapular exercises. From 4 weeks onward, gradual passive mobilization of the shoulder was permitted. Active assisted mobilization of the shoulder was initiated between 6 and 8 weeks and strengthening of the cuff only after 3 months. Return to full activity was allowed 6 to 9 months after the restoration of strength and movement.

AUTHOR	YEAR	CONSORT	AIM	FOLLOW-UP	COLEMAN SCORE	RESULT	RIABILITATION
***PRP – PRFM* - PLATELET-LEUKOCYTE MEMBRANE – MESENCHYMAL STEM CELL - AUGMENTATION**							
RANDELLI ***[20]***	2011	NO	To compare RC repair with PRP application to RC repair without PRP	24 months	79	The results of our study showed autologous PRP reduced pain in the first postoperative months. The long-term results of subgroups of grade 1 and 2 tears suggest that PRP positively affected cuff rotator healing.	Patients were discharged the day after the operation wearing a sling. Patients were instructed to wear the sling continuously for 10 days postoperative. At postoperative day 10, passive assisted exercises were begun. Once passive, ROM was completely restored; and at a minimum of 30 days from the operation patients were allowed to start assisted active range-of-motion exercises. Selective strengthening exercises were started a minimum of 2 months postoperative.
RHA ***[21]***	2013	NO	To compare the effects of platelet-rich plasma injection with those of dry needling on shoulder pain and function in patients with RC disease	6 months	52	Autologous platelet-rich plasma injections lead to a progressive reduction in the pain and disability when compared to dry needling. This benefit is certainly still present at six months after treatment. These findings suggest that treatment with platelet-rich plasma injections is safe and useful for rotator cuff disease.	_

AUTHOR	YEAR	CONSORT	AIM	FOLLOW-UP	COLEMAN SCORE	RESULT	RIABILITATION
			PRP – PRFM **- PLATELET-LEUKOCYTE MEMBRANE – MESENCHYMAL STEM CELL - AUGMENTATION**				
RODEO ***[22]***	2012	NO	To compare RC repair with PRP application to RC repair without PRP	12 months	77	Platelet-rich fibrin matrix applied to the tendon-bone interface at the time of rotator cuff repair had no demonstrable effect on tendon healing, tendon vascularity, manual muscle strength, or clinical rating scales. In fact, the regression analysis suggests that PRFM may have a negative effect on healing. Further study is required to evaluate the role of PRFM in rotator cuff repair.	Patients began passive Codman and pendulum exercises within the first few days after surgery. Only passive motion was allowed during this initial phase. During the second 6-week period, the goal was to reestablish motion. Active-assisted elevation in the plane of the scapula was initiated at 6 weeks, followed by progression to active elevation. The third phase began at 12 weeks and emphasized strengthening of the rotator cuff and scapular muscles. Formal physical therapy typically continued for at least 4 to 5 months, followed by progression to a home exercise program.
RUIZ-MONEO ***[23]***	2013	NO	To compare RC repair with PRP application to RC repair without PRP	12 months	77	The present clinical trial does not support the use of plasma rich in growth factors in the arthroscopic repair of rotator cuff tears because no differences in rotator cuff healing or improvements in function were observed in the 1-year postsurgical clinical and radiological follow-up assessments	_

AUTHOR	YEAR	CONSORT	AIM	FOLLOW-UP	COLEMAN SCORE	RESULT	RIABILITATION
			***PRP – PRFM* - PLATELET-LEUKOCYTE MEMBRANE – MESENCHYMAL STEM CELL - AUGMENTATION**				
SHAMS ***[24]***	2016	NO	To evaluate the results of subacromial injection of platelet-rich plasma (PRP) versus corticosteroid injection therapy	6 months	55	PRP injections showed earlier better results as compared to corticosteroid injections, although statistically significant better results after 6 months could not be found. Therefore, subacromial RPP injection could be considered as a good alternative to corticosteroid injection, especially in patients with a contraindication to corticosteroid administration.	_
WALSH ***[25]***	2018	NO	To compare RC repair with autologous platelet-rich plasma fibrin matrix (PRPFM) application to RC repair without PRPFM	24 months	77	Our results showed no benefit from PRPFM used for rotator cuff repair according to the WORC Index, Simple Shoulder Test, and shoulder strength index.	Patients were discharged with a short period of immobilization (2 weeks), fol- lowed by passive motion until 6 weeks postoperatively. Isometric strengthening was introduced at 7-8 weeks, and full active- assisted range of motion and active range of motion were instituted at 10-12 weeks. Following 3 months of formal rehabilitation, the majority of the strengthening was done at home.

AUTHOR	YEAR	CONSORT	AIM	FOLLOW-UP	COLEMAN SCORE	RESULT	RIABILITATION
***PRP* – *PRFM* - PLATELET-LEUKOCYTE MEMBRANE – MESENCHYMAL STEM CELL - AUGMENTATION**							
WANG [26]	2015	YES	To compare postoperative and repeated application of platelet-rich plasma to the tendon repair site after double-row arthroscopic supraspinatus repair to double-row arthroscopic tendon repair	4 months	86	After arthroscopic supraspinatus tendon repair, image-guided PRP treatment on 2 occasions does not improve early tendon-bone healing or functional recovery.	All patients were immobilized in a sling for 6 weeks postoperatively and followed a standard rehabilitation program under the supervision of physical therapists. This consisted of passive range of motion exercises in the first 6 weeks, followed by active assisted range of motion exercises from 6 to 10 weeks, and finally, a guided strengthening program from 10 to 16 weeks.
WEBER ***[27]***	2013	NO	To compare rotator cuff repair without or with the use of platelet-rich fibrin matrix (PRFM)	12 months	74	Platelet-rich fibrin matrix was not shown to significantly improve perioperative morbidity, clinical outcomes, or structural integrity. While longer term follow-up or different platelet-rich plasma formulations may show differences, early follow-up does not show significant improvement in perioperative morbidity, structural integrity, or clinical outcome.	_

AUTHOR	YEAR	CONSORT	AIM	FOLLOW-UP	COLEMAN SCORE	RESULT	RIABILITATION
***PRP – PRFM* - PLATELET-LEUKOCYTE MEMBRANE – MESENCHYMAL STEM CELL - AUGMENTATION**							
ZUMSTEIN ***[28]***	2016	NO	To compare arthroscopic RC repair with leucocyte and platelet-rich fibrin (L-PRF) to arthroscopic RC repair without platelet-rich fibrin L-PRF	12 months	68	ArthroscopicrotatorcuffrepairwithapplicationofL-PRFyieldsnobeneficialeffectinclinical outcome, anatomic healing rate, mean postoperative defect size, and tendon quality at 12 months of follow-up.	Shoulders were immobilized postoperatively for 4 weeks after single-tendon tear repair and for 6 weeks after repair of massive tears of the supraspinatus and infraspinatus using a sling in adduction. Patients were advised to perform some exercises for 5 minutes for the first 3 weeks. Parallel during the immobilization period, they practice some passive motion exercises in the plane of the scapula at 3 weeks. Further passive range of motion and active-assisted range of motion exercises were allowed after gradual weaning off the sling from 4 to 6 weeks after surgery. No active motion was allowed for 6 weeks
BARBER ***[29]***	2012	NO	To evaluate the safety and effectiveness of arthroscopic acellular human dermal matrix augmentation of large RC tear repairs. Group 1 received acellular human dermal matrix augmentation. Group 2 doesn't receive it.	24 months	79	Acellular human dermal matrix augmentation of large cuff tears involving 2 tendons showed better ASES and Constant scores and more frequent intact cuffs as determined by gadolinium-enhanced MRI. Intact repairs were found in 85% of the augmented group and 40% of the nonaugmented group. No adverse events related to the acellular human dermal matrix were observed.	Postoperatively, patients' extremities were placed in an abduction sling for 4 to 6 weeks, allowing daily pendulum motion exercises. Supervised physical therapy was started at 4 weeks, with strengthening al- lowed starting at 12 weeks.

AUTHOR	YEAR	CONSORT	AIM	FOLLOW-UP	COLEMAN SCORE	RESULT	RIABILITATION
***PRP – PRFM* - PLATELET-LEUKOCYTE MEMBRANE – MESENCHYMAL STEM CELL - AUGMENTATION**							
CAI ***[30]***	2018	YES	To evaluate if Graft augmentation with 3D collagen could prevent re-tears of the repaired tendon and improve tendon-bone healing in moderate to large RC tears. Control group received suture-bridge technique. Study group received the suture-bridge technique augmented with 3-dimensional (3D) collagen.	12 months	75	3D collagen augmentation could provide effective treatment of moderate to large rotator cuff tears, providing substantial functional improvement, and could reduce the retear rate. This technique could also promote new tendon-bone formation, thus exerting a prominent effect on tendon-bone healing.	In the first week after surgery, the arm was maintained in a standard abduction pillow, during which pendulum exercises and gentle passive forward flexion were encouraged and started for all patients. Active assisted range of motion exercises were initiated at 6 weeks. After that, patients began active resistance muscle strengthening at the eighth week. From immediately after to 3 months after the operation, patients were allowed to practice light daily activities, whereas heavy manual work and sport activity were allowed only after 6 months postoperatively.
GREINER ***[31]***	2015	YES	To investigate the safety and feasibility of recombinant human bone morphogenetic protein 12 (rhBMP-12) on an absorbable collagen sponge (ACS) as an adjuvant therapy in open RC repair. Group 1 underwent open RC repair and .implantation of ACS with rhBMP-12. Group 2 underwent standard of care.	12 mounts	59	RSA with bony lateralization shows a trend toward improved external rotation in lateralized RSA, with a statistically significant improvement in external rotation in patients with an intact teres minor.	_

AUTHOR	YEAR	CONSORT	AIM	FOLLOW-UP	COLEMAN SCORE	RESULT	RIABILITATION
			PRP – PRFM **- PLATELET-LEUKOCYTE MEMBRANE – MESENCHYMAL STEM CELL - AUGMENTATION**				
AVANZI ***[32]***	2019	NO	To evaluate the anatomic integrity of rotator cuff repair performed by medialized single row and augmented by a porcine dermal patch, in comparison with a non-augmented group.	24 months	74	Rotator cuff repairs augmented with a porcine dermal patch resulted in excellent clinical outcomes with a higher healing rate and close-to-normal MRI findings. The technique is safe and effective; in addition, it is reproducible and allows for better outcomes compared with those of standard medialized single-row repairs.	A 4-month-long rehabilitation period was standardized for both groups and included a first phase focused on suture protection, a second phase focused on passive range of motion recovery, and a third phase focused on strength recovery and reconditioning.
LAMAS ***[33]***	2019	NO	To compare autologous mesenchymal stem cells to placebo in patients who underwent open rotator cuff repair.	12 months	71	Our study showed preliminary inconclusive clinical outcomes in the patients treated with MSCs. Adverse events revealed the need for further approaches using scaffolds of a different nature or perhaps no scaffolds, in the context of small joints.	The shoulder was immobilized postoperatively with an abduction sling.

Table legend: NS: normal saline; SH Sodium Hyaluronate; PRP: platelet rich plasma; : SH+PRP: Sodium Hyaluronate + platelet rich plasma. PRFM: platelet-rich fibrin matrix.

Table S3. Single row –double row

AUTHOR	YEAR	CONSORT	AIM	FOLLOW-UP	COLEMAN SCORE	RESULT	RHEABILITATION
			SINGLE ROW –DOUBLE ROW				
BURKS ***[34]***	2009	NO	To compare Arthroscopic Single and Double-Row RC Repair	12 months	71	No clinical or MRI differences were seen between patients repaired with a SR or DR technique.	Postoperatively all patients used an abduction sling. Under supervision, passive range of motion was started in the first week. Active assisted ROM was starting at 4 to 6 weeks postoperatively, and full active ROM was commenced at 6 to 8 weeks with the longer time periods for very large tears. Strengthening exercises were typically delayed for 10 to 12 weeks.
CARBONEL ***[35]***	2012	NO	To compare arthroscopic RC repair with single-row and double-row techniques.	24 months	86	At two years follow-up the double-row repair technique showed a significant difference in clinical out- come compared with single-row repair and this was even more significative in over 30-mm tears. No MRI differences were observed.	The arm was supported using an abduction sling. Under supervision, passive ROM was started in the first week. Active assisted ROM was typically started in a supine position starting at four to six weeks postoperatively, and full active ROM was commenced at six to eight weeks. Strengthening exercises were typically delayed for ten to 12 weeks.
FRANCESCHI ***[36]***	2016	YES	To investigate, in patients undergoing early accelerated mobilization, whether double-row (DR) repair provides better clinical outcomes and a lower re-tear rate compared with single-row (SR) configurations	24 months	73	In selected patients at a high risk of shoulder stiffness and therefore necessitating accelerated postoperative rehabilitation, DR repair of the RC could lower retear rates.	After surgery, each group followed an accelerated rehabilitation protocol; therefore, the rehabilitation program was the same for all patients.
FRANCESCHI ***[37]***	2007	YES	To evaluate the possible difference in clinical and imaging outcome between single-row and double-row suture anchor technique repairs of RC tears	24 months	78	Single- and double-row techniques provide comparable clinical outcome at 2 years. A double-row technique produces a mechanically superior construct compared with the single-row method in restoring the anatomical footprint of the rotator cuff, but these mechanical advantages do not translate into superior	The arm was supported using a sling with an abduction pillow for 6 weeks. Active elbow flexion and extension were allowed. Passive external rotation was started from the first day after surgery. At 6 weeks, the sling was removed, and overhead stretching with a rope and pulley was started. Isoinertial strengthening and rehabilitation of the rotator cuff, deltoid, and scapular stabilizers were initiated at 10 or 12 weeks after the operation

AUTHOR	YEAR	CONSORT	AIM	FOLLOW-UP	COLEMAN SCORE	RESULT	RHEABILITATION
				SINGLE ROW –DOUBLE ROW			
						clinical performance.	
GARTSMAN ***[38]***	2013	NO	To compare the structural outcomes of a single-row RC repair and double-row suture bridge fixation after arthroscopic repair of a full-thickness supraspinatus RC tear	10 months (range 6-12 months)	83	Arthroscopic double-row suture bridge repair of an isolated supraspinatus rotator cuff tear resulted in a significantly higher tendon healing rate when compared to arthroscopic single-row repair.	The patients were immobilized in an abduction sling for 6 weeks. Active shoulder elevation and abduction movements were forbidden. Active range of motion (ROM) of the fingers, wrist, and elbow was encouraged. The patients' only shoulder exercise was pendulum circumduction. The patient returned 6 weeks after the operation. The sling was discontinued and the patient instructed in a home program of supine active assisted ROM exercises in elevation
GRASSO ***[39]***	2009	NO	To compare the clinical outcome of arthroscopic RC repair with single-row and double-row techniques	24 months	66	At short-term follow-up, arthroscopic rotator cuff repair with the double-row technique showed no significant difference in clinical outcome compared with single-row repair.	After surgery, a sling was applied to the operated limb and was maintained for 3 weeks. After 8 weeks after surgery they start a ROM program, 12 weeks after surgery they started a muscle- strengthening program.
KIM ***[40]***	2016	YES	To compare clinical outcomes between conventional en masse repair and separate double-layer double-row repair for the treatment of delaminated RC tears	25.9 +/- 2 months	73	Both conventional en masse repair and separate double-layer double-row repair were effective in improving clinical outcomes in the treatment of delaminated rotator cuff tears. Lower pain scores were seen in patients who underwent separate double-layer double-row repair.	An abduction brace was applied for 1 month postoperatively, and pulley exercises were pre-scribed to increase forward flexion 1 month postoperatively. When passive shoulder range of motion (ROM) was restored to 90%, isometric exercises in all the planes were recommended. Thera-and exercises, strengthening exercises for the muscles to stabilize the scapula, and advanced muscle strengthening exercises with dumbbells were taught.

AUTHOR	YEAR	CONSORT	AIM	FOLLOW-UP	COLEMAN SCORE	RESULT	RHEABILITATION
			SINGLE ROW –DOUBLE ROW				
KOH KH ***[41]***	2011	YES	To compare the clinical outcomes and the re-tear rates of arthroscopic single-row and double-row suture anchor repair in 2 to 4 cm RC tears	24 months	84	This study indicates that the clinical results and retear rates of DR repair with 1 additional medial suture anchor were not significantly different from those of SR repairs with 2 lateral suture anchors in patients with medium to large rotator cuff tear.	An abduction brace was applied for protection for 3 weeks with an allowance for elbow and finger exercises. Passive range-of-motion exercises were started at the fourth week after surgery. Active range-of-motion exercises and rehabilitation were started after full passive range of motion had been acquired. Strengthening exercises with elastic bands were started after 3 months postoperatively
LAPNER ***[42]***	2012	NO	To compare the functional outcomes and healing rates after use of single-row and double-row suture techniques for repair of the RC	24 months	85	No significant differences in functional or quality-of-life outcomes were identified between single-row and double-row fixation techniques. A smaller initial tear size and a double-row fixation technique were associated with higher healing rates as assessed with ultrasonography or MRI.	Pendulum exercises were initiated on the first postoperative day. Active-assisted shoulder motion exercises were initiated six weeks postoperatively. Active motion began between eight and twelve weeks postoperatively, with strengthening exercises and reintegration into normal activities starting at twelve weeks postoperatively.

AUTHOR	YEAR	CONSORT	AIM	FOLLOW-UP	COLEMAN SCORE	RESULT	RHEABILITATION
				SINGLE ROW –DOUBLE ROW			
MA ***[43]***	2012	-	To compare the clinical and imaging outcomes of a single-row and double-row suture anchor fixation in arthroscopic RC repair with emphasis on analysis of the effect of various tear size on repair integrity	24 months	70	Arthroscopic rotator cuff repair with double-row fixation showed better shoulder strength in patients with larger tear size in comparison with single-row fixation. However, the imaging results showed no significant difference in cuff integrity in both groups in patients with any tear size at 6-month and minimum 2-year follow-up.	In the first week after surgery, an arm sling was used by all patients and gentle passive forward flexion started. Active range-of-motion exercise started at the sixth week for smaller tears and the eighth week for larger tears. Active resistance muscle strengthening exercise started at the eighth week for smaller tears and the tenth week for larger
NICHOLAS ***[44]***	2016	YES	To examine the effect of double-row versus single-row RC repair on functional outcomes and strength recovery in patients with full-thickness tears	36 months	70	Outcomes were not different between SR or DR repair, with generally excellent outcomes for both groups. Rotator cuff repair and subsequent rehabilitation markedly improved shoulder strength.	For the first 4 weeks, the affected upper extremity was placed in an abduction sling that was removed 3 times daily for pendulum exercises. Passive ROM was done for the first 6 weeks. Scapular mobility and stability exercises were initiated at this time. From the fourth week, passive ROM was extended After 6 weeks, passive motion was increased as tolerated. Active motion was encouraged as well as specific training of the rotator cuff in the scapular plane before progressing to the frontal and sagittal planes. Abduction strengthening was initiated 10 and 12 weeks postoperatively

AUTHOR	YEAR	CONSORT	AIM	FOLLOW-UP	COLEMAN SCORE	RESULT	RHEABILITATION
			SINGLE ROW –DOUBLE ROW				
IANNOTTI ***[45]***	2006	NO	To determine the effect of augmentation with small intestine submucosal patch compared with no augmentation open repairs of chronic two-tendon (supraspinatus and infraspinatus) tears of the RC. Thirty shoulders with a chronic two-tendon rotator cuff tear that was completely repairable with open surgery were randomized to be treated with either augmentation with porcine small intestine mucosa (augmentation group) or no augmentation (control group).	14 months	68	Augmentation of the surgical repair of large and massive chronic rotator cuff tears with porcine small intestine submucosa did not improve the rate of tendon-healing or the clinical outcome scores. On the basis of these data, we do not recommend using porcine small intestine submucosa to augment repairs of massive chronic rotator cuff tears done with the surgical and postoperative procedures described in this study.	The shoulder was immobilized in a sling for one week postoperatively, and only passive forward flexion and external rotation were allowed for eight weeks. The patient then progressed to active shoulder motion. Strengthening was begun at ten to twelve weeks postoperatively.
IDE ***[46]***	2017	YES	To assess functional recovery and structural outcomes in detail after implanting recombinant human bone morphogenetic protein–12 (rhBMP-12)/absorbable collagen sponge (ACS) as adjuvant treatment during open RC repair in patients over a 1-year postoperative follow-up	12 months	73	Functional recovery and structural outcomes in patients in whom rhBMP-12/ACS was used as adjuvant therapy in rotator cuff repair justify conducting future, larger, multicenter, prospective studies.	_

Table S4 Pain

AUTHOR	YEAR	CONSORT	AIM	FOLLOW-UP	COLEMAN SCORE	RESULT	RHEABILITATION
			PAIN				
*ALVAREZ **[47]***	2005	NO	To assess whether an injection of betamethasone is more effective than xylocaine alone in improving the quality of patients who have chronic RC tendinosis or partial RC tears	6 months	71	With the numbers available for this study, the authors found betamethasone to be no more effective in improving the quality of life, range of motion, or impingement sign than xylocaine alone in patients with chronic rotator cuff tendinosis for all follow-up time intervals evaluated.	_
*BANG **[48]***	2010	NO	To compare low-dose of gabapentin for postoperative pain management in patients undergoing arthroscopic RC repair to placebo	10 months	68	A single dose of 300 mg of gabapentin reduced the VAS score during the first 24 hours postoperatively in patients undergoing shoulder arthroscopic rotator cuff repair, without significant side effects when compared with placebo. However, the fentanyl consumption did not differ between the gabapentin and placebo groups	_
*BARBER **[49]***	2002	NO	To compare anesthetic continuous-infusion of bupivacaine for postoperative pain after outpatient shoulder surgery to saline solution	1 week	73	The first 2 postoperative days is the period of greatest pain. Continuous postoperative bupivacaine infusion is effective during this critical period, and the effect lingers even after the infusion is discontinued. The administration of bupivacaine via a continuous-infusion anesthetic pump statistically reduced postoperative pain after outpatient arthroscopic rotator cuff repairs, SLAP lesion repairs, subacromial decompressions, and capsular reefing.	_

AUTHOR	YEAR	CONSORT	AIM	FOLLOW-UP	COLEMAN SCORE	RESULT	RHEABILITATION
				PAIN			
BEHR ***[50]***	2012	NO	To compare epineural postoperative analgesia of middle interscalene brachial with levobupivacaine + intramuscular injection of saline (control group) to epineural levobupivacaine + intramuscular injection of buprenorphine 0.15 mg (IMB) to epineural levobupivacaine + epineural buprenorphine 0.15 mg + intramuscular injection of saline solution (ENB)	36 hours	60	Epineural buprenorphine prolonged postop- erative analgesia of MIB more effectively than intramuscular buprenorphine, which suggests that buprenorphine acts at a peripheral nervous system site of action.	_
BORGEAT ***[51]***	2010	YES	To compare the analgesia of a continuous infusion of ropivacaine 0.2% to of a continuous infusion of ropivacaine 0.3% administered through an interscalene catheter for the first 48 hours after surgery	1 month	70	The use of ropivacaine 0.3% through an interscalene catheter for the first 48 hours after open rotator cuff repair provided a significant reduction of morphine consumption and a better sleep quality for the first postoperative night without increasing the intensity of motor block or side effects	_
CHO CH ***[52]***	2015	NO	To compare multimodal analgesia + zolpidem to multimodal analgesia without zolpidem in patients undergoing arthroscopic RC repair	5 days	59	The use of zolpidem for analgesia after arthroscopic rotator cuff repair provided a significant reduction in the need for rescue analgesic without increasing adverse effects. Nevertheless, mean VAS pain scores during the first 5 days after surgery did not differ between the zolpidem group and the control group.	_

AUTHOR	YEAR	CONSORT	AIM	FOLLOW-UP	COLEMAN SCORE	RESULT	RHEABILITATION
			PAIN				
CHO CH ***[53]***	2011	NO	To compare the effectiveness and safety of a multimodal pain control protocol to intravenous patient-controlled analgesia in RC repair	5 days	59	The multimodal pain control protocol was found to offer more effective postoperative pain control with fewer adverse effects than intravenous patient-con- trolled analgesia. However, achieving adequate pain control within the first 48 h of surgery remains challenging, and thus, the developments of more effective and safer multimodal pain control protocols are required.	_
CHO NS ***[54]***	2007	NO	To compare Subacromial infusion of bupivacaine 0.5% to intravenous injection fentanil + ketorolac tromethamine	48 hours	64	Patient-controlled analgesia after arthroscopic rotator cuff repair showed that both subacromial infusion of bupivacaine and intravenous injection of fentanyl and ketorolac tromethamine were equally effective and clinically equivalent pain control methods.	_

AUTHOR	YEAR	CONSORT	AIM	FOLLOW-UP	COLEMAN SCORE	RESULT	RHEABILITATION
				PAIN			
CHOI ***[55]***	2018	NO	To compare interscalene brachial plexus block (IBPB) with general anesthesia before shoulder arthroscopy to general anesthesia without IBPB	-	75	Pre-operative IBPB with general anaesthesia for arthroscopic rotator cuff repair was beneficial in maintaining haemodynamic stability and improving the VCS during surgery.	_
CHOU ***[56]***	2010	NO	To compare injections of 25 mg/wk of sodium hyaluronate into the subacromial bursa for 5 consecutive weeks (ARTZ) to 2.5 mL of normal saline solution with the same injection protocol (placebo group) in patient with RC lesion without complete tears.	24 months	73	Subacromial injections of sodium hyaluronate are effective in treating rotator cuff lesions without complete tears.	_

AUTHOR	YEAR	CONSORT	AIM	FOLLOW-UP	COLEMAN SCORE	RESULT	RHEABILITATION
			PAIN				
COGHLAN ***[57]***	2009	NO	To compare ropivacaine infusion following RC repair and subacromial decompression (Group 1) to placebo infusion following RC repair and subacromial decompression (Group 2) to ropivacaine infusion following subacromial decompression (Group 3) to placebo infusion following subacromial decompression (Group 4)	24 hours	65	We found minimal evidence to support the use of ropivacaine infusion for improving outcomes following rotator cuff surgery in the setting of preemptive ropivacaine and intraoperative parecoxib.	_
CULEBRAS ***[58]***	2001	NO	To compare 3 groups. Placebo (n = 20): interscalene block with 40 mL of 0.5% bupivacaine with epinephrine (1/200000) and 1 mL of 0.9% saline, completed by 1 mL of 0.9% saline IM in the controlateral shoulder; Control (n = 20): interscalene block with 40 mL of 0.5% bupivacaine with epinephrine and 1 mL of 0. 9% saline, completed by 150 microg (=1 mL) of clonidine IM; Clonidine (n = 20): interscalene block with 40 mL of 0.5% bupivacaine with epinephrine and 150 microg (=1 mL) of clonidine, completed by 1 mL of 0.9% saline IM.	48 hours	56	The adding 150 µg of clonidine in interscalene block does not prolong analgesia induced by 40 mL of bupivacaine 0.5% with epinephrine, but de- creases mean arterial blood pressure and heart rate.	_

AUTHOR	YEAR	CONSOR T	AIM	FOLLOW-UP	COLEMAN SCORE	RESULT	RHEABILITATION
				PAIN			
*DELAUNAY **[59]***	2005	NO	To compare a continuous interscalene infusion of ropivacaine to a continuous infusion of subacromial ropivacaine	48 hours	46	After arthroscopic rotator cuff repair, continuous interscalene block provides better analgesia compared with continuous subacromial infusion but with an increased incidence of minor side effects	_
*DESROCHES **[60]***	2016	YES	To compare suprascapular nerve block (SSB) to interscalene block (ISB) as postoperative analgesia within the first 24 hour after arthroscopic supraspinatus and/or infraspinatus tendon repair	6 months	55	In this prospective, randomized controlled study, SSB was as effective as ISB for mean pain control within the first 24 hours but ISB was more effective in relieving pain in the recovery room after arthroscopic supraspinatus and/or infraspinatus tendon repair.	_

AUTHOR	YEAR	CONSOR T	AIM	FOLLOW-UP	COLEMAN SCORE	RESULT	RHEABILITATION
				PAIN			
GUMINA ***[61]***	2012	NO	To evaluate the clinical and MRI results of RC repair with and without the employment of this oral supplement of Tenosan in patients with a large, posterosuperior RC tear (RCT)	12 months	83	The use of the supplement for 3 months after cuff repair decreases shoulder post-operative pain and leads to a slight improvement in repair integrity. This improvement does not seem to correlate with an better objective functional outcome. However, these effects could facilitate and abbreviate the post-operative rehabilitation program and reduce re-rupture rate. The main limitations of this study are the relative short follow-up period and small number of patients studied.	_
HAN ***[62]***	2013	NO	To compare the effect of postoperative pain control and adverse effects of intravenous patient-controlled analgesia (IV PCA) to multimodal shoulder injection after arthroscopic RC repair	48 hours	57	Multimodal shoulder injection is a safe and effective modality for management of pain after arthro- scopic rotator cuff repair. Considering the expense and need of special devices for IV PCA, multimodal shoulder injection may be an effective and safe alternative to IV PCA for postoperative analgesia after arthroscopic rotator cuff repair.	The same rehabilitation protocol was applied to all patients for the duration of the study. Immobilization was maintained with an abduction brace while the shoulder remained motionless as instructed. Shrugging of both shoulders, gentle active elbow flexion/extension, gentle active forearm supination/pronation, and active hand plus wrist motion were encouraged immediately after surgery.

AUTHOR	YEAR	CONSORT	AIM	FOLLOW-UP	COLEMAN SCORE	RESULT	RHEABILITATION
				PAIN			
*HARTRICK **[63]***	2012	***YES***	To compare the effect of initial bolus of 5, 10, or 20 mL of ropivacaine 0.75% for continuous interscalene analgesia on postoperative pain and diaphragmatic function in patients undergoing arthroscopic shoulder surgery	3 months	75	ISB provided reliable surgical analgesia with 5 mL, 10 mL or 20 mL ropivacaine (0.75%). The 20 mL volume was associated with increased complaints of dyspnea. The 5 mL volume was associated with statistically higher pain scores in the immediate postoperative period. Lower volumes resulted in a reduced incidence of dyspnea compared to 20 mL, however diaphragmatic impairment was not eliminated. Compensatory increases in contralateral diaphragmatic movement may explain tolerance for ipsilateral paresis.	_

AUTHOR	YEAR	CONSORT	AIM	FOLLOW-UP	COLEMAN SCORE	RESULT	RHEABILITATION
				PAIN			
IKEMOTO **[64]**	2015	NO	To compare arthroscopic repair of the rotator cuff injury under general anesthesia in combination with interscalene block, without using an electrical nerve stimulating device (Group A) to general anesthesia in combination with suprascapular nerve block and infusion of anesthetic into the subacromial space moments before the surgical procedure (Group B) to general anesthesia alone, and only after the surgical procedure suprascapular nerve block and infusion of anesthetic into the subacromial space (Group C)	Mean follow-up: 39 mounts<br><br>Maximum follow-up: 72 mounts	75	Suprascapular nerve block with infusion of anesthetic into the subacromial space is an excellent alternative to interscalene block, particularly in hospitals in which an electrical nerve stimulating device is unavailable.	_
JO CH ***[65]***	2014	YES	To compare the effects of a multimodal analgesia (MMA) protocol which includes intra- and periarticular anesthesia versus saline-treated controls (Control) after arthroscopic rotator cuff repair.	72 hours	70	The MMA protocol used in this study was found to reduce postoperative pain and opioid consumption during the acute postoperative period after arthroscopic rotator cuff repair without increasing side effects after arthroscopic rotator cuff repair.	_

AUTHOR	YEAR	CONSORT	AIM	FOLLOW-UP	COLEMAN SCORE	RESULT	RHEABILITATION
				PAIN			
KHASHAN ***[66]***	2016	YES	To compare Group M: (morphine 20 mg/10 ml, intra-articuar), Group KM (ketamine 50 mg + morphine 10 mg/ 10 ml) and Group S (normal saline 0.9 % 10 ml.)	3 months	79	Pre-incisional intra-articular morphine reduced pain in the first 2 weeks after arthroscopic rotator cuff repair. Further research is warranted to elucidate the optimal timing and dosing of IA ketamine and morphine for postoperative analgesic effects.	_
KIM ***[67]***	2018	YES	To compare the efficacy of interscalene brachial plexus bolus blockade (IBPBB) to patient-controlled interscalene indwelling catheter analgesia (PCIA) to a control group for postoperative pain management within 48 hours postoperatively in patients undergoing arthroscopic RC repairs (ARCR)	48 hours	61	IBPBB provided effective immediate postoperative analgesia until 6 hours postoperatively. Especially until postoperative 2 hours, the VAS pain score was less than 1 point in the IBPBB group; however, there was significant rebound pain at 12 hours after surgery. During the first 24 hours postoperatively, PCIA reduced postoperative pain without rebound pain.	The shoulder was fixed with an abduction brace after the operation.

AUTHOR	YEAR	CONSORT	AIM	FOLLOW-UP	COLEMAN SCORE	RESULT	RHEABILITATION
				PAIN			
KIM ***[68]***	2015	NO	To compare postoperative analgesic efficacy and safety of continuous cervical epidural block (FCCEB) to ultrasound-guided continuous interscalene brachial plexus block with ropivacaine 0.75% (UCISB75) to ultrasound-guided continuous interscalene brachial plexus block with ropivacaine 0.20% (UCISB20)	12 months	77	UCISB20 may provide superior postoperative analgesia and is the most recommendable postoperative analgesic method in ARCR.	_
KO SH ***[69]***	2017	NO	To compare the results of arthroscopically guided suprascapular nerve block (SSNB) and blinded axillary nerve block with those of blinded SSNB in terms of postoperative pain and satisfaction within the first 48 hours after arthroscopic RC repair	48 hours	78	Arthroscopically guided SSNB and blinded axillary nerve block in arthroscopic rotator cuff repair for medium-sized rotator cuff tears provided more improvement in VAS for pain and greater patient satisfaction in the first 48 postoperative hours than blinded SSNB.	The cannula was removed at 48 hours after surgery. The operated shoulders were protected with the UltraSling for 6 postoperative weeks, and the same postoperative exercises were prescribed in both groups. An abduction pillow brace was used for shoulder immobilization in all patients postoperatively, and patients were cautioned to keep the shoulder in neutral rotation and 40° abduction.

AUTHOR	YEAR	CONSORT	AIM	FOLLOW-UP	COLEMAN SCORE	RESULT	RHEABILITATION
			PAIN				
KOH WU ***[70]***	2016	YES	To determine whether continuous supraclavicular block would provide postoperative analgesia comparable to that of continuous interscalene block	48 hours	68	The primary endpoint was the mean pain intensity 24 h after the surgery. Postoperative mean pain scores at 24 h were similar in the supraclavicular and interscalene groups. The incidence of complete or partial hemidiaphragmatic paresis was lower in the supraclavicular group Continuous supraclavicular block provided comparable analgesia compared with interscalene block with a reduced incidence of complete or partial hemidiaphragmatic paresis for 24 h following surgery.	_
LEE JJ ***[71]***	2017	NO	To compare the pain-relieving effect of ultrasound-guided interscalene brachial plexus block (ISB) combined with arthroscopy-guided suprascapular nerve block (SSNB) (Group 1) to ultrasound ISB alone (Group 2)	48 hours	77	Arthroscopy-guided SSNB combined with ultrasound-guided ISB resulted in lower visual analogue scale pain scores at 3–24 and 48 h post-operatively, and higher patient satisfaction scores at 6–36 h post-operatively with the attenuated rebound pain compared to scores in patients who received ultrasound-guided ISB alone after arthroscopic rotator cuff repair. The combined blocks may relieve post-operative pain more effectively than the single block within 48 h after arthroscopic cuff repair.	_

AUTHOR	YEAR	CONSOR T	AIM	FOLLOW-UP	COLEMAN SCORE	RESULT	RHEABILITATION
				PAIN			
LEE JJ ***[72]***	2014	NO	To compare the results of ultrasonographically guided axillary nerve block combined with suprascapular nerve block (Group 1) with those of suprascapular nerve block alone (Group 2) on postoperative pain and satisfaction	48 hours	77	Ultrasonographically guided ANB combined with SSNB in arthroscopic rotator cuff repair showed an improved mean VAS in the first 24 hours after surgery compared with SSNB alone. The mean SAT and LPI of the combined blocks were better than those of the single block within the first 36 hours. Ultrasonographically guided ANB combined with SSNB also decreased the rebound phenomenon	_
LEE JJ ***[73]***	2015	NO	To compare arthroscopy-guided direct suprascapular nerve block using 10 mL 0.5 % ropivacaine with 1:200,000 epinephrine performed after arthroscopic RC repair (Group 1) to arthroscopy-guided direct suprascapular nerve block using 10 mL 0.9 % saline after arthroscopic rotator cuff repair (Placebo)	24 hours	66	Post-operative need for fentanyl boluses as analgesia was reduced significantly, and it would be beneficial if this procedure involved a sensory branch of axillary nerve block or was performed at the beginning of the arthroscopic procedure. post-operative need for fentanyl boluses as analgesia was reduced significantly, and it would be beneficial if this procedure involved a sensory branch of axillary nerve block or was performed at the beginning of the arthroscopic procedure.	_

AUTHOR	YEAR	CONSORT	AIM	FOLLOW-UP	COLEMAN SCORE	RESULT	RHEABILITATION
				PAIN			
LIU ***[74]***	2016	NO	To compare the effect of a single dose interscalene block and general anesthesia (GA) to the effect of GA	3 days	68	After arthroscopic rotator cuff repair, an SISB effectively relieved pain on the day of surgery without any complications. In addition, insulin levels were significantly reduced at 42 hours postoperatively.	All patients followed the same rehabilitation protocol. Pendulum exercises were started 1 week post- operatively, and the abduction sling was removed and active assisted range of motion exercises were started 6 weeks postoperatively.
MALIK ***[75]***	2016	YES	To compare the analgesic efficacy of 3-day continuous interscalene brachial plexus block versus a single-shot block for arthroscopic RC repair	2 weeks	75	A 3-day continuous interscalene brachial plexus block provides better analgesia than a single- shot block. Sleep patterns were better, and less opioid was needed after arthroscopic rotator cuff repair in patients given a continuous plexus block.	_

AUTHOR	YEAR	CONSORT	AIM	FOLLOW-UP	COLEMAN SCORE	RESULT	RHEABILITATION
				PAIN			
MERIVIRTA ***[76]***	2013	NO	To compare the effectiveness of subacromial bupivacaine infusion and a transdermal fentanyl patch in the treatment of postoperative pain after arthroscopic shoulder surgery	3 months	70	A fentanyl patch delivering 12-mg/h fentanyl offers an easy and safe treatment option as a part of multimodal analgesia with few adverse effects in the treatment of postoperative pain in a carefully selected patient group after arthroscopic shoulder surgery	_
OH CH ***[77]***	2011	NO	To compare a subacromial injection of sodium hyaluronate (HA)/carboxymethylated cellulose (CMC) to a control group to evaluate the postoperative shoulder stiffness, pain and healing of RC repair, as well as the safety of an injection	12 months	65	A subacromial injection of an anti-adhesive agent after arthroscopic rotator cuff repair tended to produce faster recovery in forward flexion with no adverse effects on cuff healing. However, its anti-adhesive effects after rotator cuff repair should be considered carefully with further studies.	The immobilization period within the abduction brace was deter- mined by the tear size measured at the time of operation, 4 weeks for small 5 weeks for medium and 6 weeks for large to massive tears. Passive range of motion exercises were recommended immediately after surgery for small and medium sized tears but passive ROM exercises were restricted for 2 weeks for large to massive tears. Active ROM exercises were encouraged after weaning off the brace, and strengthening exercises were started 3 months after the operation

AUTHOR	YEAR	CONSORT	AIM	FOLLOW-UP	COLEMAN SCORE	RESULT	RHEABILITATION
				PAIN			
OSTI ***[78]***	2015	YES	To study whether the use of pulsed electromagnetic fields after RC repair is effective in the short term as an adjuvant treatment to reduce local inflammation, postoperative joint swelling, and recovery time, as well as to induce pain relief	26 months	69	Application of pulsed electromagnetic fields after rotator cuff repair is safe and reduces postoperative pain, analgesic use, and stiffness in the short term. At 2 years, no difference was seen in outcomes in patients who did or did not undergo treatment with pulsed electromagnetic fields.	Starting with pendular exercises, elbow passive flexion-extension, and scapular motion exercises on the day after surgery, and all patients wore a sling for 4 weeks. Passive range of motion exercises were started 2 weeks after surgery and continued for 4 weeks. Active exercises were started at 6 weeks, and strengthening exercises were allowed a minimum of 3 months after surgery.
PARK ***[79]***	2016	NO	To evaluate the efficacy of patient-controlled anesthesia (PCA) + additional axillary nerve block (ANB) + suprascapular nerve block (SSNB) to patient-controlled anesthesia (PCA) with no device assistance to PCA + SSN after arthroscopic RC repair	48 hours	66	PCA + SSNB + ANB is a cost-effective, time-saving, and easily performed method for post-operative pain control as an axis of multimodal pain control strategy.<br>PCA + SSNB + ANB is a cost-effective, time-saving, and easily performed method for post-operative pain control as an axis of multimodal pain control strategy.	_

AUTHOR	YEAR	CONSOR T	AIM	FOLLOW-UP	COLEMAN SCORE	RESULT	RHEABILITATION
				PAIN			
*SALVIZ **[80]***	2013	YES	To compare the recovery profile of patients receiving single injection (SISB) and continuous interscalene brachial plexus block (CISB) or general anesthesia (GA) for arthroscopic rotator cuff repair surgery through the first postoperative week	7 days	66	The analgesic benefits of CISB found in the PACU and immediately after discharge extend through the intermediate recovery period ending on postoperative day 7.	_
*SEVEN MM **[81]***	2017	NO	To compare dextrose prolotherapy injection to exercises to reduce pain and improve shoulder function and patient satisfaction	12 months	75	Prolotherapy is an easily applicable and satisfying auximiliary method in the treatment of chronic rotator cuff lesion.	Patients received a home exercise program described

AUTHOR	YEAR	CONSORT	AIM	FOLLOW-UP	COLEMAN SCORE	RESULT	RHEABILITATION
			PAIN				
TAKADA ***[82]***	2009	NO	To compare analgesic effects of lipid emulsion 0.1 ml·kg−1 as a placebo (Group A) to preoperative intravenous flurbiprofen 1 mg·kg−1(Group B) in patients undergoing arthroscopic RC repair under general anesthesia	24 hours	62	These results show that preoperative intravenous flurbiprofen facilitates the analgesic effect in the early postoperative period after arthroscopic rotator cuff repair.	_
TETZLAFF ***[83]***	2000	NO	To determine whether intra-articular injection of morphine, fentanyl, or sufentanil added to bupivacaine provided pain control after open RC repair. All received 20 mL of 0.25% bupivacaine: group 1, plain; group 2, with 1 mg of morphine added; group 3, with 50 microg of fentanyl added; and group 4, with 10 microg of sufentanil added	4 hours	65	Intra-articular injection of the shoulder with 0.25% bupivacaine and 1 mg morphine at the conclusion of surgery provided pain control and diminished morphine used in the first 24 hours after open rotator cuff repair. Fentanyl and sufentanil did not improve the analgesia over that achieved with bupivacaine alone	_

AUTHOR	YEAR	CONSORT	AIM	FOLLOW-UP	COLEMAN SCORE	RESULT	RHEABILITATION
				PAIN			
*THACKERAY **[84]***	2013	NO	To compare interscalene brachial plexus block (ISBPB) performed with 20 ml of 0,125% or 0,25% bupivacaine	48 hours	62	Function and oxygen saturation were superior in patients treated with more dilute bupivacaine. Furthermore, there were no clinically significant differences in pain scores, and no statistically significant differences in opioid requirements and patient satisfaction. Furthermore, there were no clinically significant differences in pain scores, and no statistically significant differences in opioid requirements and patient satisfaction.	_
*WATANABE **[85]***	2016	NO	To compare postoperative analgesic with ropivacaine (Group R) to betamethasone added to ropivacaine (Group BR) for brachial plexus block in patients who underwent arthroscopic RC repair	7 days	64	Betamethasone added to local anesthetic in interscalene brachial plexus block improved postoperative pain after arthroscopic rotator cuff repair, and betamethasone prolonged the duration of analgesia by almost 6 h.	_

AUTHOR	YEAR	CONSORT	AIM	FOLLOW-UP	COLEMAN SCORE	RESULT	RHEABILITATION
				PAIN			
Yamakado [86]	2014	NO	To compare arthroscopically placed perineural catheter at the scapular notch (ca-SSNB) to subacromial bursal block (SAB) in providing a continuous block of the suprascapular nerve	3 days	70	Ca-SSNB was highly effective in controlling postoperative pain after ARCR.	Postoperative physiotherapy was identical for both the groups. The operated shoulders were protected with an UltraSling (DJO Global, Vista, CA, USA) for 4–6 weeks, depending on the tear size.
YAMAMOTO ***[87]***	2003	NO	To demonstrate the effect of preoperative and intraoperative, small-dose intravenous (IV) droperidol on postoperative pain relief in orthopedic patients given general anesthesia with morphine comparing the pre-incision (P) group , the afterincision (A) group and the Control (C) group	18 hours	53	Preoperative IV droperidol resulted in improved postoperative pain relief inpatients undergoing shoulder rotator cuff surgery with general anesthesia using IV morphine	_

AUTHOR	YEAR	CONSOR T	AIM	FOLLOW-UP	COLEMAN SCORE	RESULT	RHEABILITATION
				PAIN			
YUN ***[88]***	2012	NO	To investigate the postoperative analgesic effect of subacromial patient-controlled analgesia with ropivacaine (SAPCA) in comparison with intravenous patient-con-trolled analgesia (IVPCA) after arthroscopic RC repair	48 hours	63	The analgesic effect of subacromial patient-controlled analgesia with ropivacaine was better than intravenous analgesia during the immediate postoperative period with fewer side effects.	_
MENEK ***[89]***	2019	NO	To compare Mulligan mobilization (Mulligan group) to physiotherapy (Control group) on pain and quality of life in individuals with Rotator cuff syndrome.	6 weeks	57	Mulligan mobilization was more effective than general treatment methods for pain as well as normal joint motion, DASH scoring and some parameters of SF-36 compared with general treatment methods.	After the participants were assessed, they were taken to the 6-week exercise program specifically once a day for 5 days a week.
COORY [90]	2019	YES	To compare the clinical efficacy of a suprascapular nerve block (ssnb) to subacromial injection (sa) in patients with symptomatic rotator cuff tears	3 months	61	This study demonstrates that an ssnb resulted in better pain and functional results than an sa at 6 and 12 weeks for symptomatic rotator cuff tears.	Each participant received standard advice regarding activity mod- ification and rotator cuff tear murdoch protocol physiotherapy. Patients were permitted to take nsaids on an as-required basis.

AKBABA ***[91]***	2019	NO	To evaluate the effectiveness of the treatment of active myofascial trigger points (MTrPs) in patients with rotator cuff pathologies. Patients were randomly divided into two groups. Group 1 received treatment of active MTrPs with ischemic compression (IC) of MTrPs in addition to standard conservative treatment program; patients in Group 2 received only the standard conservative treatment program.	1,5 months	50	A six-week course of IC helps treat active MTrPs. A standard conservative treatment program reduced pain and increased function; the addition of MTrP treatment did not improve clinical outcomes in patients with rotator cuff pathologies.	_

AUTHOR	YEAR	CONSORT	AIM	FOLLOW-UP	COLEMAN SCORE	RESULT	REHABILITATION
				PAIN			
BANERJEE ***[92]***	2008	NO	To evaluate the efficacy of continuous low dose bupivacaine infiltration by infusion pump into the subacromial space after arthroscopic RC repair. Group1 received 0.25% of bupivacaine at 2 mL/hr (n = 20), Group 2 received 0.25% bupivacaine at 5 mL/hr (n = 20), Group 3 received saline at 5 mL/hr	2 days	78	This study neither supports nor refutes the use of infusion pumps. Authors hypothesized that the placebo group would experience greater pain than the 5-mL group; however, a non significant trend toward the contrary occurred. A trend toward less pain in the 2-mL group was not significant.	Standard postoperative and rehabilitative protocols were followed. Pendulum and passive range of motion exercises were begun on postoperative day 2, at the time of the first therapy visit and pain pump removal. Active assisted exercises began on week 6 and active exercises were started at week 8. Strengthening began at 12 weeks.
GIALANELLA ***[93]***	2011	NO	To evaluate the effect of intraarticular injections of corticosteroids	6 months	65	Our study indicates that intraarticular injection of triamcinolone improves pain relief for 3 months in RCT and	_

AUTHOR	YEAR	CONSORT	AIM	FOLLOW-UP	COLEMAN SCORE	RESULT	REHABILITATION
			PAIN				
			(triamcinolone) in patients with symptomatic rotator cuff tears (RCT). Group1 received a single intraarticular injection of 40 mg triamcinolone, Group2 received two injections of 40 mg triamcinolone. Group 3 received no treatment (control group).			its action is not prolonged or potentiated by two injections of the drug done at 21-day intervals.	
LEE HJ ***[94]***	2015	YES	To compare the efficacy of local injection administered in the glenohumeral joint, subacromial space, or both location after arthroscopic RC repair. Group 1 received a postoperative glenohumeral injection of bupivacaine (20 mL) and lidocaine (10 mL). Group2 received the same postoperative injection in the subacromial space. Group3 received the same amount of local anesthesia but with half injected in the glenohumeral joint and half in the subacromial space.	1 day	71	Injection of local analgesics after arthroscopic rotator cuff repair relieves postoperative pain regardless of the injection location.	_
PERDREAU ***[95]***	2015	NO	To assess the short-term efficacy and safety of multimodal analgesic (MMA) injection associated to corticosteroids in arthroscopic RC tear surgery. The study group received	4.5 months	71	MMA associated to corticosteroids after arthroscopic rotator cuff tear surgery provided immediate benefit in terms of analgesia and morphine sparing, without apparent risk of infection. The practice is presently little known in France and deserves	Postoperative functional care comprised 1-month relative immobilization by elbow-to-body sling associated to immediate pendulum exercises according to preoperative rehabilitation advice. Thereafter, rehabilitation was intensified by the physiotherapist, following a specific program similar

AUTHOR	YEAR	CONSORT	AIM	FOLLOW-UP	COLEMAN SCORE	RESULT	REHABILITATION
PAIN							
			subacromial injection of a mixture of morphine, ropivacaine and methylprednisolone associated to intra-articular injection of morphine plus methylprednisolone; the control group received only isotonic saline.			longer-term assessment, especially as regards functional rehabilitation and tendon healing.	in all cases.
GANOKROJ ***[96]***	2019	NO	To compare the accuracy and effectiveness of the mid-lateral (Group 1) and posterior (Group 2) routes of subacromial injection	3 months	63	Univariate analysis showed no correlation between accuracy and age, sex, body mass index, or circumference of the proximal humerus. .However, injection route had some influence on accuracy, with a crude odds ratio of 5.41 for the midlateral route. Midlateral was the preferred route for subacromial injection.	_
SCHWARTZBERG ***[97]***	2013	NO	To evaluate the efficacy of continuous subacromial bupivacaine infusion to relieve pain after arthroscopic RC repair. Group 1 received no postoperative subacromial infusion catheter. Group 2 received a postoperative subacromial infusion catheter filled with saline solution. Group 3 received a postoperative subacromial infusion catheter filled with 0.5% bupivacaine without epinephrine.	6 months	67	The use of continuous bupivacaine subacromial infusion catheters resulted in no detectable pain reduction after arthroscopic rotator cuff repair based on visual analog scale scores and narcotic medication consumption.	_
DESMET ***[98]***	2015	YES	To evaluate the effect of three different doses	2 days	61	The median (IQR [range]) time to first postoperative	_

AUTHOR	YEAR	CONSO RT	AIM	FOLLOW-UP	COLEMAN SCORE	RESULT	REHABILITATION
				PAIN			
			of intravenous dexamethasone on the duration of interscalene blockade. Group 1 received saline 0.9%. Group 2 received 1.25 mg of dexamethason. Group 3 received 2.5 mg of dexamethasone Group 4 received 10 mg of dexamethasone			analgesic request after saline was 12.2 h, which was extended by intravenous dexamethasone 2.5 mg and 10 mg to 17.4 Postoperative analgesia was given sooner after rotator cuff repair than subacromial decompression, hazard ratio but later in older participants, hazard ratio per year but later in older participants, hazard ratio per year	
KIM ***[99]***	2017	NO	To compare the clinical efficacy of continuous infusion of remifentanil (Group 1), nicardipine, (Group 2) and remifentanil plus nicardipine (Group 3) to control hypotensive anesthesia in arthroscopic shoulder surgery	2 hours	65	he continuous infusion of remifentanil plus nicardipine appeared to be advantageous for maintaining hypotensive anesthesia until 120 min of arthroscopic shoulder surgery without rebound pain in a postanesthesia care unit.	_
SYED ***[100]***	2018	YES	To evaluate whether preoperative narcotics education reduces consumption after arthroscopic RC repair. Patients undergoing primary arthroscopic RC repair were randomized to receiving opioid- related preoperative education (Group 1) or not (Group 2).	3 months	72	The findings of this study determined that preoperative education intervention significantly decreased the number of narcotic pills consumed at 3 months after ARCR. In addition, education resulted in earlier cessation of opioids; therefore, directed patient education can help alleviate the current opioid epidemic.	_

Table S5 Conservative vs surgical

AUTHOR	YEAR	CONSORT	AIM	FOLLOW-UP	COLEMAN SCORE	RESULT	REHABILITATION
			CONSERVATIVE VS SURGICAL				
KIM ***[101]***	2018	YES	To compare the results of immediate arthroscopic RC repair to repair after 6 months of non-operative care in patients with partial rotator cuff tears involving more than 50% of the tendon thickness. Group 1 received immediate rotator cuff repair. Group 2 received delayed rotator cuff repair after 6 months of nonoperative treatment.	12 months	61	Both immediate surgical repair and delayed repair after nonsurgical care for PTRCTs were effective in improving clinical outcomes, and there was a very low incidence of retears in both groups. However, at 6 months postoperatively, superior functional outcomes were observed in the delayed repair group compared with the immediate repair group. A trial period of pre- operative nonsurgical care is reasonable, and immediate surgical repair is not crucial for the treatment of PTRCT.	An abduction brace was applied for 1 month after the operation. Pulley exercises were prescribed after 1 postoperative month. When the ROM was restored to 90%, isometric exercises in all planes were recommended. Band exercises, strengthening exercises for the muscles to stabilize the scapula, and advanced muscle strengthening exercises with dumbbells were taught.
MOOSMAYER ***[102]***	2019	NO	To compare the difference between Tendon Repair and Physiotherapy	120 months	88	At 10 years, the differences in outcome between primary tendon repair and physiotherapy for small and medium-sized rotator cuff tears had increased, with better results for primary tendon repair.	Treatment sessions of 40 minutes were given twice weekly for 12 weeks and with decreasing frequency during the following 6 to 12 weeks. No additional treatment measures such as anti-inflammatory or analgesic medication were given.
LAMBERS ***[103]***	2015	NO	To compare functional and radiologic improvement after surgical and conservative treatment of degenerative RC tears. Group1 underwent rotator cuff repair. Group 2 underwent conservative treatment.	12 months	67	In our population of patients with degenerative rotator cuff tears who were randomly treated by surgery or conservative protocol, we did not observe differences in functional outcome as measured with the CMS 1 year after treatment. The best outcomes in function and pain were seen in patients with an intact rotator cuff postoperatively.	After surgery, the patient wore a sling for 6 weeks. Patients were referred for physical therapy and treatment was commenced according to a standardized protocol.21 In the first 6 weeks, only passive movements were allowed. Passive GH movement was performed to prevent loss of mobility. The mobility of elbow and wrist was passively maintained. Circumduction exercises were allowed. After 6 weeks, active guided treatment was started and was expanded to active treatment. Strength development was started 3 months postoperatively.

AUTHOR	YEAR	CONSORT	AIM	FOLLOW-UP	COLEMAN SCORE	RESULT	REHABILITATION
				CONSERVATIVE VS SURGICAL			
KUKKONEN ***[104]***	2014	NO	To compare the outcome from surgical repair and physiotherapy. Patients were randomly allocated into one of three groups; Group 1 underwent physiotherapy, Group 2 acromioplasty and physiotherapy and Group 3 underwent rotator cuff repair, acromioplasty and physiotherapy.	12 months	78	These results suggest that at one-year follow-up, operative treatment is no better than conservative treatment with regard to non-traumatic supraspinatus tears, and that conservative treatment should be considered as the primary method of treatment for this condition.	The exercise protocol was standardised and started with exercises aimed at improving glenohumeral motion and active scapular retraction for the first six weeks<br>Then static and dynamic exercises for the scapular and glenohumeral musculature were gradually increased from six weeks to 12 weeks, after which the participant increased resistance and strength training up to six months.
MOOSMAYER ***[105]***	2010	NO	To compare surgical repair to physiotherapy in patients with small and medium size rotator cuff tears.	12 months	78	Analysis of between-group differences showed better results for the surgery group on the Constant scale<br>for pain-free abduction and for reduction in pain. for pain-free abduction and for reduction in pain	_

Table S6 Trauma-related rotator cuff tears

AUTHOR	YEAR	CONSORT	AIM	FOLLOW-UP	COLEMAN SCORE	RESULT	REHABILITATION
**TRAUMA-RELATED ROTATOR CUFF TEARS**							
AAGAARD ***[106]***	2019	NO	To investigate if early repair of trauma-related full-thickness rotator cuff tears could prevent healing failure.	12 months	64	Although early repair of trauma-related FTRCT improved patient relevant outcomes over two years for the entire cohort, only two out of three repaired rotator cuffs displayed intact structural integrity on MRI after one year. Consequently, early repair did not seem to prevent healing failure after trauma-related FTRCT.	Postoperatively, all patients underwent rehabilitation in primary care according to an evidence-based standardized protocol. During the first four weeks, passive range of movement (ROM) with elevation up to 145° was allowed. From the fifth week, the patients started with active assisted ROM. The active ROM started at week seven by patients with single tendon repairs and at week ten by those with multiple tendon repairs, followed by strengthening exercises.

Table S7 Physiotherapy

AUTHOR	YEAR	CONSORT	AIM	FOLLOW-UP	COLEMAN SCORE	RESULT	REHABILITATION
			PHYSIOTHERAPY				
AKBABA ***[107]***	2018	YES	To investigate the effect of setting expectations verbally on the effectiveness of kinesiotape application in patients with a RC tear. Group 1: there is no evidence that kinesiotaping is effective; Group 2 (here is limited evidence that kinesiotaping is effective; Group 3 there is evidence that kinesiotaping has an excellent effect.	24 hours	77	Setting positive expectations verbally about kinesiotaping might be effective in reducing pain in patients with rotator cuff tear.	_
ANALAN ***[108]***	2015	NO	To evaluate the effects of therapeutic continuous ultrasound (US) on patients with RC disease. The patients were randomly assigned to either a group that received true US (n=11) or one that received sham US (n=11).	3 weeks	64	In patients with rotator cuff disease, physiotherapy interventions effectively treat the pain, improve the clinical status, and increase the muscle strength of the shoulder ER at a low constant angular velocity, with functional improvement being seen immediately after treatment. However, at the end of the intervention, the US had yielded no additional efficacy to the physiotherapy treatment regimen of the patients with rotator cuff disease.	_
ARIAS-BURIA ***[109]***	2015	NO	To evaluate the effects of including 1 session of trigger point dry needling (TrP-DN) into a multimodal physiotherapy treatment on pain and function in postoperative shoulder	2 months	67	Our results suggest that including a single session of TrP-DN in the first week of a multimodal physical therapy approach may assist with faster increases in function in individuals with postoperative shoulder pain.	_

AUTHOR	YEAR	CONSORT	AIM	FOLLOW-UP	COLEMAN SCORE	RESULT	REHABILITATION
				PHYSIOTHERAPY			
			pain. Patients were divided in physiotherapy group, who received best evidence physical therapy and physical therapy plus TrP-DN group who received the same intervention plus a single session of TrP-DN.				
ARNDT ***[110]***	2012	NO	To compare the clinical results after two types of postoperative management: immediate passive motion versus immobilization. Patients were randomized to receive postoperative management of immediate passive motion or strict immobilization for 6 weeks.	15 months	80	Results for healing seemed to be slightly better with immobilization, but this was not statistically significant: the cuff had a normal appearance in 35.9% of cases after immobilization compared to 25.6% after passive motion, an image of intratendinous addition was found in 25.6% versus 30.2%, punctiform leaks in 23.1% versus 20.9%, and recurrent tears in 15.4% versus 23.3% respectively.	In the ‘‘Passive’’ group, rehabilitation began the day after surgery with three to five sessions per week depending upon the availability of the physical therapist. This included pendulum exercises, manual passive range of motion and work on a CPM machine without limitation of the range of motion. A sling was worn in between the rehabilitation sessions. The ‘‘Immobilization’’ group included strict immobilization of the shoulder in a sling that was placed at the end of surgery and worn for 6 weeks, with no movement allowed except pendulum exercises. Active rehabilitation was begun in both groups after the 6-week postoperative control, and was identical in both groups.
BAUMGARTEN ***[111]***	2016	YES	To compare patients that used pulleys 6 weeks postoperatively (Pulley Group), to patients that followed a rehabilitation protocol without pulleys (Jackins Group) after RC repair	6 months	73	A rotator cuff repair rehabilitation program that uses pulleys does not result in inferior outcomes, as determined by patient-determined outcome scores, measurements of scapular substitution, range of motion, and caption strength.	The rehabilitation was done only in pre-operative period
BLUM ***[112]***	2009	NO	To assess the effect of H-Wave electro stimulation device (HWDS) on range of	3 months	67	HWDS compared to PLACEBO induces a significant increase in range of motion in positive	post-operative rehabilitation it is standard procedure that in post-operative rotator cuff reconstruction patients are not allowed to lift the arm and they are fitted

AUTHOR	YEAR	CONSORT	AIM	FOLLOW-UP	COLEMAN SCORE	RESULT	REHABILITATION
			PHYSIOTHERAPY				
			motion and strength testing in patients who underwent RC reconstruction. Patients were randomly assigned into one of two groups: 1) H-Wave device stimulation (HWDS); 2) Sham-Placebo Device (PLACEBO).			management of rotator cuff reconstruction, supporting other previous research on HWDS and improvement in function. Interpretation of this preliminary investigation while suggestive of significant increases in Range of Motion of Post -Operative Rotator Cuff Reconstruction, warrants further confirmation in a larger double-blinded sham controlled randomized study	for a sling for a long period of time. In this study, no active Physical Therapy occurred in most cases for at least 8 weeks post surgery. each patient utilized the H- Wave device and program or sham and were allowed only passive Range of Motion (ROM).
CUFF ***[113]***	2012	NO	To evaluate patient outcomes and RC healing after arthroscopic RC repair using a postoperative physical therapy protocol. In the early group, 33 patients were randomized to passive elevation and rotation that began at postoperative day 2. In the delayed group, 35 patients began the same protocol at 6 weeks	12 months	68	Patients in the early group and delayed group both demonstrated very similar outcomes and range of motion at 1 year. There was a slightly higher rotator cuff healing rate in the delayed passive range of motion group compared with the early passive range of motion group	The physical therapy session was limited to 120 of forward elevation in the scapular plane and 30 of external rotation with the arm in adduction for the first 3 weeks.<br>After week 3, patients were progressed to passive forward elevation to tolerance and passive external rotation to 45 began active assisted range of motion with the therapist at 6 weeks, progressed to full active range of motion by 10 weeks, and began strengthening at 12 weeks.<br>Patients began active assisted range of motion with the therapist at 6 weeks, progressed to full active range of motion by 10 weeks, and began strengthening at 12 weeks.
DE ROO ***[114]***	2015	NO	To compare the clinical results of immediate passive mobilization versus delayed mobilization in the rehabilitation of RC repair during the early postoperative period. The mobilization group (79 patients) received	4 months	67	We noted no significant difference between the two groups regarding range of motion at 6 weeks and range of motion, strength and functional outcome scores at 4 months. Ultrasound didn't show a difference in healing at 6 w in either of both groups.	All patients received a postoperative abduction (30°) pillow brace for 4 weeks day and night and 2 more weeks only at night.<br>It was allowed to remove the pillow for hygienic purposes and 3 times a day to do some pendu lum exercises. The MO group started physical therapy at day 1 under guidance of their own physical therapist following a standardized

AUTHOR	YEAR	CONSORT	AIM	FOLLOW-UP	COLEMAN SCORE	RESULT	REHABILITATION
			PHYSIOTHERAPY				
			immediate daily passive mobilization. The immobilization group (51 patients) was immobilized for 4 weeks until physiotherapy was started			Both rehabilitation protocols seem applicable as well as safe in the early post-operative phase.	scheme with daily controlled passive mobilization exercises Specific capsular glenohumeral exercises and active-assisted shoulder exercises were started at week 5 progressively. At week 8, muscle strengthening was al- lowed progressively. The IM group was continuously immobilized in the brace for 4 weeks except for the pendulum exercises. Gradual passive mobilization was started from week 5 on a self-administered basis and after 6 weeks a similar protocol was used as group MO under guidance of their physical therapist.
*DUZGUN **[115]***	2011	NO	To compare the effects of the slow and accelerated protocols on pain and functional activity level after arthroscopic RC repair	16 weeks	69	The accelerated protocol is recommended to physical therapists during rehabilitation after arthroscopic rotator cuff repair to prevent the negative effects of immobilization and to support rapid reintegration to daily living activities.	The exercise program and manual therapy techniques are identical in these protocols, the timing differed. While active movement was initiated during week 3 in the accelerated protocol, it was started during week 6 in the slow protocol. While the accelerated protocol is completed in 8 weeks, the slow protocol is completed in 22 weeks
*DUZGUN **[116]***	2014	NO	To investigate the effects of the early initiation of passive and active range of motion exercises following arthroscopic RC repair. Patients were quasi-randomly assigned into accelerated (accelerate) protocol (n=19) and slow (slow) protocol (n=21) groups. Patients in both groups were treated with the same protocol.	24 months	67	The early initiation of passive and gentle controlled active motion exercise following rotator cuff repairs does not appear to affect range of motion in the first 6 postoperative months.	In the SLOW group, soft tissue mobilization for the scapulothoracic and glenohumeral joint along with passive ROM exercises were initiated at the 4th postoperative week. Active ROM in scapular plane elevation, flex- ion and abduction was initiated at the 6th week and light resistive elastic resistance exercises at the 8th week. The protocol was applied 3 days a week for 14 weeks.

AUTHOR	YEAR	CONSORT	AIM	FOLLOW-UP	COLEMAN SCORE	RESULT	REHABILITATION
				PHYSIOTHERAPY			
HAYES ***[117]***	2004	NO	To compare individualized supervised physiotherapy treatment to a standardized unsupervised home exercise regime. All subjects received a standardized home exercise regime. Subjects who were randomized to the physiotherapy group received additional individualized treatment.	6 months	74	On the basis of these results, outcomes for subjects allocated to individualized physiotherapy treatment after rotator cuff repair are no better than for subjects allocated to a standardized home exercise regime.	For the first post-operative day, the affected extremity was immobilized in a sling. During the 1 postoperative week the patients do some exercise three times per day in accordance with phase one of a three- phase (six month) standardized home exercise regime. Phase two exercises were issued by the treating surgeon at the time of the first post-operative clinic, eight days post-operation Phase three exercises were issued by the treating surgeon at the time of the second post-operative clinic, six weeks post-operative.
JENSSEN ***[118]***	2018	YES	To compare clinical and radiologic results among patients with 3 versus 6 weeks of immobilization after arthroscopic RC repair. Group A was immobilized in a simple sling for 3 weeks, and group B had a brace with a small abduction pillow with the arm in neutral position for 6 weeks	12 months	82	RC repair resulted in improved post- operative shoulder function, regardless of whether the shoulder was immobilized for 3 or 6 weeks. Three weeks of postoperative immobilization with sling use was non-inferior to the commonly used regimen involving 6 weeks of immobilization in a brace with regard to the WORC index at 12 months' follow-up. MRI indicated similar degrees of healing between the groups. Based on these findings, it is safe to immobilize patients in a simple sling for 3 to 6 weeks after repair of small to medium RC tears.	patients were randomized into 2 groups: group A had early active range of motion (ROM) starting at 3 weeks and group B had delayed active ROM starting 6 weeks after surgery Group A had a simple sling for 3 weeks while group B had a brace with a small abduction pillow with the arm in neutral position for 6 weeks after surgery. he patients were told to keep the sling/brace on day and night and to take it off 3 times a day to perform pendulum exercises.<br>All patients were allowed active ROM of elbow and hand and passive ROM of the shoulder joint from day<br>.

AUTHOR	YEAR	CONSO RT	AIM	FOLLOW-UP	COLEMAN SCORE	RESULT	REHABILITATION
				PHYSIOTHERAPY			
KEENER ***[119]***	2014	YES	To compare clinical results and tendon healing rates following arthroscopic RC repair utilizing two distinct rehabilitation protocols. Patients underwent arthroscopic repair of a full- thickness rotator cuff tear measuring <30 mm in width. Postoperatively, patients were randomized either to a traditional rehabilitation program with early range of motion or to an immobilization group with delayed range of motion for six weeks.	24 months	83	Arthroscopic repair of small and medium full-thickness rotator cuff tears results in reliable improvements in clinical outcomes and a high rate of tendon integrity using a double-row repair technique in patients under the age of sixty-five years. There is no apparent advantage or disadvantage of early passive range of motion compared with immobilization with regard to healing or functional outcome.	Subjects in both rehabilitation groups were instructed to wear a standard sling or the initial six weeks after surgery The sling was also removed periodically to allow elbow motion. Physical therapists were given standardized prescriptions outlining the recommended exercises and restrictions pertinent to each phase of rehabilitation
KIM ***[120]***	2012	NO	To test the hypothesis that extracorporeal shock wave therapy (ESWT) stimulates RC healing after arthroscopic repair. The patients were randomized into two groups: patients who underwent ESWT at 6 weeks after surgery (ESWT group) and patients who did not (control group).	12 months	70	This study failed to prove that ESWT stimulates rotator cuff healing after arthroscopic rotator cuff repair. Additional ESWT after rotator cuff repair could theoretically be advantageous, and it was proven to be safe in this study.	This study failed to prove that ESWT stimulates rotator cuff healing after arthroscopic rotator cuff repair. Additional ESWT after rotator cuff repair could theoretically be advantageous, and it was proven to be safe in this study.
KIM ***[121]***	2012	YES	To elucidate whether early passive motion exercise affects functional outcome and tendon healing after arthroscopic RC repair. Patients were randomly allocated into group 1:	12 months	68	Early passive motion exercise after arthroscopic cuff repair did not guarantee early gain of ROM or pain relief but also did not negatively affect cuff healing. We suggest that early passive motion	For group 1, controlled early passive motion exercise consisting of forward flexion, abduction, and external rotation was conducted from 1 day after the operation during the brace-wearing period For group 2, no passive ROM was allowed until brace removal.

AUTHOR	YEAR	CONSORT	AIM	FOLLOW-UP	COLEMAN SCORE	RESULT	REHABILITATION
				PHYSIOTHERAPY			
			early passive motion exercises were conducted 3 to 4 times per day during the abduction brace-wearing period. And group 2: no passive motion was allowed during the same period.			exercise is not mandatory after arthroscopic repair of small to medium-sized full-thickness rotator cuff tears, and postoperative rehabilitation can be modified to ensure patient compliance.	Immobilization was maintained with the abduction brace at 30°. Shrugging of shoulders, active elbow flexion/extension, active forearm supination/pronation, and active hand and wrist motion were encouraged immediately after surgery for both groups. Active-assisted shoulder exercise was encouraged after the weaning of the brace. Muscle strengthening was usually initiated at 9 to 12 weeks postoperatively
KOH KH ***[122]***	2014	YES	To assess the effect of immobilization following RC repair and to determine whether there was any difference in clinical outcome related to the duration of immobilization. Patients were allocated in two groups. Four week s groups: immobilization without passive exercise for 4 weeks. Eight week s groups: immobilization without passive exercise for 8 weeks.	2 months	77	Eight weeks of immobilization did not yield a higher rate of healing of medium-sized rotator cuff tears compared with four weeks of immobilization.	. Stage 1 was an immobilization period in which no passive or active range-of-motion exercise was permitted regardless of the duration of immobilization. A sling with an abduction pillow was applied during stage 1. Normal daily activities were allowed as possible with the arm in the sling. Stage 2 was initiated with the removal of the sling and pillow after four or eight weeks of immobilization. Gentle passive range-of-motion exercise was begun Active or active-assisted motion was allowed as patients obtained a nearly full passive range of motion and confidence with the exercise. the strengthening period was begun at eleven weeks
KRAEUTLER ***[123]***	2015	NO	To compare the effect of compressive cryotherapy (CC) versus ice (IW) on postoperative pain in patients undergoing shoulder arthroscopy for RC repair or subacromial decompression. patients were	7 days	47	There does not appear to be a significant benefit to use of CC over standard IW in patients undergoing shoulder arthroscopy for rotator cuff repair or subacromial decompression. Further study is needed to determine if CC devices are a cost-effective option for postoperative pain	_

AUTHOR	YEAR	CONSORT	AIM	FOLLOW-UP	COLEMAN SCORE	RESULT	REHABILITATION
			PHYSIOTHERAPY				
			randomized to use either CC or a standard IW for the first post-operative week.			management in this population of patients.	
LAM ***[124]***	2015	NO	To study if mechanical stimulation applied postoperatively will enhance tendon-to-bone healing and reduce post- operative re-tear rates. Recruited patients were randomized into 2 groups: one group received a vibration device that oscillated at 80 Hz, and the other group received a placebo device.	24 weeks	83	High-frequency, low-magnitude vibration did provide acute pain relief on application 6 weeks after arthroscopic rotator cuff repair surgery. However, vibration did not improve tendon-to-bone healing, shoulder range of motion, shoulder strength, or shoulder pain with activities, at rest, and at night when compared with placebo.	All patients received the same postoperative rehabilitation protocol for 6 months
LASTAYO ***[125]***	1998	NO	To compare the results of continuous passive motion with those of manual passive range-of-motion exercises after repair of the RC. Two types of postoperative management: continuous passive motion or manual passive range-of-motion exercises	22 months	51	According to the Shoulder Pain and Disability Index, a valid and reliable self-administered questionnaire, the treatment was extremely successful in both groups.	Manual passive range-of-motion exercises were more cost-effective than continuous passive motion. The limited number of physical-therapy visits associated with the manual passive range-of-motion exercises in the present study appeared to be more cost-effective than a traditional physical-therapy schedule of three visits per week. Postoperative therapy with continuous passive motion or manual passive range-of-motion exercises appears to yield favorable results after repair of a small, medium, or large tear of the rotator cuff.
LEE BG ***[126]***	2012	NO	To compare range of motion and healing rates between 2 different rehabilitation protocols after	12 months	79	Pain, range of motion, muscle strength, and function all significantly improved after arthroscopic rotator cuff repair,	Aggressive early passive rehabilitation (manual therapy [2 times per day] and unlimited self–passive stretching exercise) was performed in 30 shoulders (group A) and limited early passive

AUTHOR	YEAR	CONSORT	AIM	FOLLOW-UP	COLEMAN SCORE	RESULT	REHABILITATION
			PHYSIOTHERAPY				
			arthroscopic single row repair for full thickness RC tear. Aggressive early passive rehabilitation (manual therapy [2 times per day] and unlimited self–passive stretching exercise) was performed in Group A and limited early passive rehabilitation (limited continuous passive motion exercise and limited self–passive exercise) Group B.			regardless of early postoperative rehabilitation protocols. However, aggressive early motion may increase the possibility of anatomic failure at the repaired cuff. A gentle rehabilitation protocol with limits in range of motion and exercise times after arthroscopic rotator cuff repair would be better for tendon healing without taking any substantial risks.	rehabilitation (limited continuous passive motion exercise and limited self–passive exercise) in 34 shoulders (group B).
MAHURE ***[127]***	2017	NO	To compare transcutaneous electrical nerve stimulation (TENS) to placebo after RC repair	1 week	73	Results from this prospective double-blinded randomized trial demonstrate that compared with placebo TENS, active TENS can result in significantly less pain and reduced opioid use in the immediate postoperative period after ARCR, suggesting that TENS may be potentially useful in a multimodal approach to managing postoperative pain.	_

AUTHOR	YEAR	CONSORT	AIM	FOLLOW-UP	COLEMAN SCORE	RESULT	REHABILITATION
				PHYSIOTHERAPY			
*MAZZOCCA **[128]***	2017	YES	To compare the effect of early versus delayed motion protocols on quality of life, clinical outcomes in patients who have undergone arthroscopic single-tendon RC repair. The patient were divided into two groups: early motion protocol (starting 2 to 3 days after surgery) or a delayed motion protocol (starting 28 days after surgery).	6 months	82	There was no difference between the delayed and early motion groups in WORC scores at 6 months after surgery. Early motion was associated with lower WORC scores throughout the postoperative period; however, both groups had a similar trajectory of improvement, suggesting both protocols have the same effect on patient-reported improvement. Although failure rates were similar between the groups, the sample size was not sufficient to support a statement regarding the relation between tear morphology and the rehabilitation protocol	Study participants in both groups were immobilized in an Ultrasling with abduction pillow (Corflex, Manchester, NH) to diminish passive tension on the repair. Sling use was discontinued in both groups at the 6-week mark. In addition, study participants in both groups received a postoperative home exercise program consisting of postural exercises and elbow, forearm, wrist, and hand ROM exercises
*OSBAHR **[129]***	2002	NO	To determine the effect of continuous cryotherapy on glenohumeral joint and subacromial space temperatures in the postoperative shoulder. The patient were divided into cryotherapy group and controls	23 hours	58	Our results affirm that reductions in glenohumeral joint and subacromial space temperatures in the postoperative shoulder do occur, leading to potential benefits of continuous cryotherapy as an effective mode of pain control in the postoperative care of patients.	_
*PIITULAINEN **[130]***	2014	YES	To compare a 12-months home-based exercise program with usual care for disability and health-related quality of life after RC repair. The usual care group (UCG) received ordinary postoperative instructions, while the	14 months	65	The home exercise program and usual care were equally effective in improving disability and quality of life after rotator cuff repair. The extra time involved in teaching the home exercise program is not warranted.	The upper arm was maintained beside the body in a suspension bandage for three weeks, Patients were advised to perform postoperative home exercises three times a day. The exercises were started on the first postoperative day. The exercises were started on the first postoperative day. At six weeks, each patient visited the

AUTHOR	YEAR	CONSORT	AIM	FOLLOW-UP	COLEMAN SCORE	RESULT	REHABILITATION
			PHYSIOTHERAPY				
			experimental group (EG) receives advice and instructions on a shoulder muscle strengthening program to be undertaken at home.				outpatient clinic again and was instructed to start dynamic range of motion exercises and strength exercises with a light resistance using yellow resistance bands<br>At six weeks, each patient visited the outpatient clinic again and was instructed to start dynamic range of motion exercises and strength exercises with a light resistance using yellow resistance bands<br>The new experimental intervention was started after two postoperative months, and was based on muscle strengthening exercises performed individually at home<br>The new experimental intervention was started after two postoperative months, and was based on muscle strengthening exercises performed individually at home
RAAB ***[131]***	1996	NO	To determine the effect of continuous passive motion (CPM) on RC repair (RCR). Patient were divided into control group that underwent postoperative physical therapy (PT) and study group (PT plus CPM)	3 months	75	CPM has no effect on overall shoulder score 3 month follow up. CPM has a beneficial effect oh ROM for all patients, as well as on pain relief in female patients and patients > 60 years of age	Patients in the control group underwent a standard postoperative physical therapy control. The study group underwent a similar postoperative physical therapy program but with the addition of shoulder CPM commencing in the recovery room and continuing for 3 week.
RODDEY ***[132]***	2002	NO	To compare the effectiveness of 2 types of home program instruction, videotape versus personal instruction by a physical therapist (PT), on subjective outcomes and exercise compliance following arthroscopic repair of a full-thickness RC tear	52 weeks	74	With a therapist available for questions, patients who utilized the videotape method for their home program instruction had self-reported outcomes equal to patients instructed in their home program personally by a physical therapist. Self-reported compliance with the rehabilitation program had little effect on the outcomes.	Group 1 subjects received exercise instruction solely through a videotape. Subjects in group 2 received 4 separate one-on-one instruction sessions with a physical therapist during the course of the study.

AUTHOR	YEAR	CONSORT	AIM	FOLLOW-UP	COLEMAN SCORE	RESULT	REHABILITATION
				PHYSIOTHERAPY			
SHEPS ***[133]***	2019	NO	To compare the effect of early mobilization (EM) to standard rehabilitation (SR)	24 months	88		
SHEPS ***[134]***	2015	NO	To study the early mobilization following mini-open RC repair. The patients were randomized to either early mobilization or standard rehabilitation groups.	24 months	79	At 24 months post-operatively, patients who performed pain-free, early active mobilization for activities of daily living showed no difference in clinical outcomes from patients immobilized for six weeks following MORCR. We suggest that the choice of rehabilitation regime following MORCR may be left to the discretion of the patient and the treating surgeon.	both groups commenced passive and self-assisted exercises, taught by the hospital PT, on the first post-operative day, the SR patients were told to wear the sling at all times except when performing the passive and self-assisted activities. The EM patients per- formed the same passive and self-assisted activities, but were told that the sling was only needed for comfort and could be taken off and discarded at the patient's discretion. At six weeks, all patients in both groups converged into an identical rehabilitation protocol. Post-operatively, ROM and pain were re-assessed at six weeks and three months
GAROFALO **[135]**	2010	NO	To compare passive self-assisted range of motion exercise versus passive self-assisted range of motion exercise associated with use of continuous passive motion	12 months	72	Postoperative treatment of an arthroscopic rotator cuff repair with passive self-assisted exercises provides a significant advantage in terms of ROM improvement and pain relief when compared to passive self-assisted exercise alone, at the short-term follow-up. No significant differences between the two groups were observed at 1 year postoperatively.	Postoperatively, the shoulder was immobilized in an Ultra Sling II brace for 4 weeks. Group A underwent a protocol of passive self-assisted mobilization supervised by the physiotherapist consisting in 3 series of 10 repetitions each, of pendulum movements and progressive passive abduction, forward flexions and external rotation. Group B underwent an additional assisted passive mobilization protocol using the Arthromot S3 device. for a total of 2 h/day in 4 sessions lasting 30 min each. From the 13th to the 28th week, we continue the passive program to resume complete ROM, and we start active-assisted ROM exercises

AUTHOR	YEAR	CONSORT	AIM	FOLLOW-UP	COLEMAN SCORE	RESULT	REHABILITATION
			PHYSIOTHERAPY				
KLINTBERG **[136]**	2009	NO	T o describe the clinical changes following two different physiotherapy treatment protocols after rotator cuff repair In the traditional group the rotator cuff was protected from loading. Patients were immobilized for six weeks and started with passive range of motion the day after surgery	24 months	60	The present study showed that the progressive protocol produced no adverse effects compared with the traditional protocol.	The progressive group (n = 7) started with dynamic, specific muscle activation of the rotator cuff the day after surgery as well as passive range of motion. After four weeks of immobilization the loading to the rotator cuff increased and in a progressive manner throughout the rehabilitation. In the traditional group (n = 7) the rotator cuff was protected from loading. Patients were immobilized for six weeks and started with passive range of motion the day after surgery. No specific exercises to the rotator cuff were introduced during this period.
RASCHHOFER **[137]**	2017	YES	All participants were randomly assigned to one of the two outpatient treatment groups: primary passive motion versus early isometric loading of the rotator cuff.	12 months	71	This pilot study with early isometric loading of the rotator cuff shows better function and less maximal pain.	All participants were randomly assigned to one of the two outpatient treatment groups: primary passive motion versus early isometric loading of the rotator cuff. Both groups were treated for 12 weeks and performed additionally a home exercise program.

Table S8 Tenotomy and tenodesis

AUTHOR	YEAR	CONSORT	AIM	FOLLOW-UP	COLEMAN SCORE	RESULT	REHABILITATION
				TENOTOMY AND TENODESIS			
DE CARLI ***[138]***	2012	NO	To determine clinical, functional, and radiological results of two groups of patients affected by RC tear with concomitant degeneration of the long head of the biceps tendon treated with tenotomy/tenodesis or tenotomy	23 months	61	Long head of the biceps tenotomy combined with tenodesis does not provide any significant clinical or functional improvement than isolated tenotomy. However, the incidence of the Popeye sign is significantly higher, even though not associated with any functional dysfunction.	_
LEE HJ ***[139]***	2016	YES	To compare the outcomes of tenotomy to outcomes of tenodesis for treatment of long head of biceps tendon (LHBT) lesions with RC tears. Arthroscopic LHBT tenotomy was done in (group I), and LHBT tenodesis was done for (group II) with rotator cuff repair	12 months	88	For the treatment of LHBT lesions with rotator cuff tear, patients with tenotomy and tenodesis both showed significant improvements in functional scores. The incidence of Popeye deformity was about 3-times higher in tenotomy group. No significant differences in elbow motor power were observed except greater forearm supination power in the tenodesis group.	The patients were fitted with an abduction brace immediately after the operation. The abduction brace was worn for 4 weeks postoperatively, and the same standardized rehabilitation protocols were prescribed in both groups. The patients were asked to undergo home-based active assisted shoulder exercises 3 times daily, TheraBand exercises, strengthening exercises for the muscles
OH JH ***[140]***	2016	NO	To compare clinical outcomes in patients with concomitant superior labrum – biceps complex (SLBC) lesions and RC tears who underwent arthroscopic rotator cuff repair, according to 3 different treatment methods (simple	12 months	69	All 3 treatments improved pain and function. Simple debridement showed the lowest risk of the Popeye deformity and preserved forearm supination strength. Biceps tenotomy and tenodesis may be preferable for selected patients: biceps tenotomy for patients with definite	All patients followed the planned rehabilitation protocol. Immobilization was maintained with an abduction brace, and the duration of immobilization was based on the tear size measured at the time of operation: 4 weeks for small tears (<1 cm), 5 weeks for medium tears (1 to 3 cm), and 6 weeks for large to massive tears (>3 cm). Shrugging of both shoulders, active elbow flexion/extension, active forearm supination/pronation, and active hand and

AUTHOR	YEAR	CONSORT	AIM	FOLLOW-UP	COLEMAN SCORE	RESULT	REHABILITATION
			TENOTOMY AND TENODESIS				
			debridement, biceps tenotomy, or biceps tenodesis) for the SLBC lesions			bicipital groove tenderness and biceps tenodesis for patients, especially male patients, with bicipital groove tenderness who want to preserve supination strength.	wrist motion were encouraged immediately after surgery, and the surgical wounds in all patients were checked at 2 weeks postoperatively. Active-assisted shoulder ROM exercises began after weaning from the brace, and muscle strengthening was started at 9 to 12 weeks postoperatively with respect to tear size.
*ZHANG **[141]***	2015	NO	To compare the clinical outcomes of the two procedures in patients older than 55 years of age affected by reparable RC tears with concomitant long head biceps pathologies. Patients were randomly assigned to the tenotomy group or the tenodesis group.	25 months	83	Both tenotomy and tenodesis are effective and equal for the treatment of long head biceps lesions. However, because tenotomy requires a shorter surgical time and results in faster pain relief, tenotomy may be more suitable for the treatment of long head biceps lesions in patients older than 55 years of age with reparable rotator cuff tears.	All the patients (tenotomy or tenodesis) followed the routine rehabilitation procedures after rotator cuff repair. There was no additional procedure for tenotomy, except immobilization of their elbow motion for 1 week. How- ever, the patients treated with tenodesis progressed to passive range of motion of the elbow 1 week post operation and active range of motion and gentle strength training 6 weeks post operation. Unrestricted use of the biceps muscle was not allowed until 16–20 weeks post operation.
*BELAY **[142]***	2019	NO	To evaluate differences in pain relief and subjective outcomes between biceps tenotomy versus tenodesis for long head biceps tendinopathy. Patients were randomized and blinded to biceps tenotomy versus arthroscopic tenodesis intra-operatively.	24 months	72	Outcomes appear similar between biceps tenotomy versus tenodesis; however, the tenotomy group demonstrated greater incidence of cosmetic deformity but an earlier improvement in postoperative pain.	_
*HUFELAND **[143]***	2019	NO	To evaluate elbow flexion and forearm supination force as well as the biceps muscle distalization in patients with isolated LHB lesions treated with	12 months	66	This prospective randomized study comparing LHB tenodesis and tenotomy in isolated LHB lesions has shown no significant difference in elbow flexion and	Early functional physiotherapy with active and passive free glenohumeral motion without immobilization was allowed from the first day postoperatively. Elbow flexion was limited to less than 1 kg for 12 weeks postoperatively

AUTHOR	YEAR	CONSORT	AIM	FOLLOW-UP	COLEMAN SCORE	RESULT	REHABILITATION
TENOTOMY AND TENODESIS							
			tenodesis or tenotomy			forearm supination force and clinical scores after 12 months. After LHB tenotomy, there was a non-significant trend for a higher rate of popeye sign deformities of the upper arm and biceps muscle cramps.	

Table S9 Mini open and arthroscopic repair

AUTHOR	YEAR	CONSORT	AIM	FOLLOW-UP	COLEMAN SCORE	RESULT	RHEABILITATION
			MINI OPEN AND ARTHROSCOPIC REPAIR				
CARR ***[144]***	2017	NO	To compare the effectiveness of arthroscopic (Group 1) to open repair (Group 2) of the RC. 136 patients underwent arthroscopic surgery and 137 patients to open surgery	24 months	73	There is no evidence of difference in effectiveness between open and arthroscopic repair of rotator cuff tears. The rate of re-tear is high in both groups, for all sizes of tear and ages and this adversely affects the outcome.	_
CHO CH ***[145]***	2012	NO	To compare arthroscopic (Group1) and mini-open (Group 2) repair for RC tears.	6 months	69	Arthroscopic and mini-open repair had equivalent clinical outcome in the early post-operative period. The hypothesis that arthroscopic repair would cases less postoperative period compared with mini-open repair was not supported	The postoperative rehabilitation protocol was the same for both groups . Wearing an abduction brace, patients engaged in pendulum and continuous passive motion machine exercises were started at 6 week postoperatively, muscle-strengthening exercise were started at 3 month, and occupational or sport activities were started at 6 month
LIU ***[146]***	2017	NO	To compare the clinical outcomes of patients undergoing all-arthroscopic (Group 1) or mini-open (Group 2) RC repair.	16.6 months	77	The AA approach was associated with less pain and lower DASH score as well as higher CMS score in the early recovery period. No difference was found between the 2 groups in primary and secondary outcomes in the long term, or incidence of complications such as adhesive capsulitis and rotator cuff retear. In conclusion, we consider that the AA procedure has better recovery at short-term follow-ups, while both techniques are equivalent regarding long-term outcomes.	The postoperative rehabilitation protocol was the same for both groups. Wearing an abduction brace, patients engaged in pendulum and continuous passive motion machine exercises until postoperative day 5, and then passive range-of-motion exercises were started. Active range-of-motion exercises were started at 6 weeks postoperatively, muscle-strengthening exercises were started at 3 months, and occupational or sports activities were started at 6 months.

AUTHOR	YEAR	CONSORT	AIM	FOLLOW-UP	COLEMAN SCORE	RESULT	RHEABILITATION
			MINI OPEN AND ARTHROSCOPIC REPAIR				
VAN DER ZWAAL ***[147]***	2013	NO	To compare clinical outcomes in the first postoperative year of patients with full-thickness small to medium-sized tears undergoing all-arthroscopic (AA) versus mini-open (MO) RC repair	52 weeks.	83	Functional outcome, pain, range of motion, and complications do not significantly differ between patients treated with all-arthroscopic repair and those treated with mini-open repair in the first year after surgery. Patients do attain the benefits of treatment somewhat sooner (6 weeks) with the arthroscopic procedure.	Active exercises of the elbow, wrist, and hand were encouraged immediately. The rehabilitation protocol consisted of active abduction in the scapular plane limited to 70 and 0 of external rotation in the first 4 to 6 weeks as tolerated. After this, active range of motion exercises were started. When the patient was free of pain, scapula and rotator cuff isotonic strengthening exercises were initiated.

Table S10 Subacromial decompression

AUTHOR	YEAR	CONSORT	AIM	FOLLOW-UP	COLEMAN SCORE	RESULT	RHEABILITATION
			SUBACROMIAL DECOMPRESSION				
BIDWAI ***[148]***	2016	NO	To assess the efficacy of arthroscopic subacromial decompression (Group ASAD) versus mini-open repair (Cuff repair group) for the treatment of medium sized RC tears in two different groups of patients	7 years	85	In this medium- to longer-term study, there is no demonstrable significant benefit of cuff repair over decompression alone for the treatment of medium-sized rotator cuff tears, in terms of ASES, DASH and Constant scores for pain, function and strength modules. The presence of cuff tear does not necessitate surgical repair. This conclusion should drive surgical strategies and shared decision-making between patients and surgeons.	_
FLURIN ***[148]***	2013	NO	To evaluate if arthroscopic repair (Group 1) would be better than decompression (Group 2) in older patients who are likely to be less active and may have more severely degenerated RC tendons.	12 months	76	Therewasasignificantimprovementinall-clinicalscoresforbothtechniques1year after surgery. Repair was significantly better than decompression for all clinical outcomes, even in patients above 75 years of age. The difference observed between repair and decompression was greater in patients with more retracted tears and lesser in patients with more severe fatty infiltration.	For all patients, a standardized postoperative protocol was implemented with early self-rehabilitation and splint immobilization for 8—10 days after pain had disappeared in patients who had received decompression or for 6 weeks in patients who under went rotator cuff repair.
GARTSMAN ***[149]***	2004	NO	To determine whether arthroscopic subacromial decompression changes the outcome of RC repair. Group1 received arthroscopic subacromial	12 months	56	within the parameters described above, arthroscopic subacromial decompression does not appear to change the functional outcome after arthroscopic repair of the rotator cuff.	_

AUTHOR	YEAR	CONSORT	AIM	FOLLOW-UP	COLEMAN SCORE	RESULT	RHEABILITATION
			SUBACROMIAL DECOMPRESSION				
			decompression. Group 2 doesn't received arthroscopic subacromial				
MILANO ***[150]***	2007	NO	To evaluate the role of subacromial decompression in the arthroscopic repair of full-thickness RC tears. Group 1 received arthroscopic rotator cuff repair with subacromial decompression. In group 2 the repair was performed without decompression.	24 mounts	68	At short-term follow-up, subacromial decompression did not seem to significantly affect the outcome of arthroscopic rotator cuff repair. Longer follow-up studies will be necessary to confirm the clinical relevance of these observations.	all patients underwent the following rehabilitation program. First phase range of motion exercise program, Second phase muscle strengthening program and third phase open kinetic chain exercise.
NAM ***[151]***	2018	NO	To evaluate the effects of extensive bursectomy (EB) and limited bursectomy (LB) during arthroscopic RC repair. In the EB group subacromial bursa were thoroughly removed from anterior to posterior and lateral to medial. In the LB group, bursectomy was minimized to allow torn cuff visualization and tendon repair	12 months	81	EB during arthroscopic rotator cuff repair appears to have no benefit in terms of reducing pain. More adhesions in the subacromial space after EB may result in slower motion recovery, especially in terms of ER. The extent of bursectomy did not affect tendon integrity. However, marked bursal thickening was more frequently observed in the EB group.	The same rehabilitation protocol was used in the 2 groups. Immobilization was maintained with a sling and abduction pad for 5 weeks in all patients. After weaning from brace use, patients were instructed on how to conduct passive assisted stretching exercises and active exercises (forward elevation, external rotation, and internal rotation before active exercises). Return to sports was not allowed until approximately 5 months after surgery.

Table S11 Surgical tecniques

AUTHOR	YEAR	CONSORT	AIM	FOLLOW-UP	COLEMAN SCORE	RESULT	RHEABILITATION
			**SURGICAL TECNIQUES**				
BOEHM ***[152]***	2005	NO	To compare the clinical results of two suture techniques for which different suture materials were used. Group 1 had transosseous repair with No. 3 Ethibond using modified Mason-Allen sutures and group 2 had transosseous repair with 1.0 mm polydioxanone cord using modified Kessler sutures.	30 months	75	No significant statistical difference was seen between the two groups: Constant score, 91% vs 92%; rate of further tear, 18% vs 22%; and revision, 4% vs 4%. In cases of further tear the outcome in group 2 did not differ from that for the intact repairs	-
RANDELLI ***[153]***	2015	NO	To evaluate whether treating partial-thickness articular-sided tears of the upper subscapularis (ssC) tendon with a dedicated suture anchor would result in an internal rotation strength improvement compared with simple shaving of the ssC tendon and footprint	42 months	65	Partial-thickness articular-sided tear of the upper ssC tendon in association with a posterosuperior rotator cuff repair and LHB treatment, when limited to the superior centimeter of the ssC tendon, shows a comparable performance in terms of strength in internal rotation either after simple shaving or a tendon-to-bone repair.	The postoperative protocol was: four weeks wearing a sling followed by four weeks of passive stretching, and then active strengthening. During the first four weeks patients were allowed
BIGONI ***[154]***	2009	NO	To evaluate, with the isokinetic testing, the recovery of strength in patients with RC tears treated with two different arthroscopic repair techniques. Patients with a full-thickness supra-spinatus tear were randomized to two different groups. Patients in group 1 underwent side-to-side repair with permanent sutures, whereas those	12 months	58	We showed a strength difference between patients with side-to-side repairs and those with tendon-to-bone repairs.	The postoperative program was the same for both groups: neutral rotation in a sling with a 15° abduction pillow for 4 weeks, a pool therapy program for passive motion in the third week, and active assisted exercises in the sixth week with the goal of complete range-of-motion recovery, followed by isometric, isotonic, and isokinetic training to encourage strength recovery.

AUTHOR	YEAR	CONSORT	AIM	FOLLOW-UP	COLEMAN SCORE	RESULT	RHEABILITATION
			**SURGICAL TECNIQUES**				
			in group 2 underwent tendon-to-bone fixation with 1 metal suture anchor loaded with double sutures.				
FRANCESCHI ***[155]***	2013	YES	To compare two groups of patients who underwent two different arthroscopic procedures for repair of articular sided partial-thickness RC tears Group1 underwent arthroscopic rotator cuff repair with a transtendon technique; Group 2 underwent arthroscopic full-thickness conversion and repair of the lesion.	36 mounts	73	The two procedures are safe, effective, and comparable.	Patients wore a sling at 0° of external rotation and 15° of abduction for four weeks, and were immediately allowed active and passive flexion of the elbow, 0° passive external rotation with the arm at the side, capsular stretching exercises, and scapulothoracic motion At two weeks, patients were reviewed for removal of stitches and dressings, and were recommended to start gentle active motion exercises supervised by an expert physiotherapist. Patients started water and elastic band exercises at four weeks, free ROM in all directions at six weeks, and resisted strengthening exercises at 12 weeks.
KO ***[156]***	2008	NO	To compare the clinical results and failure rates of arthroscopic RC repair by use of a modified mattress locking stitch (MMLS) repair versus a simple stitch repair. Thirty-nine individuals underwent arthroscopic repair by use of an MMLS (group I). Thirty-nine individuals underwent arthroscopic repair by use of a simple stitch (group II).	31.1 mounts	78	Arthroscopic repair of medium-sized (1.5- to 3-cm) full-thickness rotator cuff tears by use of an MMLS improves patient satisfaction rates and radiographic repair integrity in comparison to simple stitch repair.	_
MILANO ***[157]***	2010	NO	To compare the clinical outcome of arthroscopic RC repair with metal and biodegradable suture anchors. Metal anchors were used in	24.4 +/- 2.6 mounts	85	Arthroscopic repair of medium-sized (1.5- to 3-cm) full-thickness rotator cuff tears by use of an MMLS improves patient satisfaction rates and radiographic repair integrity in	Patients underwent the following rehabilitation program adapted from principles of shoulder re- habilitation of Kibler. First phase (4-8 weeks after surgery) range of moton exercise program. Second phase(9-12 weeks after surgery)

AUTHOR	YEAR	CONSORT	AIM	FOLLOW-UP	COLEMAN SCORE	RESULT	RHEABILITATION
**SURGICAL TECNIQUES**							
			group 1 and biodegradable anchors in group 2.			comparison to simple stitch repair.	muscle strengthening program. Third phase (13-16 after surgery) open kinetic chain exercises and postural rehabilitation
SHIN ***[158]***	2012	NO	To compare the clinical outcomes of patients who underwent partial-thickness articular-sided RC repairs by 2 surgical techniques. 24 patients received arthroscopic rotator cuff repair with a transtendon technique (group 1) and 24 patients received arthroscopic rotator cuff repairs after tear completion (group 2I)	31 months	77	Arthroscopic repair of partial-thickness articular-sided rotator cuff tears exceeding 50% of the tendon thickness provided satisfactory functional improvements and pain relief regardless of the repair technique. Although repair after conversion to a full-thickness tear showed less postoperative morbidity, tendon integrity is of primary concern after repair. On the other hand, the transtendon repair technique resulted in complete tendon integrity but slower functional recovery.	_
MOHSEN MARDANI-KIVI **[159]**	2019	YES	To compare clinical and functional outcomes of open sub- pectoral versus arthroscopic intraarticular tenodesis. In the IA group, an anchor suture was used for both rotator cuff repair and LHBT tenodesis. In the SP group, after arthroscopic repair of the rotator cuff, subpectoral tenodesis of LHBT was performed using an interference screw	24 months	82	The two groups were similar with regard to demographic characteristics and preoperative evaluations (all $P > 0.05$). The functional status of both groups was improved, but not significantly differently so between the two groups ($P = 0.1$ and $P = 0.4$, respectively). Pain intensity decreased during the 2-year follow-up period, similarly so in the two groups. Patient satisfaction was also similar in the two groups	-
RANDELLI ***[160]***	2017	YES	To evaluate clinical and radiological results of arthroscopic RC repair using 2 different techniques: single-row anchor fixation versus transosseous hardware-	12 months	79	No significant differences were found between the 2 arthroscopic repair techniques in terms of functional and radio-logical results. However, postoperative pain decreased more quickly after the	All patients enrolled wore an arm-sling day and night for 4 weeks after surgery; during that period the sling was removed only to eat and perform personal hygiene and light exercises of mobilization of the elbow and scapulothoracic joint. From the 29th day, unless otherwise indicated, patients began

AUTHOR	YEAR	CONSORT	AIM	FOLLOW-UP	COLEMAN SCORE	RESULT	RHEABILITATION
			**SURGICAL TECNIQUES**				
			free suture repair			transosseous procedure, which therefore emerges as a possible improvement in the surgical repair of the rotator cuff.	passive physical therapy to recover the full range of motion of the shoulder joint. From the end of the second month, patients started active physical therapy, lasting 4 weeks, to regain muscle strength.
KIM ***[161]***	2019	YES	To investigate preemptive extensive rotator interval (RI) release during arthroscopic rotator cuff repair. The patients were divided into two groups: the preemptive extensive RI release group and the RI non-release group.	26.5 months	84	Arthroscopic preemptive extensive RI release can reduce early postoperative shoulder stiffness after ARCR but does not significantly change the overall clinical outcome after surgery.	For 1 month postoperatively, an abduction brace was applied. After the brace was discarded, pendulum exercise was started. Two months postoperatively, patients started sleeper-stretch and pulley exercises. Three months postoperatively, patients began isometric exercises using a yellow Thera-Band to restore the power of ER and IR. All sports activities were permitted 6 months after the surgery, and the use of the shoulder was not limited within a tolerable loading.

Table S12 Miscellaneous

AUTHOR	YEAR	CONSORT	AIM	FOLLOW-UP	COLEMAN SCORE	RESULT	REHABILITATION
				MISCELLANEOUS			
BERTH ***[162]***	2010	NO	To investigate the effectiveness of arthroscopic debridement or arthroscopic partial repair in patients with massive RC tear	24 +/- 2 months	70	During the follow-up period all patients in our series had good or satisfactory outcome after rotator cuff surgery. Regardless of high rates of structural failure of the partial rotator cuff repair, the results of arthroscopic partial rotator cuff repair demonstrated slightly better functional outcome than debridement.	In group 1, an abduction pillow was worn during the first 4 weeks after surgery. Passive mobilization and assisted active exercises within the pain-free range of motion were also performed up to 6 weeks after surgery.<br>In group2, patients were mobilized rapidly and a sling was worn only if required during the first 10 days postoperatively. Passive and active range-of-motion exercises started the first day after surgery and continued until maximum movement was achieved. All patients were treated with continuous passive motion within the first 3 weeks.
BLANCHARD ***[163]***	1999	NO	To evaluate diagnostic and therapeutic impact of MRI and arthrography in the investigation of full-thickness RC tears	6 months	58	The accuracy of MRI for FTRCT in 38 patients with surgical confirmation was 79%, sensitivity 81% and specificity 78%; the accuracy of arthrography was 82%, sensitivity 50% and specificity 96%.Magnetic resonance imaging may be the preferred investigation because of its better demonstration of soft tissue anatomy.	_
CAPITO ***[164]***	2017	NO	To compare the clinical effects of a hyperosmolar (593 mOsm/L) irrigation solutions to a standard isotonic isotonic (273 mOsm/L) solution for shoulder arthroscopy..	12 months	74	A hyperosmolar irrigation solution provides a safe and effective way to decrease periarticular fluid retention associated with arthroscopic rotator cuff surgery without any adverse long-term effects. Use of a hyperosmolar irrigation solution for shoulder arthroscopy has potential clinical benefits to surgeons and patients.	_

AUTHOR	YEAR	CONSORT	AIM	FOLLOW-UP	COLEMAN SCORE	RESULT	REHABILITATION
			MISCELLANEOUS				
CASTAGNA ***[165]***	2015	NO	To compare the clinical and subjective difference between trans-tendon repair or complete/repair in patients affected by deep partial articular supraspinatus tear. The first group (A) was treated with arthroscopic transtendon repair while the second group (B) was treated with an arthroscopic completion of the tear and formal repair.	24 months	69	Both repairing techniques of deep partial supraspinatus tear provide good results in terms of function and pain. There were no statistically significant differences between the two techniques.	All patients were immobilized in a 20° abduction sling for 4 weeks when they then started a normal rehabilitation protocol for 4 months.
CHIERICHINI ***[166]***	2015	YES	To compare the occurrence rate of hypotensive and bradycardic events (HBEs) during arthroscopic RC repair performed with interscalene brachial plexus block anesthesia in the sitting position in 2 groups of patients who underwent the procedure with norepinephrine or epinephrine added to the irrigation fluid	12 months	72	Continuous administration of norepi- nephrine, 0.66 mg/L, diluted in irrigation fluid during arthroscopic rotator cuff repair with the patient in the beach-chair position reduces the incidence of HBEs and is as effective as epinephrine in controlling intraoperative bleeding and maintaining the visual clarity of the surgical field.	_

AUTHOR	YEAR	CONSORT	AIM	FOLLOW-UP	COLEMAN SCORE	RESULT	REHABILITATION
			MISCELLANEOUS				
FRANCESCHI ***[167]***	2008	YES	To evaluate if there are advantages in repairing a type II SLAP lesion when associated with a RC tear in patients over 50 years of age. In Group1 was repaired the rotator cuff and the type II SLAP lesion . In the Group 2 was repaired the rotator cuff and tenotomized the long head of the biceps.	35 months	78	There are no advantages in repairing a type II SLAP lesion when associated with a rotator cuff tear in patients over 50 years of age. The association of rotator cuff repair and biceps tenotomy provides better clinical outcome compared with repair of the type II SLAP lesion and the rotator cuff.	The arm was supported using a sling with an abduction pillow for 6 weeks. Active elbow flexion and extension were allowed, but terminal extension was restricted. Passive external rotation was started from the first day after surgery, and maintained within a comfortable range. Overhead stretching was restricted until 6 weeks postoperatively to avoid damaging the repair. At 6 weeks, the sling was removed, and overhead stretching with a rope and pulley were started. Rehabilitation was continued for 6 months.
GERVASI ***[168]***	2016	NO	To assess the safety and efficacy of fluoroscopy-guided biodegradable spacer implantation under local anesthesia, in patients with massive rotator cuff tears and comorbidities	24 months	66	Fluoroscopy-guided implantation of InSpace™ system under local anesthesia, represented an effective alternative to the existing procedures. This procedure may be considered as a treatment option for elderly patients or for patients with multiple comorbidities complicating or contraindicating surgery under general anesthesia. Technically easy, this technique can be an effective tool in the armamentarium of most orthopedic surgeons.	–

AUTHOR	YEAR	CONSORT	AIM	FOLLOW-UP	COLEMAN SCORE	RESULT	REHABILITATION
			MISCELLANEOUS				
KIM ***[169]***	2011	NO	To evaluate general shoulder pain and the overall shoulder function after combined RC repair and acromioclavicular joint resection in patients with concomitant asymptomatic acromioclavicular arthritis. Group 1 included 31 patients, who underwent arthroscopic distal clavicle resection combined with rotator cuff repair. Group 2 included 52 patients, who underwent isolated rotator cuff repair.	24 months	71	This study shows that distal clavicle resection combined with rotator cuff repair for asymptomatic ACJ arthritis with inferiorly directed osteophytes lower functional scores due to temporary pain in early postoperative periods, but better functional outcomes with satisfactory pain relief and no reoperation rate were observed after 2 years.	_
MILANO ***[170]***	2013	YES	To evaluate the efficacy of a marrow-stimulating technique with microfractures of the greater tuberosity during arthroscopic RC repair. Group 1 underwent standard repair; in group 2, microfractures of the greater tuberosity were performed to enhance tendon repair	28 .1 +/- 3 months	77	Postoperative magnetic resonance imaging did not show any significant difference between groups in structural integrity. However, subgroup analysis showed a significantly greater healing rate in the microfracture group for large tears involving the supraspinatus and infraspinatus.	After surgery, a sling was applied to the operated limb and was maintained for 4 weeks. After this period, all patients underwent the same rehabilitation program adapted from principles of shoulder rehabilitation described by Kibler et al. No differences in the rehabilitation program were considered according to the treatment group or extent of rotator cuff tear.

AUTHOR	YEAR	CONSORT	AIM	FOLLOW-UP	COLEMAN SCORE	RESULT	REHABILITATION
			MISCELLANEOUS				
OH JH ***[171]***	2014	YES	To evaluate the clinical effectiveness of arthroscopic distal clavicle resection (DCR) in patients with RC tears and concomitant asymptomatic radiological ACJ arthritis. Patients in group 1 (39 patients) underwent additional arthroscopic DCR, while patients in group 2 (39 patients) did not.	24 months	76	Preventive arthroscopic DCR in patients with rotator cuff tears and concomitant asymptomatic radiological ACJ arthritis did not result in better clinical or structural outcomes, and it did lead to symptomatic ACJ instability in some patients. Preventive arthroscopic DCR is not recommended in patients with radiological but asymptomatic ACJ arthritis. Further long- term follow-up is needed to confirm the development of symptoms in ACJ arthritis.	Immobilization was maintained with the abduction brace at 30°. Shoulder shrugging, active elbow flexion/extension, active forearm supination/pronation, and active hand and wrist motion were encouraged immediately after surgery. No passive shoulder motion was allowed during brace-wearing periods (4-6 weeks after surgery). After weaning off the brace, patients were encouraged to begin active-assisted range of motion (ROM) exercises. Muscle strengthening exercises were usually initiated at 9 to 12 weeks postoperatively
OH JH ***[172]***	2014	NO	To compare the clinical efficacy of warmed irrigation fluid (36°) and room-temperature fluid in decreasing perioperative hypothermia during arthroscopic RC surgery. .	1 day	57	Warmed irrigation fluid was not superior to room-temperature irrigation fluid in reducing the occurrence of perioperative hypothermia during arthroscopic shoulder surgery	_

AUTHOR	YEAR	CONSORT	AIM	FOLLOW-UP	COLEMAN SCORE	RESULT	REHABILITATION
			MISCELLANEOUS				
*OSTI **[173]***	2013	NO	To evaluate if microfractures would significantly enhance vascular and healing responses over the RC insertion site and improve clinical and functional outcomes. Group 1 patients underwent microfracture at the footprint in the treatment group. The patients in the control group did not receive that treatment	29 months	69	Microfractures at the footprint are simple, safe, inexpensive and effective at producing less pain in the short term in patients who undergo rotator cuff repair, but at two years they do not result in significantly different outcomes, either clinically or at imaging, compared to traditional rotator cuff repair.	Post-operatively, all patients wore a sling for four weeks; passive ROM exercises were started two weeks after surgery and continued for four weeks. Active exercises were started at six weeks; strengthening exercises were allowed at a minimum of three months.
*PARK **[174]***	2015	YES	To compare the clinical results between patients who had distal clavicle resection (DCR) and those who did not during RC repair (RCR). Patients were allocated in DCR+ RCR group and RC repair only (isolated RCR).	24 months	68	There was no difference in the clinical evaluations between the combined arthroscopic DCR and RCR group and the isolated RCR group at a minimum 24-month follow-up. Arthroscopic DCR should be carefully considered in patients who have symptomatic ACJ arthritis with RC tears.	The shoulder was immobilized with an abduction brace for 4 weeks. During this period, the brace was used except for hygiene purposes and changing clothes. Hand exercises (repeat squeeze and release of a rubber ball) and daily activities below the wrist level were allowed on the affected side. After 4 weeks, the brace was removed and the patients were permitted to perform active-assistive passive range of motion exercises and, eventually, an active strengthening exercise after full range of motion was achieved.

AUTHOR	YEAR	CONSORT	AIM	FOLLOW-UP	COLEMAN SCORE	RESULT	REHABILITATION
			MISCELLANEOUS				
SENEKOVIC ***[175]***	2017	NO	To confirm the long-term safety and efficacy of the biodegradable inflatable In Space TM system in patients with massive reparable or irreparable RC tears	60 months	66	InSpace^{TM} system represented an effective alternative to the existing arthroscopic procedures in patients with painful massive RCT refractory to conservative management. Further randomized controlled trials comparing the clinical and functional outcomes after implantation of the InSpace^{TM} device are warranted.	–
TORRENS ***[176]***	2019	NO	T o evaluate patient treatment decision-making, based on the benefits or on the side effects in rotator cuff disorders. Patients were randomly allocated to either group A (benefit-inform) or group B (side effect-inform).	24 months	75	The way that information on rotator cuff disorders is provided strongly influences patients' treatment decisions.	_
NICHOLSON ***[177]***	2019	NO	To investigate the influence of age on the cost-effectiveness of arthroscopic rotator cuff repair	24 months	77	Arthroscopic rotator cuff repair results in excellent patient satisfaction and cost-effectiveness, regardless of age.	Rehabilitation involved a standardized physiotherapy program starting four weeks postoperatively.

AUTHOR	YEAR	CONSORT	AIM	FOLLOW-UP	COLEMAN SCORE	RESULT	REHABILITATION
			MISCELLANEOUS				
SHIBATA ***[178]***	2001	NO	To evaluate sodium hyaluronate for the treatment of patients with RC tear. 25 mg of sodium hyaluronate was injected into shoulders in one group (1 Group) and 2 mg of dexamethasone was injected in the other group (Group 2)	6 months	67	Therapeutic efficacy in the SH group was equivalent to that in the steroid group. In both groups, the rate of patients who engaged in manual labor was significantly higher in the group of unsatisfied patients than in that of satisfied patients. No adverse reaction to either treatment was observed. These results suggest that SH is an effective conservative treatment for patients with rotator cuff tears.	_

Table S13 Post- operative immobilization

AUTHOR	YEAR	CONSORT	AIM	FOLLOW-UP	COLEMAN SCORE	RESULT	REHABILITATION
						**POST-OPERATIVE IMMOBILIZATION**	
CONTI ***[179]***	2015	NO	To asses clinical outcomes comparing post-operative immobilization with a 15° external rotation brace (ER Group) to an internal rotation sling (IR Group).	12 months	73	Patients operated with isolated superior or posterosuperior rotator cuff tear immobilised with brace in 15° of ER position showed less pain and a better passive range of motion at short time after surgery.	The rehabilitation program for both groups was similar: in the first 5 weeks, they had to wear the sling (in ABD 15° and ER or IR 15° according to the respective group) day and night and were advised to perform at home pendular exercises and supine passive motions exercises to maximum 90° of elevation and 30° of external rotation. No active motion was allowed for 6 weeks. Then, the patients underwent to a similar outpatient rehabilitation protocol supervised by a professional therapist.
HOLLMAN ***[180]***	2017	NO	To compare post-operative immobilization with a abduction brace to an antirational sling	12 months	68	In the short term, the level of pain, function, and quality of life were not significantly different between the use of an abduction brace and that of an anti- rotation sling after arthroscopic rotator cuff repair. Based on these findings, the abduction brace used in this study does not seem to be the solution for decreasing the pain experienced in the first postoperative weeks after arthroscopic rotator cuff repair, and both are recommendable.	Directly after surgery, patients were instructed to start with pendulum exercises. From the first week after surgery, patients started physiotherapy with restricted passive ROM up to 70 of abduction, 70 of forward elevation, and 20 of external rotation in the scapular plane.<br>From 6 weeks, the immobilization was phased out and (guided) active motion exercises were started. From 3 months on, active motion exercises above the shoulder level were allowed.
GHANDOUR ***[181]***	2019	NO	To compare post-operative immobilization with an abduction brace (ABG Group) to a sling (PASG Group).	12 months	75	Our study did not find a significant difference between abduction brace and antirotation sling in patient-oriented outcome measures or postoperative pain after rotator cuff repair.	During the first 6 weeks, a passive ROM program was encouraged. Starting from the seventh week, a postoperative active exercise program was started Before follow-up visits, clinical assessment, or physiotherapy sessions, patients were seen first by the key holder, who removed the abduction brace and applied a pouch arm sling to all patients; later, after the end of the visit, the abduction brace was reapplied to the abduction brace group (ABG).
TIREFORT ***[182]***	2019	YES	To compare clinical and radiographic outcomes after rotator cuff repair with and without postoperative sling immobilization.	6 months	68	No immobilization after rotator cuff repair is associated with better early mobility and functional scores in comparison with sling immobilization. Postoperative immobilization with a sling may therefore not be required for patients treated for a small or medium tendon tear.	In the sling group, the arm was immobilized at the body with a sling for 4 weeks. During their hospital stay, all patients received identical surgeon recommendations to perform self-mobilization of the shoulder 5 times a day. Passive mobilization was performed by both groups during the first 4 postoperative weeks, and this was followed by progressive active mobilization. Patients in the no-sling group were not allowed to perform active abduction- elevation<br>The postoperative rehabilitation protocols were otherwise

AUTHOR	YEAR	CONSORT	AIM	FOLLOW-UP	COLEMAN SCORE	RESULT	REHABILITATION
				**POST-OPERATIVE IMMOBILIZATION**			
							identical for both groups. After 4 weeks, both groups were allowed to perform daily exercises and active motion exercises with the elbow at the side (without strengthening), but no exercises involving lifting of the elbow in any direction, unless assisted. strengthening program was permitted only after 3 postoperative months.

Table S14. The rate of missed checklist items of consort checklist for each trial

Articles	Year	no. missed checklist item	ratio missed checklist item
Aagard	2019	15	0.4
Abrams	2014	13	0.4
Alvarez	2005	13	0.4
Analan	2015	17	0.5
Analay Akbaba	2019	10	0.3
Analay Akbaba	2018	10	0.3
Arias-Buria	2015	14	0.4
Arndt	2012	23	0.6
Avanzi	2019	16	0.4
Banerjee	2008	19	0.5
Bang	2010	17	0.5
Barber	2002	22	0.6
Barber	2012	17	0.5
Baumgarten	2016	14	0.4
Behr	2012	18	0.5
Belay	2019	12	0.3
Berth	2010	20	0.5
Bidwai	2016	17	0.5
Bigoni	2009	20	0.5
Blanchard	1999	22	0.6
Blum	2009	17	0.5
Boehm	2005	13	0.4
Borgeat	2010	17	0.5
Burks	2009	18	0.5
Cai YZ, MED SCI	2018	16	0.4
Cai YZ, AM J	2018	15	0.4
Capito	2017	18	0.5

Carbonel	2012	18	0.5
Carr	2017	11	0.3
Castagna	2015	18	0.5
Castricini	2011	11	0.3
Chierichini	2015	13	0.4
Cho CH	2011	20	0.5
Cho CH	2012	20	0.5
Cho CH	2015	20	0.5
Cho NS	2007	19	0.5
Choi	2018	21	0.6
Chou	2010	14	0.4
Coghlan	2009	8	0.2
Conti	2015	24	0.6
Coory	2019	11	0.3
Cuff	2012	13	0.4
Culebras	2001	19	0.5
D’ambrosi	2016	15	0.4
De Carli	2012	20	0.5
De Roo	2015	18	0.5
Delaunay	2005	19	0.5
Desmet	2015	13	0.4
Desroches	2016	12	0.3
Dezaly	2011	19	0.5
Duzgun	2011	19	0.5
Duzgun	2014	18	0.5
Ebert	2017	17	0.5
Flurin	2013	19	0.5
Flury	2016	13	0.4
Franceschi	2007	14	0.4

Franceschi	2008	11	0.3
Franceschi	2013	15	0.4
Franceschi	2016	14	0.4
Ganokroj	2019	16	0.4
Garofalo	2010	19	0.5
Gartsman	2004	20	0.5
Gartsman	2013	18	0.5
Gervasi	2016	18	0.5
Ghandour	2019	14	0.4
Gialanella	2011	18	0.5
Grasso	2009	15	0.4
Greiner	2015	14	0.4
Gumina, J Bone Joint	2012	14	0.4
Gumina, Curr Med Res Opin	2012	15	0.4
Han	2013	17	0.5
Hartrick	2012	9	0.2
Hayes	2004	16	0.4
Hollman	2017	14	0.4
Holtby	2016	14	0.4
Hufeland	2019	16	0.4
Iannotti	2006	14	0.4
Ide	2017	15	0.4
Ikemoto	2015	19	0.5
Ilhanli	2015	17	0.5
Jacquot	2014	17	0.5
Jenssen	2018	12	0.3
Jo CH	2013	13	0.4
Jo CH	2014	13	0.4
Jo CH	2015	12	0.3

Keener	2014	14	0.4
Khashan	2016	16	0.4
Kim J	2011	17	0.5
Kim JH	2018	18	0.5
Kim JH	2019	12	0.3
Kim JY	2012	19	0.5
Kim JY	2016	14	0.4
Kim JY	2017	16	0.4
Kim YS	2012	14	0.4
Kim YS	2016	13	0.4
Kim YS	2018	13	0.4
Klintberg	2009	13	0.4
Ko SH	2017	14	0.4
Ko SH	2008	19	0.5
Koh KH	2011	14	0.4
Koh KH	2014	13	0.4
Koh WU	2016	13	0.4
Kraeutler	2015	16	0.4
Kukkonen	2014	16	0.4
Lam	2015	14	0.4
Lamas	2019	12	0.3
Lambers	2015	13	0.4
Lapner	2012	11	0.3
Lastayo	1998	18	0.5
Lee BG	2012	18	0.5
Lee HJ	2015	16	0.4
Lee HJ	2016	14	0.4
Lee JJ	2014	13	0.4
Lee JJ	2015	13	0.4

Lee JJ	2017	11	0.3
Liu, MEDICINE BALT	2017	11	0.3
Liu, J ARTHR AND RE	2017	11	0.3
Ma	2012	15	0.4
MacDonald	2011	12	0.3
Mahure	2017	14	0.4
Malavolta	2014	11	0.3
Malavolta	2018	12	0.3
Malik	2016	17	0.5
Mardani-Kivi	2019	13	0.4
Mazzocca	2017	17	0.5
Menek	2018	14	0.4
Merivirta	2013	14	0.4
Milano	2007	13	0.4
Milano	2010	12	0.3
Milano	2013	12	0.3
Mohtadi	2008	12	0.3
Moosmayer	2010	13	0.4
Moosmayer	2019	11	0.3
Nam	2018	11	0.3
Nicholas	2016	14	0.4
Nicholson	2019	14	0.4
Oh CH	2011	13	0.4
Oh JH	2014	14	0.4
Oh JH	2014	16	0.4
Oh JH	2016	18	0.5
Osbahr	2002	18	0.5
Osti	2013	16	0.4
Osti	2015	15	0.4

Pandey	2016	13	0.4
Park	2015	13	0.4
Park	2016	17	0.5
Perdreau	2015	18	0.5
Piitulainen	2014	11	0.3
Raab	1996	21	0.6
Randelli	2011	13	0.4
Randelli	2015	16	0.4
Randelli	2017	11	0.3
Raschhofer	2017	15	0.4
Rha	2013	12	0.3
Roddey	2002	17	0.5
Rodeo	2012	11	0.3
Ruiz-Moneo	2013	12	0.3
Salviz	2013	12	0.3
Schwartzberg	2013	15	0.4
Senekovic	2017	17	0.5
Seven MM	2017	17	0.5
Shams	2016	21	0.6
Sheps	2015	13	0.4
Sheps	2019	12	0.3
Shibata	2001	19	0.5
Shin SJ	2012	17	0.5
Shin SJ	2012	17	0.5
Syed	2018	15	0.4
Takada	2009	22	0.6
Tetzlaff	2000	24	0.6
Thackeray	2013	20	0.5
Tirefort	2019	13	0.4

Torrens	2019	15	0.4
Van der Zwaal	2013	15	0.4
Walsh	2018	16	0.4
Wang	2015	15	0.4
Watanabe	2016	17	0.5
Weber	2013	17	0.5
Yamakado	2014	18	0.5
Yamamoto	2003	19	0.5
Yun	2012	16	0.4
Zhang	2015	14	0.4
Zumstein	2016	12	0.3

References

1. Abrams, G.D.; Gupta, A.K.; Hussey, K.E.; Tetteh, E.S.; Karas, V.; Bach, B.R.; Cole, B.J.; Romeo, A.A.; Verma, N.N., Arthroscopic repair of full-thickness rotator cuff tears with and without acromioplasty: Randomized prospective trial with 2-year follow-up. *Am J Sports Med* **2014**, *42*, 1296–1303.
2. Dezaly, C.; Sirveaux, F.; Philippe, R.; Wein-Remy, F.; Sedaghatian, J.; Roche, O.; Molé, D., Arthroscopic treatment of rotator cuff tear in the over-60s: Repair is preferable to isolated acromioplasty-tenotomy in the short term. *Orthop Traumatol Surg Res* **2011**, *97*, S125–S130.
3. Jacquot, A.; Dezaly, C.; Goetzmann, T.; Roche, O.; Sirveaux, F.; Molé, D.; French Society for Shoulder, E.b.S., Is rotator cuff repair appropriate in patients older than 60 years of age? Prospective, randomised trial in 103 patients with a mean four-year follow-up. *Orthop Traumatol Surg Res* **2014**, *100*, S333–S338.
4. MacDonald, P.; McRae, S.; Leiter, J.; Mascarenhas, R.; Lapner, P., Arthroscopic rotator cuff repair with and without acromioplasty in the treatment of full-thickness rotator cuff tears: A multicenter, randomized controlled trial. *The Journal of bone and joint surgery. American volume* **2011**, *93*, 1953–1960.
5. Mohtadi, N.G.; Hollinshead, R.M.; Sasyniuk, T.M.; Fletcher, J.A.; Chan, D.S.; Li, F.X., A randomized clinical trial comparing open to arthroscopic acromioplasty with mini-open rotator cuff repair for full-thickness rotator cuff tears: Disease-specific quality of life outcome at an average 2-year follow-up. *Am J Sports Med* **2008**, *36*, 1043–1051.
6. Shin, S.J.; Oh, J.H.; Chung, S.W.; Song, M.H., The efficacy of acromioplasty in the arthroscopic repair of small- to medium-sized rotator cuff tears without acromial spur: Prospective comparative study. *Arthroscopy* **2012**, *28*, 628–635.
7. Cai, Y.; Sun, Z.; Liao, B.; Zhanqiang, S.; Xiao, T.; Zhu, P., Sodium hyaluronate and platelet-rich plasma for partial-thickness rotator cuff tears. *Med Sci Sports Exerc* **2018**.
8. Castricini, R.; Longo, U.G.; De Benedetto, M.; Panfoli, N.; Pirani, P.; Zini, R.; Maffulli, N.; Denaro, V., Platelet-rich plasma augmentation for arthroscopic rotator cuff repair: A randomized controlled trial. *The American journal of sports medicine* **2011**, *39*, 258–265.

9. D'Ambrosi, R.; Palumbo, F.; Paronzini, A.; Ragone, V.; Facchini, R.M., Platelet-rich plasma supplementation in arthroscopic repair of full-thickness rotator cuff tears: A randomized clinical trial. *Musculoskelet Surg* **2016**, *100*, 25–32.
10. Ebert, J.R.; Wang, A.; Smith, A.; Nairn, R.; Breidahl, W.; Zheng, M.H.; Ackland, T., A midterm evaluation of postoperative platelet-rich plasma injections on arthroscopic supraspinatus repair: A randomized controlled trial. *Am J Sports Med* **2017**, *45*, 2965–2974.
11. Flury, M.; Rickenbacher, D.; Schwyzer, H.K.; Jung, C.; Schneider, M.M.; Stahnke, K.; Goldhahn, J.; Audigé, L., Does pure platelet-rich plasma affect postoperative clinical outcomes after arthroscopic rotator cuff repair? A randomized controlled trial. *Am J Sports Med* **2016**, *44*, 2136–2146.
12. Gumina, S.; Campagna, V.; Ferrazza, G.; Giannicola, G.; Fratalocchi, F.; Milani, A.; Postacchini, F., Use of platelet-leukocyte membrane in arthroscopic repair of large rotator cuff tears: A prospective randomized study. *J Bone Joint Surg Am* **2012**, *94*, 1345–1352.
13. Holtby, R.; Christakis, M.; Maman, E.; MacDermid, J.C.; Dwyer, T.; Athwal, G.S.; Faber, K.; Theodoropoulos, J.; Woodhouse, L.J.; Razmjou, H., Impact of platelet-rich plasma on arthroscopic repair of small- to medium-sized rotator cuff tears: A randomized controlled trial. *Orthop J Sports Med* **2016**, *4*, 2325967116665595.
14. Ilhanli, I.; Guder, N.; Gul, M., Platelet-rich plasma treatment with physical therapy in chronic partial supraspinatus tears. *Iran Red Crescent Med J* **2015**, *17*, e23732.
15. Jo, C.H.; Shin, J.S.; Lee, Y.G.; Shin, W.H.; Kim, H.; Lee, S.Y.; Yoon, K.S.; Shin, S., Platelet-rich plasma for arthroscopic repair of large to massive rotator cuff tears: A randomized, single-blind, parallel-group trial. *Am J Sports Med* **2013**, *41*, 2240–2248.
16. Jo, C.H.; Shin, J.S.; Shin, W.H.; Lee, S.Y.; Yoon, K.S.; Shin, S., Platelet-rich plasma for arthroscopic repair of medium to large rotator cuff tears: A randomized controlled trial. *Am J Sports Med* **2015**, *43*, 2102–2110.
17. Malavolta, E.A.; Gracitelli, M.E.; Ferreira Neto, A.A.; Assunção, J.H.; Bordalo-Rodrigues, M.; de Camargo, O.P., Platelet-rich plasma in rotator cuff repair: A prospective randomized study. *Am J Sports Med* **2014**, *42*, 2446–2454.
18. Malavolta, E.A.; Gracitelli, M.E.C.; Assunção, J.H.; Ferreira Neto, A.A.; Bordalo-Rodrigues, M.; de Camargo, O.P., Clinical and structural evaluations of rotator cuff repair with and without added platelet-rich plasma at 5-year follow-up: A prospective randomized study. *Am J Sports Med* **2018**, *46*, 3134–3141.
19. Pandey, V.; Bandi, A.; Madi, S.; Agarwal, L.; Acharya, K.K.; Maddukuri, S.; Sambhaji, C.; Willems, W.J., Does application of moderately concentrated platelet-rich plasma improve clinical and structural outcome after arthroscopic repair of medium-sized to large rotator cuff tear? A randomized controlled trial. *J Shoulder Elbow Surg* **2016**, *25*, 1312–1322.
20. Randelli, P.; Arrigoni, P.; Ragone, V.; Aliprandi, A.; Cabitza, P., Platelet rich plasma in arthroscopic rotator cuff repair: A prospective rct study, 2-year follow-up. *J Shoulder Elbow Surg* **2011**, *20*, 518–528.
21. Rha, D.W.; Park, G.Y.; Kim, Y.K.; Kim, M.T.; Lee, S.C., Comparison of the therapeutic effects of ultrasound-guided platelet-rich plasma injection and dry needling in rotator cuff disease: A randomized controlled trial. *Clin Rehabil* **2013**, *27*, 113–122.
22. Rodeo, S.A.; Delos, D.; Williams, R.J.; Adler, R.S.; Pearle, A.; Warren, R.F., The effect of platelet-rich fibrin matrix on rotator cuff tendon healing: A prospective, randomized clinical study. *Am J Sports Med* **2012**, *40*, 1234–1241.
23. Ruiz-Moneo, P.; Molano-Muñoz, J.; Prieto, E.; Algorta, J., Plasma rich in growth factors in arthroscopic rotator cuff repair: A randomized, double-blind, controlled clinical trial. *Arthroscopy* **2013**, *29*, 2–9.
24. Shams, A.; El-Sayed, M.; Gamal, O.; Ewes, W., Subacromial injection of autologous platelet-rich plasma versus corticosteroid for the treatment of symptomatic partial rotator cuff tears. *Eur J Orthop Surg Traumatol* **2016**, *26*, 837–842.
25. Walsh, M.R.; Nelson, B.J.; Braman, J.P.; Yonke, B.; Obermeier, M.; Raja, A.; Reams, M., Platelet-rich plasma in fibrin matrix to augment rotator cuff repair: A prospective, single-blinded, randomized study with 2-year follow-up. *J Shoulder Elbow Surg* **2018**, *27*, 1553–1563.

26. Wang, A.; McCann, P.; Colliver, J.; Koh, E.; Ackland, T.; Joss, B.; Zheng, M.; Breidahl, B., Do postoperative platelet-rich plasma injections accelerate early tendon healing and functional recovery after arthroscopic supraspinatus repair? A randomized controlled trial. *Am J Sports Med* **2015**, *43*, 1430–1437.
27. Weber, S.C.; Kauffman, J.I.; Parise, C.; Weber, S.J.; Katz, S.D., Platelet-rich fibrin matrix in the management of arthroscopic repair of the rotator cuff: A prospective, randomized, double-blinded study. *Am J Sports Med* **2013**, *41*, 263–270.
28. Zumstein, M.A.; Rumian, A.; Thélu, C.; Lesbats, V.; O'Shea, K.; Schaer, M.; Boileau, P., Secec research grant 2008 ii: Use of platelet- and leucocyte-rich fibrin (l-prf) does not affect late rotator cuff tendon healing: A prospective randomized controlled study. *J Shoulder Elbow Surg* **2016**, *25*, 2–11.
29. Barber, F.A.; Burns, J.P.; Deutsch, A.; Labbé, M.R.; Litchfield, R.B., A prospective, randomized evaluation of acellular human dermal matrix augmentation for arthroscopic rotator cuff repair. *Arthroscopy* **2012**, *28*, 8–15.
30. Cai, Y.Z.; Zhang, C.; Jin, R.L.; Shen, T.; Gu, P.C.; Lin, X.J.; Chen, J.D., Arthroscopic rotator cuff repair with graft augmentation of 3-dimensional biological collagen for moderate to large tears: A randomized controlled study. *Am J Sports Med* **2018**, *46*, 1424–1431.
31. Greiner, S.; Ide, J.; Van Noort, A.; Mochizuki, Y.; Ochi, H.; Marraffino, S.; Sridharan, S.; Rudicel, S.; Itoi, E., Local rhbmp-12 on an absorbable collagen sponge as an adjuvant therapy for rotator cuff repair - a phase 1, randomized, standard of care control, multicenter study: Safety and feasibility. *Am J Sports Med* **2015**, *43*, 1994–2004.
32. Avanzi, P.; Giudici, L.D.; Capone, A.; Cardoni, G.; Lunardi, G.; Foti, G.; Zorzi, C., Prospective randomized controlled trial for patch augmentation in rotator cuff repair: 24-month outcomes. *J Shoulder Elbow Surg* **2019**, *28*, 1918–1927.
33. Lamas, J.R.; García-Fernández, C.; Tornero-Esteban, P.; Lópiz, Y.; Rodriguez-Rodriguez, L.; Ortega, L.; Fernández-Gutiérrez, B.; Marco, F., Adverse effects of xenogenic scaffolding in the context of a randomized double-blind placebo-controlled study for repairing full-thickness rotator cuff tears. *Trials* **2019**, *20*, 387.
34. Burks, R.T.; Crim, J.; Brown, N.; Fink, B.; Greis, P.E., A prospective randomized clinical trial comparing arthroscopic single- and double-row rotator cuff repair: Magnetic resonance imaging and early clinical evaluation. *Am J Sports Med* **2009**, *37*, 674–682.
35. Carbonel, I.; Martinez, A.A.; Calvo, A.; Ripalda, J.; Herrera, A., Single-row versus double-row arthroscopic repair in the treatment of rotator cuff tears: A prospective randomized clinical study. *Int Orthop* **2012**, *36*, 1877–1883.
36. Franceschi, F.; Papalia, R.; Franceschetti, E.; Palumbo, A.; Del Buono, A.; Paciotti, M.; Maffulli, N.; Denaro, V., Double-row repair lowers the retear risk after accelerated rehabilitation. *Am J Sports Med* **2016**, *44*, 948–956.
37. Franceschi, F.; Ruzzini, L.; Longo, U.G.; Martina, F.M.; Zobel, B.B.; Maffulli, N.; Denaro, V., Equivalent clinical results of arthroscopic single-row and double-row suture anchor repair for rotator cuff tears: A randomized controlled trial. *The American journal of sports medicine* **2007**, *35*, 1254–1260.
38. Gartsman, G.M.; Drake, G.; Edwards, T.B.; Elkousy, H.A.; Hammerman, S.M.; O'Connor, D.P.; Press, C.M., Ultrasound evaluation of arthroscopic full-thickness supraspinatus rotator cuff repair: Single-row versus double-row suture bridge (transosseous equivalent) fixation. Results of a prospective, randomized study. *J Shoulder Elbow Surg* **2013**, *22*, 1480–1487.
39. Grasso, A.; Milano, G.; Salvatore, M.; Falcone, G.; Deriu, L.; Fabbriciani, C., Single-row versus double-row arthroscopic rotator cuff repair: A prospective randomized clinical study. *Arthroscopy* **2009**, *25*, 4–12.
40. Kim, Y.S.; Lee, H.J.; Jin, H.K.; Kim, S.E.; Lee, J.W., Conventional en masse repair versus separate double-layer double-row repair for the treatment of delaminated rotator cuff tears. *Am J Sports Med* **2016**, *44*, 1146–1152.
41. Koh, K.H.; Kang, K.C.; Lim, T.K.; Shon, M.S.; Yoo, J.C., Prospective randomized clinical trial of single- versus double-row suture anchor repair in 2- to 4-cm rotator cuff tears: Clinical and magnetic resonance imaging results. *Arthroscopy* **2011**, *27*, 453–462.

42. Lapner, P.L.; Sabri, E.; Rakhra, K.; McRae, S.; Leiter, J.; Bell, K.; Macdonald, P., A multicenter randomized controlled trial comparing single-row with double-row fixation in arthroscopic rotator cuff repair. *J Bone Joint Surg Am* **2012**, *94*, 1249–1257.
43. Ma, H.L.; Chiang, E.R.; Wu, H.T.; Hung, S.C.; Wang, S.T.; Liu, C.L.; Chen, T.H., Clinical outcome and imaging of arthroscopic single-row and double-row rotator cuff repair: A prospective randomized trial. *Arthroscopy* **2012**, *28*, 16–24.
44. Nicholas, S.J.; Lee, S.J.; Mullaney, M.J.; Tyler, T.F.; Fukunaga, T.; Johnson, C.D.; McHugh, M.P., Functional outcomes after double-row versus single-row rotator cuff repair: A prospective randomized trial. *Orthop J Sports Med* **2016**, *4*, 2325967116667398.
45. Iannotti, J.P.; Codsi, M.J.; Kwon, Y.W.; Derwin, K.; Ciccone, J.; Brems, J.J., Porcine small intestine submucosa augmentation of surgical repair of chronic two-tendon rotator cuff tears. A randomized, controlled trial. *J Bone Joint Surg Am* **2006**, *88*, 1238–1244.
46. Ide, J.; Mochizuki, Y.; van Noort, A.; Ochi, H.; Sridharan, S.; Itoi, E.; Greiner, S., Local rhbmp-12 on an absorbable collagen sponge as an adjuvant therapy for rotator cuff repair-a phase 1, randomized, standard of care control, multicenter study: Part 2-a pilot study of functional recovery and structural outcomes. *Orthop J Sports Med* **2017**, *5*, 2325967117726740.
47. Alvarez, C.M.; Litchfield, R.; Jackowski, D.; Griffin, S.; Kirkley, A., A prospective, double-blind, randomized clinical trial comparing subacromial injection of betamethasone and xylocaine to xylocaine alone in chronic rotator cuff tendinosis. *Am J Sports Med* **2005**, *33*, 255–262.
48. Bang, S.R.; Yu, S.K.; Kim, T.H., Can gabapentin help reduce postoperative pain in arthroscopic rotator cuff repair? A prospective, randomized, double-blind study. *Arthroscopy* **2010**, *26*, S106–S111.
49. Barber, F.A.; Herbert, M.A., The effectiveness of an anesthetic continuous-infusion device on postoperative pain control. *Arthroscopy* **2002**, *18*, 76–81.
50. Behr, A.; Freo, U.; Ori, C.; Westermann, B.; Alemanno, F., Buprenorphine added to levobupivacaine enhances postoperative analgesia of middle interscalene brachial plexus block. *J Anesth* **2012**, *26*, 746–751.
51. Borgeat, A.; Aguirre, J.; Marquardt, M.; Mrdjen, J.; Blumenthal, S., Continuous interscalene analgesia with ropivacaine 0.2% versus ropivacaine 0.3% after open rotator cuff repair: The effects on postoperative analgesia and motor function. *Anesth Analg* **2010**, *111*, 1543–1547.
52. Cho, C.H.; Lee, S.W.; Lee, Y.K.; Shin, H.K.; Hwang, I., Effect of a sleep aid in analgesia after arthroscopic rotator cuff repair. *Yonsei Med J* **2015**, *56*, 772–777.
53. Cho, C.H.; Song, K.S.; Min, B.W.; Lee, K.J.; Ha, E.; Lee, Y.C.; Lee, Y.K., Multimodal approach to postoperative pain control in patients undergoing rotator cuff repair. *Knee surgery, sports traumatology, arthroscopy : Official journal of the ESSKA* **2011**, *19*, 1744–1748.
54. Cho, N.S.; Ha, J.H.; Rhee, Y.G., Patient-controlled analgesia after arthroscopic rotator cuff repair: Subacromial catheter versus intravenous injection. *Am J Sports Med* **2007**, *35*, 75–79.
55. Choi, S.; Kim, T.; Kwon, Y.S.; Kang, H., Intra-operative effect of interscalene brachial plexus block to arthroscopic rotator cuff repair surgery. *Int Orthop* **2018**.
56. Chou, W.Y.; Ko, J.Y.; Wang, F.S.; Huang, C.C.; Wong, T.; Wang, C.J.; Chang, H.E., Effect of sodium hyaluronate treatment on rotator cuff lesions without complete tears: A randomized, double-blind, placebo-controlled study. *J Shoulder Elbow Surg* **2010**, *19*, 557–563.
57. Coghlan, J.A.; Forbes, A.; McKenzie, D.; Bell, S.N.; Buchbinder, R., Efficacy of subacromial ropivacaine infusion for rotator cuff surgery. A randomized trial. *J Bone Joint Surg Am* **2009**, *91*, 1558–1567.
58. Culebras, X.; Van Gessel, E.; Hoffmeyer, P.; Gamulin, Z., Clonidine combined with a long acting local anesthetic does not prolong postoperative analgesia after brachial plexus block but does induce hemodynamic changes. *Anesth Analg* **2001**, *92*, 199–204.
59. Delaunay, L.; Souron, V.; Lafosse, L.; Marret, E.; Toussaint, B., Analgesia after arthroscopic rotator cuff repair: Subacromial versus interscalene continuous infusion of ropivacaine. *Reg Anesth Pain Med* **2005**, *30*, 117–122.

60. Desroches, A.; Klouche, S.; Schlur, C.; Bauer, T.; Waitzenegger, T.; Hardy, P., Suprascapular nerve block versus interscalene block as analgesia after arthroscopic rotator cuff repair: A randomized controlled noninferiority trial. *Arthroscopy* **2016**, *32*, 2203–2209.
61. Gumina, S.; Passaretti, D.; Gurzì, M.D.; Candela, V., Arginine l-alpha-ketoglutarate, methylsulfonylmethane, hydrolyzed type i collagen and bromelain in rotator cuff tear repair: A prospective randomized study. *Curr Med Res Opin* **2012**, *28*, 1767–1774.
62. Han, S.S.; Lee, Y.H.; Oh, J.H.; Aminzai, S.; Kim, S.H., Randomized, controlled trial of multimodal shoulder injection or intravenous patient-controlled analgesia after arthroscopic rotator cuff repair. *Knee Surg Sports Traumatol Arthrosc* **2013**, *21*, 2877–2883.
63. Hartrick, C.T.; Tang, Y.S.; Siwek, D.; Murray, R.; Hunstad, D.; Smith, G., The effect of initial local anesthetic dose with continuous interscalene analgesia on postoperative pain and diaphragmatic function in patients undergoing arthroscopic shoulder surgery: A double-blind, randomized controlled trial. *BMC Anesthesiol* **2012**, *12*, 6.
64. Ikemoto, R.Y.; Murachovsky, J.; Prata Nascimento, L.G.; Bueno, R.S.; Oliveira Almeida, L.H.; Strose, E.; de Mello, S.C.; Saletti, D., Prospective randomized study comparing two anesthetic methods for shoulder surgery. *Revista brasileira de ortopedia* **2010**, *45*, 395–399.
65. Jo, C.H.; Shin, J.S.; Huh, J., Multimodal analgesia for arthroscopic rotator cuff repair: A randomized, placebo-controlled, double-blind trial. *Eur J Orthop Surg Traumatol* **2014**, *24*, 315–322.
66. Khashan, M.; Dolkart, O.; Amar, E.; Chechik, O.; Sharfman, Z.; Mozes, G.; Maman, E.; Weinbroum, A., Effect of preemptive intra-articular morphine and ketamine on pain after arthroscopic rotator cuff repair: A prospective, double-blind, randomized controlled study. *Arch Orthop Trauma Surg* **2016**, *136*, 233–239.
67. Kim, J.H.; Koh, H.J.; Kim, D.K.; Lee, H.J.; Kwon, K.H.; Lee, K.Y.; Kim, Y.S., Interscalene brachial plexus bolus block versus patient-controlled interscalene indwelling catheter analgesia for the first 48 hours after arthroscopic rotator cuff repair. *J Shoulder Elbow Surg* **2018**, *27*, 1243–1250.
68. Kim, J.Y.; Song, K.S.; Kim, W.J.; Park, Y.H.; Kang, H.; Woo, Y.C.; Shin, H.Y., Analgesic efficacy of two interscalene blocks and one cervical epidural block in arthroscopic rotator cuff repair. *Knee Surg Sports Traumatol Arthrosc* **2016**, *24*, 931–939.
69. Ko, S.H.; Cho, S.D.; Lee, C.C.; Choi, J.K.; Kim, H.W.; Park, S.J.; Bae, M.H.; Cha, J.R., Comparison of arthroscopically guided suprascapular nerve block and blinded axillary nerve block vs. Blinded suprascapular nerve block in arthroscopic rotator cuff repair: A randomized controlled trial. *Clin Orthop Surg* **2017**, *9*, 340–347.
70. Koh, W.U.; Kim, H.J.; Park, H.S.; Choi, W.J.; Yang, H.S.; Ro, Y.J., A randomised controlled trial comparing continuous supraclavicular and interscalene brachial plexus blockade for open rotator cuff surgery. *Anaesthesia* **2016**, *71*, 692–699.
71. Lee, J.J.; Hwang, J.T.; Kim, D.Y.; Lee, S.S.; Hwang, S.M.; Lee, N.R.; Kwak, B.C., Effects of arthroscopy-guided suprascapular nerve block combined with ultrasound-guided interscalene brachial plexus block for arthroscopic rotator cuff repair: A randomized controlled trial. *Knee Surg Sports Traumatol Arthrosc* **2017**, *25*, 2121–2128.
72. Lee, J.J.; Kim, D.Y.; Hwang, J.T.; Lee, S.S.; Hwang, S.M.; Kim, G.H.; Jo, Y.G., Effect of ultrasonographically guided axillary nerve block combined with suprascapular nerve block in arthroscopic rotator cuff repair: A randomized controlled trial. *Arthroscopy* **2014**, *30*, 906–914.
73. Lee, J.J.; Yoo, Y.S.; Hwang, J.T.; Kim, D.Y.; Jeon, S.J.; Hwang, S.M.; Jang, J.S., Efficacy of direct arthroscopy-guided suprascapular nerve block after arthroscopic rotator cuff repair: A prospective randomized study. *Knee Surg Sports Traumatol Arthrosc* **2015**, *23*, 562–566.
74. Liu, X.N.; Noh, Y.M.; Yang, C.J.; Kim, J.U.; Chung, M.H.; Noh, K.C., Effects of a single-dose interscalene block on pain and stress biomarkers in patients undergoing arthroscopic rotator cuff repair: A randomized controlled trial. *Arthroscopy* **2017**, *33*, 918–926.
75. Malik, T.; Mass, D.; Cohn, S., Postoperative analgesia in a prolonged continuous interscalene block versus single-shot block in outpatient arthroscopic rotator cuff repair: A prospective randomized study. *Arthroscopy* **2016**, *32*, 1544–1550.e1541.

76. Merivirta, R.; Äärimaa, V.; Aantaa, R.; Koivisto, M.; Leino, K.; Liukas, A.; Kuusniemi, K., Postoperative fentanyl patch versus subacromial bupivacaine infusion in arthroscopic shoulder surgery. *Arthroscopy* **2013**, *29*, 1129–1134.
77. Oh, C.H.; Oh, J.H.; Kim, S.H.; Cho, J.H.; Yoon, J.P.; Kim, J.Y., Effectiveness of subacromial anti-adhesive agent injection after arthroscopic rotator cuff repair: Prospective randomized comparison study. *Clin Orthop Surg* **2011**, *3*, 55–61.
78. Osti, L.; Buono, A.D.; Maffulli, N., Pulsed electromagnetic fields after rotator cuff repair: A randomized, controlled study. *Orthopedics* **2015**, *38*, e223–e228.
79. Park, J.Y.; Bang, J.Y.; Oh, K.S., Blind suprascapular and axillary nerve block for post-operative pain in arthroscopic rotator cuff surgery. *Knee Surg Sports Traumatol Arthrosc* **2016**, *24*, 3877–3883.
80. Salviz, E.A.; Xu, D.; Frulla, A.; Kwofie, K.; Shastri, U.; Chen, J.; Shariat, A.N.; Littwin, S.; Lin, E.; Choi, J., *et al.*, Continuous interscalene block in patients having outpatient rotator cuff repair surgery: A prospective randomized trial. *Anesth Analg* **2013**, *117*, 1485–1492.
81. Seven, M.M.; Ersen, O.; Akpancar, S.; Ozkan, H.; Turkkan, S.; Yıldız, Y.; Koca, K., Effectiveness of prolotherapy in the treatment of chronic rotator cuff lesions. *Orthop Traumatol Surg Res* **2017**, *103*, 427–433.
82. Takada, M.; Fukusaki, M.; Terao, Y.; Yamashita, K.; Ando, Y.; Sumikawa, K., Postoperative analgesic effect of preoperative intravenous flurbiprofen in arthroscopic rotator cuff repair. *J Anesth* **2009**, *23*, 500–503.
83. Tetzlaff, J.E.; Brems, J.; Dilger, J., Intraarticular morphine and bupivacaine reduces postoperative pain after rotator cuff repair. *Reg Anesth Pain Med* **2000**, *25*, 611–614.
84. Thackeray, E.M.; Swenson, J.D.; Gertsch, M.C.; Phillips, K.M.; Steele, J.W.; Burks, R.T.; Tashjian, R.Z.; Greis, P.E., Diaphragm function after interscalene brachial plexus block: A double-blind, randomized comparison of 0.25% and 0.125% bupivacaine. *J Shoulder Elbow Surg* **2013**, *22*, 381–386.
85. Watanabe, K.; Tokumine, J.; Yorozu, T.; Moriyama, K.; Sakamoto, H.; Inoue, T., Particulate-steroid betamethasone added to ropivacaine in interscalene brachial plexus block for arthroscopic rotator cuff repair improves postoperative analgesia. *BMC Anesthesiol* **2016**, *16*, 84.
86. Yamakado, K., Efficacy of arthroscopically placed pain catheter adjacent to the suprascapular nerve (continuous arthroscopically assisted suprascapular nerve block) following arthroscopic rotator-cuff repair. *Open Access J Sports Med* **2014**, *5*, 129–136.
87. Yamamoto, S.; Yamaguchi, H.; Sakaguchi, M.; Yamashita, S.; Satsumae, T., Preoperative droperidol improved postoperative pain relief in patients undergoing rotator-cuff repair during general anesthesia using intravenous morphine. *J Clin Anesth* **2003**, *15*, 525–529.
88. Yun, M.J.; Oh, J.H.; Yoon, J.P.; Park, S.H.; Hwang, J.W.; Kil, H.Y., Subacromial patient-controlled analgesia with ropivacaine provides effective pain control after arthroscopic rotator cuff repair. *Knee Surg Sports Traumatol Arthrosc* **2012**, *20*, 1971–1977.
89. Menek, B.; Tarakci, D.; Algun, Z.C., The effect of mulligan mobilization on pain and life quality of patients with rotator cuff syndrome: A randomized controlled trial. *J Back Musculoskelet Rehabil* **2019**, *32*, 171–178.
90. Coory, J.A.; Parr, A.F.; Wilkinson, M.P.; Gupta, A., Efficacy of suprascapular nerve block compared with subacromial injection: A randomized controlled trial in patients with rotator cuff tears. *J Shoulder Elbow Surg* **2019**, *28*, 430–436.
91. Akbaba, Y.A.; Mutlu, E.K.; Altun, S.; Turkmen, E.; Birinci, T.; Celik, D., The effectiveness of trigger point treatment in rotator cuff pathology: A randomized controlled double-blind study. *J Back Musculoskelet Rehabil* **2019**, *32*, 519–527.
92. Banerjee, S.S.; Pulido, P.; Adelson, W.S.; Fronek, J.; Hoenecke, H.R., The efficacy of continuous bupivacaine infiltration following arthroscopic rotator cuff repair. *Arthroscopy* **2008**, *24*, 397–402.
93. Gialanella, B.; Prometti, P., Effects of corticosteroids injection in rotator cuff tears. *Pain Med* **2011**, *12*, 1559–1565.

94. Lee, H.J.; Kim, Y.S.; Park, I.; Ha, D.H.; Lee, J.H., Administration of analgesics after rotator cuff repair: A prospective clinical trial comparing glenohumeral, subacromial, and a combination of glenohumeral and subacromial injections. *J Shoulder Elbow Surg* **2015**, *24*, 663–668.
95. Perdreau, A.; Joudet, T., Efficacy of multimodal analgesia injection combined with corticosteroids after arthroscopic rotator cuff repair. *Orthop Traumatol Surg Res* **2015**, *101*, S337–S345.
96. Ganokroj, P.; Matrakool, L.; Limsuwarn, P.; Sissaynarane, T.; Yimvassana, C.; Laoratanavoraphong, S.; Ratanawarinchai, J., A prospective randomized study comparing the effectiveness of midlateral and posterior subacromial steroid injections. *Orthopedics* **2019**, *42*, e44–e50.
97. Schwartzberg, R.S.; Reuss, B.L.; Rust, R., Efficacy of continuous subacromial bupivacaine infusion for pain control after arthroscopic rotator cuff repair. *J Shoulder Elbow Surg* **2013**, *22*, 1320–1324.
98. Desmet, M.; Vanneste, B.; Reynvoet, M.; Van Cauwelaert, J.; Verhelst, L.; Pottel, H.; Missant, C.; Van de Velde, M., A randomised controlled trial of intravenous dexamethasone combined with interscalene brachial plexus blockade for shoulder surgery. *Anaesthesia* **2015**, *70*, 1180–1185.
99. Kim, J.Y.; Song, S.H.; Cho, J.H.; Cho, H.R., Comparison of clinical efficacy among remifentanil, nicardipine, and remifentanil plus nicardipine continuous infusion for hypotensive anesthesia during arthroscopic shoulder surgery. *J Orthop Surg (Hong Kong)* **2017**, *25*, 2309499017716251.
100. Syed, U.A.M.; Aleem, A.W.; Wowkanech, C.; Weekes, D.; Freedman, M.; Tjoumakaris, F.; Abboud, J.A.; Austin, L.S., Neer award 2018: The effect of preoperative education on opioid consumption in patients undergoing arthroscopic rotator cuff repair: A prospective, randomized clinical trial. *J Shoulder Elbow Surg* **2018**, *27*, 962–967.
101. Kim, Y.S.; Lee, H.J.; Kim, J.H.; Noh, D.Y., When should we repair partial-thickness rotator cuff tears? Outcome comparison between immediate surgical repair versus delayed repair after 6-month period of nonsurgical treatment. *Am J Sports Med* **2018**, *46*, 1091–1096.
102. Moosmayer, S.; Lund, G.; Seljom, U.S.; Haldorsen, B.; Svege, I.C.; Hennig, T.; Pripp, A.H.; Smith, H.J., At a 10-year follow-up, tendon repair is superior to physiotherapy in the treatment of small and medium-sized rotator cuff tears. *J Bone Joint Surg Am* **2019**, *101*, 1050–1060.
103. Lambers Heerspink, F.O.; van Raay, J.J.; Koorevaar, R.C.; van Eerden, P.J.; Westerbeek, R.E.; van 't Riet, E.; van den Akker-Scheek, I.; Diercks, R.L., Comparing surgical repair with conservative treatment for degenerative rotator cuff tears: A randomized controlled trial. *J Shoulder Elbow Surg* **2015**, *24*, 1274–1281.
104. Kukkonen, J.; Joukainen, A.; Lehtinen, J.; Mattila, K.T.; Tuominen, E.K.; Kauko, T.; Äärimaa, V., Treatment of non-traumatic rotator cuff tears: A randomised controlled trial with one-year clinical results. *Bone Joint J* **2014**, *96-B*, 75-81.
105. Moosmayer, S.; Lund, G.; Seljom, U.; Svege, I.; Hennig, T.; Tariq, R.; Smith, H.J., Comparison between surgery and physiotherapy in the treatment of small and medium-sized tears of the rotator cuff: A randomised controlled study of 103 patients with one-year follow-up. *J Bone Joint Surg Br* **2010**, *92*, 83–91.
106. Aagaard, K.E.; Lunsjö, K.; Frobell, R., Early repair of trauma-related full-thickness rotator cuff tears does not eliminate the problem of healing failure. *Bone Joint J* **2019**, *101-B*, 603-609.
107. Analay Akbaba, Y.; Kaya Mutlu, E.; Altun, S.; Celik, D., Does the patients' expectations on kinesiotape affect the outcomes of patients with a rotator cuff tear? A randomized controlled clinical trial. *Clin Rehabil* **2018**, *32*, 1509–1519.
108. Analan, P.D.; Leblebici, B.; Adam, M., Effects of therapeutic ultrasound and exercise on pain, function, and isokinetic shoulder rotator strength of patients with rotator cuff disease. *J Phys Ther Sci* **2015**, *27*, 3113–3117.
109. Arias-Buría, J.L.; Valero-Alcaide, R.; Cleland, J.A.; Salom-Moreno, J.; Ortega-Santiago, R.; Atín-Arratibel, M.A.; Fernández-de-las-Peñas, C., Inclusion of trigger point dry needling in a multimodal physical therapy program for postoperative shoulder pain: A randomized clinical trial. *J Manipulative Physiol Ther* **2015**, *38*, 179–187.

110. Arndt, J.; Clavert, P.; Mielcarek, P.; Bouchaib, J.; Meyer, N.; Kempf, J.F.; (SOFEC), F.S.f.S.E., Immediate passive motion versus immobilization after endoscopic supraspinatus tendon repair: A prospective randomized study. *Orthop Traumatol Surg Res* **2012**, *98*, S131–S138.
111. Baumgarten, K.M.; Osborn, R.; Schweinle, W.E.; Zens, M.J.; Helsper, E.A., Are pulley exercises initiated 6 weeks after rotator cuff repair a safe and effective rehabilitative treatment? A randomized controlled trial. *Am J Sports Med* **2016**, *44*, 1844–1851.
112. Blum, K.; Chen, A.L.; Chen, T.J.; Waite, R.L.; Downs, B.W.; Braverman, E.R.; Kerner, M.M.; Savarimuthu, S.M.; DiNubile, N., Repetitive h-wave device stimulation and program induces significant increases in the range of motion of post operative rotator cuff reconstruction in a double-blinded randomized placebo controlled human study. *BMC Musculoskelet Disord* **2009**, *10*, 132.
113. Cuff, D.J.; Pupello, D.R., Prospective randomized study of arthroscopic rotator cuff repair using an early versus delayed postoperative physical therapy protocol. *J Shoulder Elbow Surg* **2012**, *21*, 1450–1455.
114. De Roo, P.J.; Muermans, S.; Maroy, M.; Linden, P.; Van den Daelen, L., Passive mobilization after arthroscopic rotator cuff repair is not detrimental in the early postoperative period. *Acta Orthop Belg* **2015**, *81*, 485–492.
115. Düzgün, I.; Baltacı, G.; Atay, O.A., Comparison of slow and accelerated rehabilitation protocol after arthroscopic rotator cuff repair: Pain and functional activity. *Acta Orthop Traumatol Turc* **2011**, *45*, 23–33.
116. Düzgün, İ.; Baltacı, G.; Turgut, E.; Atay, O.A., Effects of slow and accelerated rehabilitation protocols on range of motion after arthroscopic rotator cuff repair. *Acta Orthop Traumatol Turc* **2014**, *48*, 642–648.
117. Hayes, K.; Ginn, K.A.; Walton, J.R.; Szomor, Z.L.; Murrell, G.A., A randomised clinical trial evaluating the efficacy of physiotherapy after rotator cuff repair. *Aust J Physiother* **2004**, *50*, 77–83.
118. Jenssen, K.K.; Lundgreen, K.; Madsen, J.E.; Kvakestad, R.; Pripp, A.H.; Dimmen, S., No functional difference between three and six weeks of immobilization after arthroscopic rotator cuff repair: A prospective randomized controlled non-inferiority trial. *Arthroscopy* **2018**, *34*, 2765–2774.
119. Keener, J.D.; Galatz, L.M.; Stobbs-Cucchi, G.; Patton, R.; Yamaguchi, K., Rehabilitation following arthroscopic rotator cuff repair: A prospective randomized trial of immobilization compared with early motion. *J Bone Joint Surg Am* **2014**, *96*, 11–19.
120. Kim, J.Y.; Lee, J.S.; Park, C.W., Extracorporeal shock wave therapy is not useful after arthroscopic rotator cuff repair. *Knee Surg Sports Traumatol Arthrosc* **2012**, *20*, 2567–2572.
121. Kim, Y.S.; Chung, S.W.; Kim, J.Y.; Ok, J.H.; Park, I.; Oh, J.H., Is early passive motion exercise necessary after arthroscopic rotator cuff repair? *Am J Sports Med* **2012**, *40*, 815–821.
122. Koh, K.H.; Lim, T.K.; Shon, M.S.; Park, Y.E.; Lee, S.W.; Yoo, J.C., Effect of immobilization without passive exercise after rotator cuff repair: Randomized clinical trial comparing four and eight weeks of immobilization. *J Bone Joint Surg Am* **2014**, *96*, e44.
123. Kraeutler, M.J.; Reynolds, K.A.; Long, C.; McCarty, E.C., Compressive cryotherapy versus ice-a prospective, randomized study on postoperative pain in patients undergoing arthroscopic rotator cuff repair or subacromial decompression. *J Shoulder Elbow Surg* **2015**, *24*, 854–859.
124. Lam, P.H.; Hansen, K.; Keighley, G.; Hackett, L.; Murrell, G.A., A randomized, double-blinded, placebo-controlled clinical trial evaluating the effectiveness of daily vibration after arthroscopic rotator cuff repair. *Am J Sports Med* **2015**, *43*, 2774–2782.
125. Lastayo, P.C.; Wright, T.; Jaffe, R.; Hartzel, J., Continuous passive motion after repair of the rotator cuff. A prospective outcome study. *J Bone Joint Surg Am* **1998**, *80*, 1002–1011.
126. Lee, B.G.; Cho, N.S.; Rhee, Y.G., Effect of two rehabilitation protocols on range of motion and healing rates after arthroscopic rotator cuff repair: Aggressive versus limited early passive exercises. *Arthroscopy* **2012**, *28*, 34–42.

127. Mahure, S.A.; Rokito, A.S.; Kwon, Y.W., Transcutaneous electrical nerve stimulation for postoperative pain relief after arthroscopic rotator cuff repair: A prospective double-blinded randomized trial. *J Shoulder Elbow Surg* **2017**, *26*, 1508–1513.
128. Mazzocca, A.D.; Arciero, R.A.; Shea, K.P.; Apostolakos, J.M.; Solovyova, O.; Gomlinski, G.; Wojcik, K.E.; Tafuto, V.; Stock, H.; Cote, M.P., The effect of early range of motion on quality of life, clinical outcome, and repair integrity after arthroscopic rotator cuff repair. *Arthroscopy* **2017**, *33*, 1138–1148.
129. Osbahr, D.C.; Cawley, P.W.; Speer, K.P., The effect of continuous cryotherapy on glenohumeral joint and subacromial space temperatures in the postoperative shoulder. *Arthroscopy* **2002**, *18*, 748–754.
130. Piitulainen, K.; Häkkinen, A.; Salo, P.; Kautiainen, H.; Ylinen, J., Does adding a 12-month exercise programme to usual care after a rotator cuff repair effect disability and quality of life at 12 months? A randomized controlled trial. *Clin Rehabil* **2015**, *29*, 447–456.
131. Raab, M.G.; Rzeszutko, D.; O'Connor, W.; Greatting, M.D., Early results of continuous passive motion after rotator cuff repair: A prospective, randomized, blinded, controlled study. *Am J Orthop (Belle Mead NJ)* **1996**, *25*, 214–220.
132. Roddey, T.S.; Olson, S.L.; Gartsman, G.M.; Hanten, W.P.; Cook, K.F., A randomized controlled trial comparing 2 instructional approaches to home exercise instruction following arthroscopic full-thickness rotator cuff repair surgery. *J Orthop Sports Phys Ther* **2002**, *32*, 548–559.
133. Sheps, D.M.; Silveira, A.; Beaupre, L.; Styles-Tripp, F.; Balyk, R.; Lalani, A.; Glasgow, R.; Bergman, J.; Bouliane, M.; (SURGE), S.a.U.E.R.G.o.E., Early active motion versus sling immobilization after arthroscopic rotator cuff repair: A randomized controlled trial. *Arthroscopy* **2019**, *35*, 749–760.e742.
134. Sheps, D.M.; Bouliane, M.; Styles-Tripp, F.; Beaupre, L.A.; Saraswat, M.K.; Luciak-Corea, C.; Silveira, A.; Glasgow, R.; Balyk, R., Early mobilisation following mini-open rotator cuff repair: A randomised control trial. *Bone Joint J* **2015**, *97-B*, 1257-1263.
135. Garofalo, R.; Conti, M.; Notarnicola, A.; Maradei, L.; Giardella, A.; Castagna, A., Effects of one-month continuous passive motion after arthroscopic rotator cuff repair: Results at 1-year follow-up of a prospective randomized study. *Musculoskeletal surgery* **2010**, *94 Suppl 1*, S79-83.
136. Klintberg, I.H.; Gunnarsson, A.C.; Svantesson, U.; Styf, J.; Karlsson, J., Early loading in physiotherapy treatment after full-thickness rotator cuff repair: A prospective randomized pilot-study with a two-year follow-up. *Clinical rehabilitation* **2009**, *23*, 622–638.
137. Raschhofer, R.; Poulios, N.; Schimetta, W.; Kisling, R.; Mittermaier, C., Early active rehabilitation after arthroscopic rotator cuff repair: A prospective randomized pilot study. *Clinical rehabilitation* **2017**, *31*, 1332–1339.
138. De Carli, A.; Vadalà, A.; Zanzotto, E.; Zampar, G.; Vetrano, M.; Iorio, R.; Ferretti, A., Reparable rotator cuff tears with concomitant long-head biceps lesions: Tenotomy or tenotomy/tenodesis? *Knee Surg Sports Traumatol Arthrosc* **2012**, *20*, 2553–2558.
139. Lee, H.J.; Jeong, J.Y.; Kim, C.K.; Kim, Y.S., Surgical treatment of lesions of the long head of the biceps brachii tendon with rotator cuff tear: A prospective randomized clinical trial comparing the clinical results of tenotomy and tenodesis. *J Shoulder Elbow Surg* **2016**, *25*, 1107–1114.
140. Oh, J.H.; Lee, Y.H.; Kim, S.H.; Park, J.S.; Seo, H.J.; Kim, W.; Park, H.B., Comparison of treatments for superior labrum-biceps complex lesions with concomitant rotator cuff repair: A prospective, randomized, comparative analysis of debridement, biceps tenotomy, and biceps tenodesis. *Arthroscopy* **2016**, *32*, 958–967.
141. Zhang, Q.; Zhou, J.; Ge, H.; Cheng, B., Tenotomy or tenodesis for long head biceps lesions in shoulders with reparable rotator cuff tears: A prospective randomised trial. *Knee Surg Sports Traumatol Arthrosc* **2015**, *23*, 464–469.
142. Belay, E.S.; Wittstein, J.R.; Garrigues, G.E.; Lassiter, T.E.; Scribani, M.; Goldner, R.D.; Bean, C.A., Biceps tenotomy has earlier pain relief compared to biceps tenodesis: A randomized prospective study. *Knee Surg Sports Traumatol Arthrosc* **2019**, *27*, 4032–4037.
143. Hufeland, M.; Wicke, S.; Verde, P.E.; Krauspe, R.; Patzer, T., Biceps tenodesis versus tenotomy in isolated lhb lesions: A prospective randomized clinical trial. *Arch Orthop Trauma Surg* **2019**, *139*, 961–970.

144. Carr, A.; Cooper, C.; Campbell, M.K.; Rees, J.; Moser, J.; Beard, D.J.; Fitzpatrick, R.; Gray, A.; Dawson, J.; Murphy, J., *et al.*, Effectiveness of open and arthroscopic rotator cuff repair (ukuff): A randomised controlled trial. *Bone Joint J* **2017**, *99-B*, 107-115.
145. Cho, C.H.; Song, K.S.; Jung, G.H.; Lee, Y.K.; Shin, H.K., Early postoperative outcomes between arthroscopic and mini-open repair for rotator cuff tears. *Orthopedics* **2012**, *35*, e1347–e1352.
146. Liu, J.; Fan, L.; Zhu, Y.; Yu, H.; Xu, T.; Li, G., Comparison of clinical outcomes in all-arthroscopic versus mini-open repair of rotator cuff tears: A randomized clinical trial. *Medicine (Baltimore)* **2017**, *96*, e6322.
147. van der Zwaal, P.; Thomassen, B.J.; Nieuwenhuijse, M.J.; Lindenburg, R.; Swen, J.W.; van Arkel, E.R., Clinical outcome in all-arthroscopic versus mini-open rotator cuff repair in small to medium-sized tears: A randomized controlled trial in 100 patients with 1-year follow-up. *Arthroscopy* **2013**, *29*, 266–273.
148. Bidwai, A.S.; Birch, A.; Temperley, D.; Odak, S.; Walton, M.J.; Haines, J.F.; Trail, I., Medium- to long-term results of a randomized controlled trial to assess the efficacy of arthoscopic-subacromial decompression versus mini-open repair for the treatment of medium-sized rotator cuff tears. *Shoulder Elbow* **2016**, *8*, 101–105.
149. Flurin, P.H.; Hardy, P.; Abadie, P.; Desmoineaux, P.; Essig, J.; Joudet, T.; Sommaire, C.; Thelu, C.E.; (SFA), F.A.S., Rotator cuff tears after 70 years of age: A prospective, randomized, comparative study between decompression and arthroscopic repair in 154 patients. *Orthop Traumatol Surg Res* **2013**, *99*, S371–S378.
150. Gartsman, G.M.; O'connor, D.P., Arthroscopic rotator cuff repair with and without arthroscopic subacromial decompression: A prospective, randomized study of one-year outcomes. *J Shoulder Elbow Surg* **2004**, *13*, 424–426.
151. Milano, G.; Grasso, A.; Salvatore, M.; Zarelli, D.; Deriu, L.; Fabbriciani, C., Arthroscopic rotator cuff repair with and without subacromial decompression: A prospective randomized study. *Arthroscopy : The journal of arthroscopic & related surgery : Official publication of the Arthroscopy Association of North America and the International Arthroscopy Association* **2007**, *23*, 81–88.
152. Nam, J.H.; Park, S.; Lee, H.R.; Kim, S.H., Outcomes after limited or extensive bursectomy during rotator cuff repair: Randomized controlled trial. *Arthroscopy* **2018**.
153. Boehm, T.D.; Werner, A.; Radtke, S.; Mueller, T.; Kirschner, S.; Gohlke, F., The effect of suture materials and techniques on the outcome of repair of the rotator cuff: A prospective, randomised study. *J Bone Joint Surg Br* **2005**, *87*, 819–823.
154. Randelli, P.; Arrigoni, P.; Aliprandi, A.; Sdao, S.; Ragone, V.; D'Ambrosi, R.; Randelli, F.; Cabitza, P.; Banfi, G., Repair versus shaving of partial-thickness articular-sided tears of the upper subscapularis tendon. A prospective randomized controlled trial. *Joints* **2015**, *3*, 109–115.
155. Bigoni, M.; Gorla, M.; Guerrasio, S.; Brignoli, A.; Cossio, A.; Grillo, P.; Marinoni, E.C., Shoulder evaluation with isokinetic strength testing after arthroscopic rotator cuff repairs. *J Shoulder Elbow Surg* **2009**, *18*, 178–183.
156. Franceschi, F.; Papalia, R.; Del Buono, A.; Vasta, S.; Costa, V.; Maffulli, N.; Denaro, V., Articular-sided rotator cuff tears: Which is the best repair? A three-year prospective randomised controlled trial. *Int Orthop* **2013**, *37*, 1487–1493.
157. Ko, S.H.; Lee, C.C.; Friedman, D.; Park, K.B.; Warner, J.J., Arthroscopic single-row supraspinatus tendon repair with a modified mattress locking stitch: A prospective, randomized controlled comparison with a simple stitch. *Arthroscopy* **2008**, *24*, 1005–1012.
158. Milano, G.; Grasso, A.; Salvatore, M.; Saccomanno, M.F.; Deriu, L.; Fabbriciani, C., Arthroscopic rotator cuff repair with metal and biodegradable suture anchors: A prospective randomized study. *Arthroscopy* **2010**, *26*, S112–S119.
159. Shin, S.J., A comparison of 2 repair techniques for partial-thickness articular-sided rotator cuff tears. *Arthroscopy* **2012**, *28*, 25–33.
160. Mardani-Kivi, M.; Keyhani, S.; Ebrahim-Zadeh, M.H.; Hashemi-Motlagh, K.; Saheb-Ekhtiari, K., Rotator cuff tear with concomitant long head of biceps tendon (lhbt) degeneration: What is the preferred choice? Open subpectoral versus arthroscopic intraarticular tenodesis. *J Orthop Traumatol* **2019**, *20*, 26.
161. Randelli, P.; Stoppani, C.A.; Zaolino, C.; Menon, A.; Randelli, F.; Cabitza, P., Advantages of arthroscopic rotator cuff repair with a transosseous suture technique: A prospective randomized controlled trial. *Am J Sports Med* **2017**, *45*, 2000–2009.

162. Kim, J.H.; Ha, D.H.; Kim, S.M.; Kim, K.W.; Han, S.Y.; Kim, Y.S., Does arthroscopic preemptive extensive rotator interval release reduce postoperative stiffness after arthroscopic rotator cuff repair?: A prospective randomized clinical trial. *J Shoulder Elbow Surg* **2019**, *28*, 1639–1646.
163. Berth, A.; Neumann, W.; Awiszus, F.; Pap, G., Massive rotator cuff tears: Functional outcome after debridement or arthroscopic partial repair. *J Orthop Traumatol* **2010**, *11*, 13–20.
164. Blanchard, T.K.; Bearcroft, P.W.; Constant, C.R.; Griffin, D.R.; Dixon, A.K., Diagnostic and therapeutic impact of mri and arthrography in the investigation of full-thickness rotator cuff tears. *Eur Radiol* **1999**, *9*, 638–642.
165. Capito, N.M.; Cook, J.L.; Yahuaca, B.; Capito, M.D.; Sherman, S.L.; Smith, M.J., Safety and efficacy of hyperosmolar irrigation solution in shoulder arthroscopy. *J Shoulder Elbow Surg* **2017**, *26*, 745–751.
166. Castagna, A.; Borroni, M.; Garofalo, R.; Rose, G.D.; Cesari, E.; Padua, R.; Conti, M.; Gumina, S., Deep partial rotator cuff tear: Transtendon repair or tear completion and repair? A randomized clinical trial. *Knee Surg Sports Traumatol Arthrosc* **2015**, *23*, 460–463.
167. Chierichini, A.; Frassanito, L.; Vergari, A.; Santoprete, S.; Chiarotti, F.; Saccomanno, M.F.; Milano, G., The effect of norepinephrine versus epinephrine in irrigation fluid on the incidence of hypotensive/bradycardic events during arthroscopic rotator cuff repair with interscalene block in the sitting position. *Arthroscopy* **2015**, *31*, 800–806.
168. Franceschi, F.; Longo, U.G.; Ruzzini, L.; Rizzello, G.; Maffulli, N.; Denaro, V., No advantages in repairing a type ii superior labrum anterior and posterior (slap) lesion when associated with rotator cuff repair in patients over age 50: A randomized controlled trial. *The American journal of sports medicine* **2008**, *36*, 247–253.
169. Gervasi, E.; Maman, E.; Dekel, A.; Cautero, E., Fluoroscopy-guided biodegradable spacer implantation using local anesthesia: Safety and efficacy study in patients with massive rotator cuff tears. *Musculoskelet Surg* **2016**, *100*, 19–24.
170. Kim, J.; Chung, J.; Ok, H., Asymptomatic acromioclavicular joint arthritis in arthroscopic rotator cuff tendon repair: A prospective randomized comparison study. *Arch Orthop Trauma Surg* **2011**, *131*, 363–369.
171. Milano, G.; Saccomanno, M.F.; Careri, S.; Taccardo, G.; De Vitis, R.; Fabbriciani, C., Efficacy of marrow-stimulating technique in arthroscopic rotator cuff repair: A prospective randomized study. *Arthroscopy* **2013**, *29*, 802–810.
172. Oh, J.H.; Kim, J.Y.; Choi, J.H.; Park, S.M., Is arthroscopic distal clavicle resection necessary for patients with radiological acromioclavicular joint arthritis and rotator cuff tears? A prospective randomized comparative study. *Am J Sports Med* **2014**, *42*, 2567–2573.
173. Oh, J.H.; Kim, J.Y.; Chung, S.W.; Park, J.S.; Kim, D.H.; Kim, S.H.; Yun, M.J., Warmed irrigation fluid does not decrease perioperative hypothermia during arthroscopic shoulder surgery. *Arthroscopy* **2014**, *30*, 159–164.
174. Osti, L.; Del Buono, A.; Maffulli, N., Microfractures at the rotator cuff footprint: A randomised controlled study. *Int Orthop* **2013**, *37*, 2165–2171.
175. Park, Y.B.; Koh, K.H.; Shon, M.S.; Park, Y.E.; Yoo, J.C., Arthroscopic distal clavicle resection in symptomatic acromioclavicular joint arthritis combined with rotator cuff tear: A prospective randomized trial. *Am J Sports Med* **2015**, *43*, 985–990.
176. Senekovic, V.; Poberaj, B.; Kovacic, L.; Mikek, M.; Adar, E.; Markovitz, E.; Maman, E.; Dekel, A., The biodegradable spacer as a novel treatment modality for massive rotator cuff tears: A prospective study with 5-year follow-up. *Arch Orthop Trauma Surg* **2017**, *137*, 95–103.
177. Torrens, C.; Miquel, J.; Santana, F., Do we really allow patient decision-making in rotator cuff surgery? A prospective randomized study. *J Orthop Surg Res* **2019**, *14*, 116.
178. Nicholson, J.A.; Searle, H.K.C.; MacDonald, D.; McBirnie, J., Cost-effectiveness and satisfaction following arthroscopic rotator cuff repair: Does age matter? *Bone Joint J* **2019**, *101-B*, 860-866.

179. Shibata, Y.; Midorikawa, K.; Emoto, G.; Naito, M., Clinical evaluation of sodium hyaluronate for the treatment of patients with rotator cuff tear. *J Shoulder Elbow Surg* **2001**, *10*, 209–216.
180. Conti, M.; Garofalo, R.; Castagna, A., Does a brace influence clinical outcomes after arthroscopic rotator cuff repair? *Musculoskelet Surg* **2015**, *99 Suppl 1*, S31-35.
181. Hollman, F.; Wolterbeek, N.; Zijl, J.A.C.; van Egeraat, S.P.M.; Wessel, R.N., Abduction brace versus antirotation sling after arthroscopic cuff repair: The effects on pain and function. *Arthroscopy* **2017**, *33*, 1618–1626.
182. Ghandour, T.M.; Ibrahim, A.; Abdelrahman, A.A.; Elgammal, A.; Hammad, M.H., Does the type of shoulder brace affect postoperative pain and clinical outcome after arthroscopic rotator cuff repair? *Arthroscopy* **2019**, *35*, 1016–1023.
183. Tirefort, J.; Schwitzguebel, A.J.; Collin, P.; Nowak, A.; Plomb-Holmes, C.; Ladermann, A., Postoperative mobilization after superior rotator cuff repair: Sling versus no sling: A randomized prospective study. *The Journal of bone and joint surgery. American volume* **2019**, *101*, 494–503.

184. 1. Abrams, G.D.; Gupta, A.K.; Hussey, K.E.; Tetteh, E.S.; Karas, V.; Bach, B.R.; Cole, B.J.; Romeo, A.A.; Verma, N.N., Arthroscopic repair of full-thickness rotator cuff tears with and without acromioplasty: Randomized prospective trial with 2-year follow-up. *Am J Sports Med* **2014**, *42*, 1296-1303.
185. 2. Dezaly, C.; Sirveaux, F.; Philippe, R.; Wein-Remy, F.; Sedaghatian, J.; Roche, O.; Molé, D., Arthroscopic treatment of rotator cuff tear in the over-60s: Repair is preferable to isolated acromioplasty-tenotomy in the short term. *Orthop Traumatol Surg Res* **2011**, *97*, S125-130.
186. 3. Jacquot, A.; Dezaly, C.; Goetzmann, T.; Roche, O.; Sirveaux, F.; Molé, D.; French Society for Shoulder, E.b.S., Is rotator cuff repair appropriate in patients older than 60 years of age? Prospective, randomised trial in 103 patients with a mean four-year follow-up. *Orthop Traumatol Surg Res* **2014**, *100*, S333-338.
187. 4. MacDonald, P.; McRae, S.; Leiter, J.; Mascarenhas, R.; Lapner, P., Arthroscopic rotator cuff repair with and without acromioplasty in the treatment of full-thickness rotator cuff tears: A multicenter, randomized controlled trial. *The Journal of bone and joint surgery. American volume* **2011**, *93*, 1953-1960.
188. 5. Mohtadi, N.G.; Hollinshead, R.M.; Sasyniuk, T.M.; Fletcher, J.A.; Chan, D.S.; Li, F.X., A randomized clinical trial comparing open to arthroscopic acromioplasty with mini-open rotator cuff repair for full-thickness rotator cuff tears: Disease-specific quality of life outcome at an average 2-year follow-up. *Am J Sports Med* **2008**, *36*, 1043-1051.
189. 6. Shin, S.J.; Oh, J.H.; Chung, S.W.; Song, M.H., The efficacy of acromioplasty in the arthroscopic repair of small- to medium-sized rotator cuff tears without acromial spur: Prospective comparative study. *Arthroscopy* **2012**, *28*, 628-635.
190. 7. Cai, Y.; Sun, Z.; Liao, B.; Zhanqiang, S.; Xiao, T.; Zhu, P., Sodium hyaluronate and platelet-rich plasma for partial-thickness rotator cuff tears. *Med Sci Sports Exerc* **2018**.
191. 8. Castricini, R.; Longo, U.G.; De Benedetto, M.; Panfoli, N.; Pirani, P.; Zini, R.; Maffulli, N.; Denaro, V., Platelet-rich plasma augmentation for arthroscopic rotator cuff repair: A randomized controlled trial. *Am J Sports Med* **2011**, *39*, 258-265.
192. 9. D'Ambrosi, R.; Palumbo, F.; Paronzini, A.; Ragone, V.; Facchini, R.M., Platelet-rich plasma supplementation in arthroscopic repair of full-thickness rotator cuff tears: A randomized clinical trial. *Musculoskelet Surg* **2016**, *100*, 25-32.

193. 10. Ebert, J.R.; Wang, A.; Smith, A.; Nairn, R.; Breidahl, W.; Zheng, M.H.; Ackland, T., A midterm evaluation of postoperative platelet-rich plasma injections on arthroscopic supraspinatus repair: A randomized controlled trial. *Am J Sports Med* **2017**, *45*, 2965-2974.

194. 11. Flury, M.; Rickenbacher, D.; Schwyzer, H.K.; Jung, C.; Schneider, M.M.; Stahnke, K.; Goldhahn, J.; Audigé, L., Does pure platelet-rich plasma affect postoperative clinical outcomes after arthroscopic rotator cuff repair? A randomized controlled trial. *Am J Sports Med* **2016**, *44*, 2136-2146.

195. 12. Gumina, S.; Campagna, V.; Ferrazza, G.; Giannicola, G.; Fratalocchi, F.; Milani, A.; Postacchini, F., Use of platelet-leukocyte membrane in arthroscopic repair of large rotator cuff tears: A prospective randomized study. *The Journal of bone and joint surgery. American volume* **2012**, *94*, 1345-1352.

196. 13. Holtby, R.; Christakis, M.; Maman, E.; MacDermid, J.C.; Dwyer, T.; Athwal, G.S.; Faber, K.; Theodoropoulos, J.; Woodhouse, L.J.; Razmjou, H., Impact of platelet-rich plasma on arthroscopic repair of small- to medium-sized rotator cuff tears: A randomized controlled trial. *Orthop J Sports Med* **2016**, *4*, 2325967116665595.

197. 14. Ilhanli, I.; Guder, N.; Gul, M., Platelet-rich plasma treatment with physical therapy in chronic partial supraspinatus tears. *Iran Red Crescent Med J* **2015**, *17*, e23732.

198. 15. Jo, C.H.; Shin, J.S.; Lee, Y.G.; Shin, W.H.; Kim, H.; Lee, S.Y.; Yoon, K.S.; Shin, S., Platelet-rich plasma for arthroscopic repair of large to massive rotator cuff tears: A randomized, single-blind, parallel-group trial. *Am J Sports Med* **2013**, *41*, 2240-2248.

199. 16. Jo, C.H.; Shin, J.S.; Shin, W.H.; Lee, S.Y.; Yoon, K.S.; Shin, S., Platelet-rich plasma for arthroscopic repair of medium to large rotator cuff tears: A randomized controlled trial. *Am J Sports Med* **2015**, *43*, 2102-2110.

200. 17. Malavolta, E.A.; Gracitelli, M.E.; Ferreira Neto, A.A.; Assunção, J.H.; Bordalo-Rodrigues, M.; de Camargo, O.P., Platelet-rich plasma in rotator cuff repair: A prospective randomized study. *Am J Sports Med* **2014**, *42*, 2446-2454.

201. 18. Malavolta, E.A.; Gracitelli, M.E.C.; Assunção, J.H.; Ferreira Neto, A.A.; Bordalo-Rodrigues, M.; de Camargo, O.P., Clinical and structural evaluations of rotator cuff repair with and without added platelet-rich plasma at 5-year follow-up: A prospective randomized study. *Am J Sports Med* **2018**, *46*, 3134-3141.

202. 19. Pandey, V.; Bandi, A.; Madi, S.; Agarwal, L.; Acharya, K.K.; Maddukuri, S.; Sambhaji, C.; Willems, W.J., Does application of moderately concentrated platelet-rich plasma improve clinical and structural outcome after arthroscopic repair of medium-sized to large rotator cuff tear? A randomized controlled trial. *J Shoulder Elbow Surg* **2016**, *25*, 1312-1322.

203. 20. Randelli, P.; Arrigoni, P.; Ragone, V.; Aliprandi, A.; Cabitza, P., Platelet rich plasma in arthroscopic rotator cuff repair: A prospective rct study, 2-year follow-up. *J Shoulder Elbow Surg* **2011**, *20*, 518-528.

204. 21. Rha, D.W.; Park, G.Y.; Kim, Y.K.; Kim, M.T.; Lee, S.C., Comparison of the therapeutic effects of ultrasound-guided platelet-rich plasma injection and dry needling in rotator cuff disease: A randomized controlled trial. *Clin Rehabil* **2013**, *27*, 113-122.

205. 22. Rodeo, S.A.; Delos, D.; Williams, R.J.; Adler, R.S.; Pearle, A.; Warren, R.F., The effect of platelet-rich fibrin matrix on rotator cuff tendon healing: A prospective, randomized clinical study. *Am J Sports Med* **2012**, *40*, 1234-1241.

206. 23. Ruiz-Moneo, P.; Molano-Muñoz, J.; Prieto, E.; Algorta, J., Plasma rich in growth factors in arthroscopic rotator cuff repair: A randomized, double-blind, controlled clinical trial. *Arthroscopy* **2013**, *29*, 2-9.

207. 24. Shams, A.; El-Sayed, M.; Gamal, O.; Ewes, W., Subacromial injection of autologous platelet-rich plasma versus corticosteroid for the treatment of symptomatic partial rotator cuff tears. *Eur J Orthop Surg Traumatol* **2016**, *26*, 837-842.

208. 25. Walsh, M.R.; Nelson, B.J.; Braman, J.P.; Yonke, B.; Obermeier, M.; Raja, A.; Reams, M., Platelet-rich plasma in fibrin matrix to augment rotator cuff repair: A prospective, single-blinded, randomized study with 2-year follow-up. *J Shoulder Elbow Surg* **2018**, *27*, 1553-1563.

209. 26. Wang, A.; McCann, P.; Colliver, J.; Koh, E.; Ackland, T.; Joss, B.; Zheng, M.; Breidahl, B., Do postoperative platelet-rich plasma injections accelerate early tendon healing and functional recovery after arthroscopic supraspinatus repair? A randomized controlled trial. *Am J Sports Med* **2015**, *43*, 1430-1437.

210. 27. Weber, S.C.; Kauffman, J.I.; Parise, C.; Weber, S.J.; Katz, S.D., Platelet-rich fibrin matrix in the management of arthroscopic repair of the rotator cuff: A prospective, randomized, double-blinded study. *Am J Sports Med* **2013**, *41*, 263-270.

211. 28. Zumstein, M.A.; Rumian, A.; Thélu, C.; Lesbats, V.; O'Shea, K.; Schaer, M.; Boileau, P., Secec research grant 2008 ii: Use of platelet- and leucocyte-rich fibrin (l-prf) does not affect late rotator cuff tendon healing: A prospective randomized controlled study. *J Shoulder Elbow Surg* **2016**, *25*, 2-11.

212. 29. Barber, F.A.; Burns, J.P.; Deutsch, A.; Labbé, M.R.; Litchfield, R.B., A prospective, randomized evaluation of acellular human dermal matrix augmentation for arthroscopic rotator cuff repair. *Arthroscopy* **2012**, *28*, 8-15.

213. 30. Cai, Y.Z.; Zhang, C.; Jin, R.L.; Shen, T.; Gu, P.C.; Lin, X.J.; Chen, J.D., Arthroscopic rotator cuff repair with graft augmentation of 3-dimensional biological collagen for moderate to large tears: A randomized controlled study. *Am J Sports Med* **2018**, *46*, 1424-1431.

214. 31. Greiner, S.; Ide, J.; Van Noort, A.; Mochizuki, Y.; Ochi, H.; Marraffino, S.; Sridharan, S.; Rudicel, S.; Itoi, E., Local rhbmp-12 on an absorbable collagen sponge as an adjuvant therapy for rotator cuff repair - a phase 1, randomized, standard of care control, multicenter study: Safety and feasibility. *Am J Sports Med* **2015**, *43*, 1994-2004.

215. 32. Avanzi, P.; Giudici, L.D.; Capone, A.; Cardoni, G.; Lunardi, G.; Foti, G.; Zorzi, C., Prospective randomized controlled trial for patch augmentation in rotator cuff repair: 24-month outcomes. *J Shoulder Elbow Surg* **2019**, *28*, 1918-1927.

216. 33. Lamas, J.R.; García-Fernández, C.; Tornero-Esteban, P.; Lópiz, Y.; Rodriguez-Rodriguez, L.; Ortega, L.; Fernández-Gutiérrez, B.; Marco, F., Adverse effects of xenogenic scaffolding in the context of a randomized double-blind placebo-controlled study for repairing full-thickness rotator cuff tears. *Trials* **2019**, *20*, 387.

217. 34. Burks, R.T.; Crim, J.; Brown, N.; Fink, B.; Greis, P.E., A prospective randomized clinical trial comparing arthroscopic single- and double-row rotator cuff repair: Magnetic resonance imaging and early clinical evaluation. *Am J Sports Med* **2009**, *37*, 674-682.

218. 35. Carbonel, I.; Martinez, A.A.; Calvo, A.; Ripalda, J.; Herrera, A., Single-row versus double-row arthroscopic repair in the treatment of rotator cuff tears: A prospective randomized clinical study. *Int Orthop* **2012**, *36*, 1877-1883.

219. 36. Franceschi, F.; Papalia, R.; Franceschetti, E.; Palumbo, A.; Del Buono, A.; Paciotti, M.; Maffulli, N.; Denaro, V., Double-row repair lowers the retear risk after accelerated rehabilitation. *Am J Sports Med* **2016**, *44*, 948-956.

220. 37. Franceschi, F.; Ruzzini, L.; Longo, U.G.; Martina, F.M.; Zobel, B.B.; Maffulli, N.; Denaro, V., Equivalent clinical results of arthroscopic single-row and double-row suture anchor repair for rotator cuff tears: A randomized controlled trial. *Am J Sports Med* **2007**, *35*, 1254-1260.

221. 38. Gartsman, G.M.; Drake, G.; Edwards, T.B.; Elkousy, H.A.; Hammerman, S.M.; O'Connor, D.P.; Press, C.M., Ultrasound evaluation of arthroscopic full-thickness supraspinatus rotator cuff repair: Single-row versus double-row suture bridge (transosseous equivalent) fixation. Results of a prospective, randomized study. *J Shoulder Elbow Surg* **2013**, *22*, 1480-1487.
222. 39. Grasso, A.; Milano, G.; Salvatore, M.; Falcone, G.; Deriu, L.; Fabbriciani, C., Single-row versus double-row arthroscopic rotator cuff repair: A prospective randomized clinical study. *Arthroscopy* **2009**, *25*, 4-12.
223. 40. Kim, Y.S.; Lee, H.J.; Jin, H.K.; Kim, S.E.; Lee, J.W., Conventional en masse repair versus separate double-layer double-row repair for the treatment of delaminated rotator cuff tears. *Am J Sports Med* **2016**, *44*, 1146-1152.
224. 41. Koh, K.H.; Kang, K.C.; Lim, T.K.; Shon, M.S.; Yoo, J.C., Prospective randomized clinical trial of single- versus double-row suture anchor repair in 2- to 4-cm rotator cuff tears: Clinical and magnetic resonance imaging results. *Arthroscopy* **2011**, *27*, 453-462.
225. 42. Lapner, P.L.; Sabri, E.; Rakhra, K.; McRae, S.; Leiter, J.; Bell, K.; Macdonald, P., A multicenter randomized controlled trial comparing single-row with double-row fixation in arthroscopic rotator cuff repair. *J Bone Joint Surg Am* **2012**, *94*, 1249-1257.
226. 43. Ma, H.L.; Chiang, E.R.; Wu, H.T.; Hung, S.C.; Wang, S.T.; Liu, C.L.; Chen, T.H., Clinical outcome and imaging of arthroscopic single-row and double-row rotator cuff repair: A prospective randomized trial. *Arthroscopy* **2012**, *28*, 16-24.
227. 44. Nicholas, S.J.; Lee, S.J.; Mullaney, M.J.; Tyler, T.F.; Fukunaga, T.; Johnson, C.D.; McHugh, M.P., Functional outcomes after double-row versus single-row rotator cuff repair: A prospective randomized trial. *Orthop J Sports Med* **2016**, *4*, 2325967116667398.
228. 45. Iannotti, J.P.; Codsi, M.J.; Kwon, Y.W.; Derwin, K.; Ciccone, J.; Brems, J.J., Porcine small intestine submucosa augmentation of surgical repair of chronic two-tendon rotator cuff tears. A randomized, controlled trial. *The Journal of bone and joint surgery. American volume* **2006**, *88*, 1238-1244.
229. 46. Ide, J.; Mochizuki, Y.; van Noort, A.; Ochi, H.; Sridharan, S.; Itoi, E.; Greiner, S., Local rhbmp-12 on an absorbable collagen sponge as an adjuvant therapy for rotator cuff repair-a phase 1, randomized, standard of care control, multicenter study: Part 2-a pilot study of functional recovery and structural outcomes. *Orthop J Sports Med* **2017**, *5*, 2325967117726740.
230. 47. Alvarez, C.M.; Litchfield, R.; Jackowski, D.; Griffin, S.; Kirkley, A., A prospective, double-blind, randomized clinical trial comparing subacromial injection of betamethasone and xylocaine to xylocaine alone in chronic rotator cuff tendinosis. *Am J Sports Med* **2005**, *33*, 255-262.
231. 48. Bang, S.R.; Yu, S.K.; Kim, T.H., Can gabapentin help reduce postoperative pain in arthroscopic rotator cuff repair? A prospective, randomized, double-blind study. *Arthroscopy* **2010**, *26*, S106-111.
232. 49. Barber, F.A.; Herbert, M.A., The effectiveness of an anesthetic continuous-infusion device on postoperative pain control. *Arthroscopy* **2002**, *18*, 76-81.
233. 50. Behr, A.; Freo, U.; Ori, C.; Westermann, B.; Alemanno, F., Buprenorphine added to levobupivacaine enhances postoperative analgesia of middle interscalene brachial plexus block. *J Anesth* **2012**, *26*, 746-751.
234. 51. Borgeat, A.; Aguirre, J.; Marquardt, M.; Mrdjen, J.; Blumenthal, S., Continuous interscalene analgesia with ropivacaine 0.2% versus ropivacaine 0.3% after open rotator cuff repair: The effects on postoperative analgesia and motor function. *Anesth Analg* **2010**, *111*, 1543-1547.
235. 52. Cho, C.H.; Lee, S.W.; Lee, Y.K.; Shin, H.K.; Hwang, I., Effect of a sleep aid in analgesia after arthroscopic rotator cuff repair. *Yonsei Med J* **2015**, *56*, 772-777.

236. 53. Cho, C.H.; Song, K.S.; Min, B.W.; Lee, K.J.; Ha, E.; Lee, Y.C.; Lee, Y.K., Multimodal approach to postoperative pain control in patients undergoing rotator cuff repair. *Knee surgery, sports traumatology, arthroscopy : official journal of the ESSKA* **2011**, *19*, 1744-1748.

237. 54. Cho, N.S.; Ha, J.H.; Rhee, Y.G., Patient-controlled analgesia after arthroscopic rotator cuff repair: Subacromial catheter versus intravenous injection. *Am J Sports Med* **2007**, *35*, 75-79.

238. 55. Choi, S.; Kim, T.; Kwon, Y.S.; Kang, H., Intra-operative effect of interscalene brachial plexus block to arthroscopic rotator cuff repair surgery. *Int Orthop* **2018**.

239. 56. Chou, W.Y.; Ko, J.Y.; Wang, F.S.; Huang, C.C.; Wong, T.; Wang, C.J.; Chang, H.E., Effect of sodium hyaluronate treatment on rotator cuff lesions without complete tears: A randomized, double-blind, placebo-controlled study. *J Shoulder Elbow Surg* **2010**, *19*, 557-563.

240. 57. Coghlan, J.A.; Forbes, A.; McKenzie, D.; Bell, S.N.; Buchbinder, R., Efficacy of subacromial ropivacaine infusion for rotator cuff surgery. A randomized trial. *The Journal of bone and joint surgery. American volume* **2009**, *91*, 1558-1567.

241. 58. Culebras, X.; Van Gessel, E.; Hoffmeyer, P.; Gamulin, Z., Clonidine combined with a long acting local anesthetic does not prolong postoperative analgesia after brachial plexus block but does induce hemodynamic changes. *Anesth Analg* **2001**, *92*, 199-204.

242. 59. Delaunay, L.; Souron, V.; Lafosse, L.; Marret, E.; Toussaint, B., Analgesia after arthroscopic rotator cuff repair: Subacromial versus interscalene continuous infusion of ropivacaine. *Reg Anesth Pain Med* **2005**, *30*, 117-122.

243. 60. Desroches, A.; Klouche, S.; Schlur, C.; Bauer, T.; Waitzenegger, T.; Hardy, P., Suprascapular nerve block versus interscalene block as analgesia after arthroscopic rotator cuff repair: A randomized controlled noninferiority trial. *Arthroscopy* **2016**, *32*, 2203-2209.

244. 61. Gumina, S.; Passaretti, D.; Gurzì, M.D.; Candela, V., Arginine l-alpha-ketoglutarate, methylsulfonylmethane, hydrolyzed type i collagen and bromelain in rotator cuff tear repair: A prospective randomized study. *Curr Med Res Opin* **2012**, *28*, 1767-1774.

245. 62. Han, S.S.; Lee, Y.H.; Oh, J.H.; Aminzai, S.; Kim, S.H., Randomized, controlled trial of multimodal shoulder injection or intravenous patient-controlled analgesia after arthroscopic rotator cuff repair. *Knee surgery, sports traumatology, arthroscopy : official journal of the ESSKA* **2013**, *21*, 2877-2883.

246. 63. Hartrick, C.T.; Tang, Y.S.; Siwek, D.; Murray, R.; Hunstad, D.; Smith, G., The effect of initial local anesthetic dose with continuous interscalene analgesia on postoperative pain and diaphragmatic function in patients undergoing arthroscopic shoulder surgery: A double-blind, randomized controlled trial. *BMC Anesthesiol* **2012**, *12*, 6.

247. 64. Ikemoto, R.Y.; Murachovsky, J.; Prata Nascimento, L.G.; Bueno, R.S.; Oliveira Almeida, L.H.; Strose, E.; de Mello, S.C.; Saletti, D., Prospective randomized study comparing two anesthetic methods for shoulder surgery. *Revista brasileira de ortopedia* **2010**, *45*, 395-399.

248. 65. Jo, C.H.; Shin, J.S.; Huh, J., Multimodal analgesia for arthroscopic rotator cuff repair: A randomized, placebo-controlled, double-blind trial. *Eur J Orthop Surg Traumatol* **2014**, *24*, 315-322.

249. 66. Khashan, M.; Dolkart, O.; Amar, E.; Chechik, O.; Sharfman, Z.; Mozes, G.; Maman, E.; Weinbroum, A., Effect of preemptive intra-articular morphine and ketamine on pain after arthroscopic rotator cuff repair: A prospective, double-blind, randomized controlled study. *Arch Orthop Trauma Surg* **2016**, *136*, 233-239.

250. 67. Kim, J.H.; Koh, H.J.; Kim, D.K.; Lee, H.J.; Kwon, K.H.; Lee, K.Y.; Kim, Y.S., Interscalene brachial plexus bolus block versus patient-controlled interscalene indwelling catheter analgesia for the first 48 hours after arthroscopic rotator cuff repair. *J Shoulder Elbow Surg* **2018**, *27*, 1243-1250.

251. 68. Kim, J.Y.; Song, K.S.; Kim, W.J.; Park, Y.H.; Kang, H.; Woo, Y.C.; Shin, H.Y., Analgesic efficacy of two interscalene blocks and one cervical epidural block in arthroscopic rotator cuff repair. *Knee surgery, sports traumatology, arthroscopy : official journal of the ESSKA* **2016**, *24*, 931-939.

252. 69. Ko, S.H.; Cho, S.D.; Lee, C.C.; Choi, J.K.; Kim, H.W.; Park, S.J.; Bae, M.H.; Cha, J.R., Comparison of arthroscopically guided suprascapular nerve block and blinded axillary nerve block vs. Blinded suprascapular nerve block in arthroscopic rotator cuff repair: A randomized controlled trial. *Clin Orthop Surg* **2017**, *9*, 340-347.

253. 70. Koh, W.U.; Kim, H.J.; Park, H.S.; Choi, W.J.; Yang, H.S.; Ro, Y.J., A randomised controlled trial comparing continuous supraclavicular and interscalene brachial plexus blockade for open rotator cuff surgery. *Anaesthesia* **2016**, *71*, 692-699.

254. 71. Lee, J.J.; Hwang, J.T.; Kim, D.Y.; Lee, S.S.; Hwang, S.M.; Lee, N.R.; Kwak, B.C., Effects of arthroscopy-guided suprascapular nerve block combined with ultrasound-guided interscalene brachial plexus block for arthroscopic rotator cuff repair: A randomized controlled trial. *Knee surgery, sports traumatology, arthroscopy : official journal of the ESSKA* **2017**, *25*, 2121-2128.

255. 72. Lee, J.J.; Kim, D.Y.; Hwang, J.T.; Lee, S.S.; Hwang, S.M.; Kim, G.H.; Jo, Y.G., Effect of ultrasonographically guided axillary nerve block combined with suprascapular nerve block in arthroscopic rotator cuff repair: A randomized controlled trial. *Arthroscopy* **2014**, *30*, 906-914.

256. 73. Lee, J.J.; Yoo, Y.S.; Hwang, J.T.; Kim, D.Y.; Jeon, S.J.; Hwang, S.M.; Jang, J.S., Efficacy of direct arthroscopy-guided suprascapular nerve block after arthroscopic rotator cuff repair: A prospective randomized study. *Knee surgery, sports traumatology, arthroscopy : official journal of the ESSKA* **2015**, *23*, 562-566.

257. 74. Liu, X.N.; Noh, Y.M.; Yang, C.J.; Kim, J.U.; Chung, M.H.; Noh, K.C., Effects of a single-dose interscalene block on pain and stress biomarkers in patients undergoing arthroscopic rotator cuff repair: A randomized controlled trial. *Arthroscopy* **2017**, *33*, 918-926.

258. 75. Malik, T.; Mass, D.; Cohn, S., Postoperative analgesia in a prolonged continuous interscalene block versus single-shot block in outpatient arthroscopic rotator cuff repair: A prospective randomized study. *Arthroscopy* **2016**, *32*, 1544-1550.e1541.

259. 76. Merivirta, R.; Äärimaa, V.; Aantaa, R.; Koivisto, M.; Leino, K.; Liukas, A.; Kuusniemi, K., Postoperative fentanyl patch versus subacromial bupivacaine infusion in arthroscopic shoulder surgery. *Arthroscopy* **2013**, *29*, 1129-1134.

260. 77. Oh, C.H.; Oh, J.H.; Kim, S.H.; Cho, J.H.; Yoon, J.P.; Kim, J.Y., Effectiveness of subacromial anti-adhesive agent injection after arthroscopic rotator cuff repair: Prospective randomized comparison study. *Clin Orthop Surg* **2011**, *3*, 55-61.

261. 78. Osti, L.; Buono, A.D.; Maffulli, N., Pulsed electromagnetic fields after rotator cuff repair: A randomized, controlled study. *Orthopedics* **2015**, *38*, e223-228.

262. 79. Park, J.Y.; Bang, J.Y.; Oh, K.S., Blind suprascapular and axillary nerve block for post-operative pain in arthroscopic rotator cuff surgery. *Knee surgery, sports traumatology, arthroscopy : official journal of the ESSKA* **2016**, *24*, 3877-3883.

263. 80. Salviz, E.A.; Xu, D.; Frulla, A.; Kwofie, K.; Shastri, U.; Chen, J.; Shariat, A.N.; Littwin, S.; Lin, E.; Choi, J.*, et al.*, Continuous interscalene block in patients having outpatient rotator cuff repair surgery: A prospective randomized trial. *Anesth Analg* **2013**, *117*, 1485-1492.

264. 81. Seven, M.M.; Ersen, O.; Akpancar, S.; Ozkan, H.; Turkkan, S.; Yıldız, Y.; Koca, K., Effectiveness of prolotherapy in the treatment of chronic rotator cuff lesions. *Orthop Traumatol Surg Res* **2017**, *103*, 427-433.

265. 82. Takada, M.; Fukusaki, M.; Terao, Y.; Yamashita, K.; Ando, Y.; Sumikawa, K., Postoperative analgesic effect of preoperative intravenous flurbiprofen in arthroscopic rotator cuff repair. *J Anesth* **2009**, *23*, 500-503.

266. 83. Tetzlaff, J.E.; Brems, J.; Dilger, J., Intraarticular morphine and bupivacaine reduces postoperative pain after rotator cuff repair. *Reg Anesth Pain Med* **2000**, *25*, 611-614.

267. 84. Thackeray, E.M.; Swenson, J.D.; Gertsch, M.C.; Phillips, K.M.; Steele, J.W.; Burks, R.T.; Tashjian, R.Z.; Greis, P.E., Diaphragm function after interscalene brachial plexus block: A double-blind, randomized comparison of 0.25% and 0.125% bupivacaine. *J Shoulder Elbow Surg* **2013**, *22*, 381-386.

268. 85. Watanabe, K.; Tokumine, J.; Yorozu, T.; Moriyama, K.; Sakamoto, H.; Inoue, T., Particulate-steroid betamethasone added to ropivacaine in interscalene brachial plexus block for arthroscopic rotator cuff repair improves postoperative analgesia. *BMC Anesthesiol* **2016**, *16*, 84.

269. 86. Yamakado, K., Efficacy of arthroscopically placed pain catheter adjacent to the suprascapular nerve (continuous arthroscopically assisted suprascapular nerve block) following arthroscopic rotator-cuff repair. *Open Access J Sports Med* **2014**, *5*, 129-136.

270. 87. Yamamoto, S.; Yamaguchi, H.; Sakaguchi, M.; Yamashita, S.; Satsumae, T., Preoperative droperidol improved postoperative pain relief in patients undergoing rotator-cuff repair during general anesthesia using intravenous morphine. *J Clin Anesth* **2003**, *15*, 525-529.

271. 88. Yun, M.J.; Oh, J.H.; Yoon, J.P.; Park, S.H.; Hwang, J.W.; Kil, H.Y., Subacromial patient-controlled analgesia with ropivacaine provides effective pain control after arthroscopic rotator cuff repair. *Knee surgery, sports traumatology, arthroscopy : official journal of the ESSKA* **2012**, *20*, 1971-1977.

272. 89. Menek, B.; Tarakci, D.; Algun, Z.C., The effect of mulligan mobilization on pain and life quality of patients with rotator cuff syndrome: A randomized controlled trial. *J Back Musculoskelet Rehabil* **2019**, *32*, 171-178.

273. 90. Coory, J.A.; Parr, A.F.; Wilkinson, M.P.; Gupta, A., Efficacy of suprascapular nerve block compared with subacromial injection: A randomized controlled trial in patients with rotator cuff tears. *J Shoulder Elbow Surg* **2019**, *28*, 430-436.

274. 91. Akbaba, Y.A.; Mutlu, E.K.; Altun, S.; Turkmen, E.; Birinci, T.; Celik, D., The effectiveness of trigger point treatment in rotator cuff pathology: A randomized controlled double-blind study. *J Back Musculoskelet Rehabil* **2019**, *32*, 519-527.

275. 92. Banerjee, S.S.; Pulido, P.; Adelson, W.S.; Fronek, J.; Hoenecke, H.R., The efficacy of continuous bupivacaine infiltration following arthroscopic rotator cuff repair. *Arthroscopy* **2008**, *24*, 397-402.

276. 93. Gialanella, B.; Prometti, P., Effects of corticosteroids injection in rotator cuff tears. *Pain Med* **2011**, *12*, 1559-1565.

277. 94. Lee, H.J.; Kim, Y.S.; Park, I.; Ha, D.H.; Lee, J.H., Administration of analgesics after rotator cuff repair: A prospective clinical trial comparing glenohumeral, subacromial, and a combination of glenohumeral and subacromial injections. *J Shoulder Elbow Surg* **2015**, *24*, 663-668.

278. 95. Perdreau, A.; Joudet, T., Efficacy of multimodal analgesia injection combined with corticosteroids after arthroscopic rotator cuff repair. *Orthop Traumatol Surg Res* **2015**, *101*, S337-345.

279. 96. Ganokroj, P.; Matrakool, L.; Limsuwarn, P.; Sissaynarane, T.; Yimvassana, C.; Laoratanavoraphong, S.; Ratanawarinchai, J., A prospective randomized study comparing the effectiveness of midlateral and posterior subacromial steroid injections. *Orthopedics* **2019**, *42*, e44-e50.

280. 97. Schwartzberg, R.S.; Reuss, B.L.; Rust, R., Efficacy of continuous subacromial bupivacaine infusion for pain control after arthroscopic rotator cuff repair. *J Shoulder Elbow Surg* **2013**, *22*, 1320-1324.

281. 98. Desmet, M.; Vanneste, B.; Reynvoet, M.; Van Cauwelaert, J.; Verhelst, L.; Pottel, H.; Missant, C.; Van de Velde, M., A randomised controlled trial of intravenous dexamethasone combined with interscalene brachial plexus blockade for shoulder surgery. *Anaesthesia* **2015**, *70*, 1180-1185.

282. 99. Kim, J.Y.; Song, S.H.; Cho, J.H.; Cho, H.R., Comparison of clinical efficacy among remifentanil, nicardipine, and remifentanil plus nicardipine continuous infusion for hypotensive anesthesia during arthroscopic shoulder surgery. *J Orthop Surg (Hong Kong)* **2017**, *25*, 2309499017716251.

283. 100. Syed, U.A.M.; Aleem, A.W.; Wowkanech, C.; Weekes, D.; Freedman, M.; Tjoumakaris, F.; Abboud, J.A.; Austin, L.S., Neer award 2018: The effect of preoperative education on opioid consumption in patients undergoing arthroscopic rotator cuff repair: A prospective, randomized clinical trial. *J Shoulder Elbow Surg* **2018**, *27*, 962-967.

284. 101. Kim, Y.S.; Lee, H.J.; Kim, J.H.; Noh, D.Y., When should we repair partial-thickness rotator cuff tears? Outcome comparison between immediate surgical repair versus delayed repair after 6-month period of nonsurgical treatment. *Am J Sports Med* **2018**, *46*, 1091-1096.

285. 102. Moosmayer, S.; Lund, G.; Seljom, U.S.; Haldorsen, B.; Svege, I.C.; Hennig, T.; Pripp, A.H.; Smith, H.J., At a 10-year follow-up, tendon repair is superior to physiotherapy in the treatment of small and medium-sized rotator cuff tears. *The Journal of bone and joint surgery. American volume* **2019**, *101*, 1050-1060.

286. 103. Lambers Heerspink, F.O.; van Raay, J.J.; Koorevaar, R.C.; van Eerden, P.J.; Westerbeek, R.E.; van 't Riet, E.; van den Akker-Scheek, I.; Diercks, R.L., Comparing surgical repair with conservative treatment for degenerative rotator cuff tears: A randomized controlled trial. *J Shoulder Elbow Surg* **2015**, *24*, 1274-1281.

287. 104. Kukkonen, J.; Joukainen, A.; Lehtinen, J.; Mattila, K.T.; Tuominen, E.K.; Kauko, T.; Äärimaa, V., Treatment of non-traumatic rotator cuff tears: A randomised controlled trial with one-year clinical results. *Bone Joint J* **2014**, *96-B*, 75-81.

288. 105. Moosmayer, S.; Lund, G.; Seljom, U.; Svege, I.; Hennig, T.; Tariq, R.; Smith, H.J., Comparison between surgery and physiotherapy in the treatment of small and medium-sized tears of the rotator cuff: A randomised controlled study of 103 patients with one-year follow-up. *J Bone Joint Surg Br* **2010**, *92*, 83-91.

289. 106. Aagaard, K.E.; Lunsjö, K.; Frobell, R., Early repair of trauma-related full-thickness rotator cuff tears does not eliminate the problem of healing failure. *Bone Joint J* **2019**, *101-B*, 603-609.

290. 107. Analay Akbaba, Y.; Kaya Mutlu, E.; Altun, S.; Celik, D., Does the patients' expectations on kinesiotape affect the outcomes of patients with a rotator cuff tear? A randomized controlled clinical trial. *Clin Rehabil* **2018**, *32*, 1509-1519.

291. 108. Analan, P.D.; Leblebici, B.; Adam, M., Effects of therapeutic ultrasound and exercise on pain, function, and isokinetic shoulder rotator strength of patients with rotator cuff disease. *J Phys Ther Sci* **2015**, *27*, 3113-3117.

292. 109. Arias-Buría, J.L.; Valero-Alcaide, R.; Cleland, J.A.; Salom-Moreno, J.; Ortega-Santiago, R.; Atín-Arratibel, M.A.; Fernández-de-las-Peñas, C., Inclusion of trigger point dry needling in a multimodal physical therapy program for postoperative shoulder pain: A randomized clinical trial. *J Manipulative Physiol Ther* **2015**, *38*, 179-187.

293. 110. Arndt, J.; Clavert, P.; Mielcarek, P.; Bouchaib, J.; Meyer, N.; Kempf, J.F.; (SOFEC), F.S.f.S.E., Immediate passive motion versus immobilization after endoscopic supraspinatus tendon repair: A prospective randomized study. *Orthop Traumatol Surg Res* **2012**, *98*, S131-138.

294. 111. Baumgarten, K.M.; Osborn, R.; Schweinle, W.E.; Zens, M.J.; Helsper, E.A., Are pulley exercises initiated 6 weeks after rotator cuff repair a safe and effective rehabilitative treatment? A randomized controlled trial. *Am J Sports Med* **2016**, *44*, 1844-1851.

295. 112. Blum, K.; Chen, A.L.; Chen, T.J.; Waite, R.L.; Downs, B.W.; Braverman, E.R.; Kerner, M.M.; Savarimuthu, S.M.; DiNubile, N., Repetitive h-wave device stimulation and program induces significant increases in the range of motion of post operative rotator cuff reconstruction in a double-blinded randomized placebo controlled human study. *BMC Musculoskelet Disord* **2009**, *10*, 132.

296. 113. Cuff, D.J.; Pupello, D.R., Prospective randomized study of arthroscopic rotator cuff repair using an early versus delayed postoperative physical therapy protocol. *J Shoulder Elbow Surg* **2012**, *21*, 1450-1455.

297. 114. De Roo, P.J.; Muermans, S.; Maroy, M.; Linden, P.; Van den Daelen, L., Passive mobilization after arthroscopic rotator cuff repair is not detrimental in the early postoperative period. *Acta Orthop Belg* **2015**, *81*, 485-492.

298. 115. Düzgün, I.; Baltacı, G.; Atay, O.A., Comparison of slow and accelerated rehabilitation protocol after arthroscopic rotator cuff repair: Pain and functional activity. *Acta Orthop Traumatol Turc* **2011**, *45*, 23-33.

299. 116. Düzgün, İ.; Baltacı, G.; Turgut, E.; Atay, O.A., Effects of slow and accelerated rehabilitation protocols on range of motion after arthroscopic rotator cuff repair. *Acta Orthop Traumatol Turc* **2014**, *48*, 642-648.

300. 117. Hayes, K.; Ginn, K.A.; Walton, J.R.; Szomor, Z.L.; Murrell, G.A., A randomised clinical trial evaluating the efficacy of physiotherapy after rotator cuff repair. *Aust J Physiother* **2004**, *50*, 77-83.

301. 118. Jenssen, K.K.; Lundgreen, K.; Madsen, J.E.; Kvakestad, R.; Pripp, A.H.; Dimmen, S., No functional difference between three and six weeks of immobilization after arthroscopic rotator cuff repair: A prospective randomized controlled non-inferiority trial. *Arthroscopy* **2018**, *34*, 2765-2774.

302. 119. Keener, J.D.; Galatz, L.M.; Stobbs-Cucchi, G.; Patton, R.; Yamaguchi, K., Rehabilitation following arthroscopic rotator cuff repair: A prospective randomized trial of immobilization compared with early motion. *J Bone Joint Surg Am* **2014**, *96*, 11-19.

303. 120. Kim, J.Y.; Lee, J.S.; Park, C.W., Extracorporeal shock wave therapy is not useful after arthroscopic rotator cuff repair. *Knee Surg Sports Traumatol Arthrosc* **2012**, *20*, 2567-2572.

304. 121. Kim, Y.S.; Chung, S.W.; Kim, J.Y.; Ok, J.H.; Park, I.; Oh, J.H., Is early passive motion exercise necessary after arthroscopic rotator cuff repair? *Am J Sports Med* **2012**, *40*, 815-821.

305. 122. Koh, K.H.; Lim, T.K.; Shon, M.S.; Park, Y.E.; Lee, S.W.; Yoo, J.C., Effect of immobilization without passive exercise after rotator cuff repair: Randomized clinical trial comparing four and eight weeks of immobilization. *The Journal of bone and joint surgery. American volume* **2014**, *96*, e44.

306. 123. Kraeutler, M.J.; Reynolds, K.A.; Long, C.; McCarty, E.C., Compressive cryotherapy versus ice-a prospective, randomized study on postoperative pain in patients undergoing arthroscopic rotator cuff repair or subacromial decompression. *J Shoulder Elbow Surg* **2015**, *24*, 854-859.

307. 124. Lam, P.H.; Hansen, K.; Keighley, G.; Hackett, L.; Murrell, G.A., A randomized, double-blinded, placebo-controlled clinical trial evaluating the effectiveness of daily vibration after arthroscopic rotator cuff repair. *Am J Sports Med* **2015**, *43*, 2774-2782.

308. 125. Lastayo, P.C.; Wright, T.; Jaffe, R.; Hartzel, J., Continuous passive motion after repair of the rotator cuff. A prospective outcome study. *The Journal of bone and joint surgery. American volume* **1998**, *80*, 1002-1011.

309. 126. Lee, B.G.; Cho, N.S.; Rhee, Y.G., Effect of two rehabilitation protocols on range of motion and healing rates after arthroscopic rotator cuff repair: Aggressive versus limited early passive exercises. *Arthroscopy* **2012**, *28*, 34-42.

310. 127. Mahure, S.A.; Rokito, A.S.; Kwon, Y.W., Transcutaneous electrical nerve stimulation for postoperative pain relief after arthroscopic rotator cuff repair: A prospective double-blinded randomized trial. *J Shoulder Elbow Surg* **2017**, *26*, 1508-1513.

311. 128. Mazzocca, A.D.; Arciero, R.A.; Shea, K.P.; Apostolakos, J.M.; Solovyova, O.; Gomlinski, G.; Wojcik, K.E.; Tafuto, V.; Stock, H.; Cote, M.P., The effect of early range of motion on quality of life, clinical outcome, and repair integrity after arthroscopic rotator cuff repair. *Arthroscopy* **2017**, *33*, 1138-1148.

312. 129. Osbahr, D.C.; Cawley, P.W.; Speer, K.P., The effect of continuous cryotherapy on glenohumeral joint and subacromial space temperatures in the postoperative shoulder. *Arthroscopy* **2002**, *18*, 748-754.

313. 130. Piitulainen, K.; Häkkinen, A.; Salo, P.; Kautiainen, H.; Ylinen, J., Does adding a 12-month exercise programme to usual care after a rotator cuff repair effect disability and quality of life at 12 months? A randomized controlled trial. *Clin Rehabil* **2015**, *29*, 447-456.

314. 131. Raab, M.G.; Rzeszutko, D.; O'Connor, W.; Greatting, M.D., Early results of continuous passive motion after rotator cuff repair: A prospective, randomized, blinded, controlled study. *Am J Orthop (Belle Mead NJ)* **1996**, *25*, 214-220.

315. 132. Roddey, T.S.; Olson, S.L.; Gartsman, G.M.; Hanten, W.P.; Cook, K.F., A randomized controlled trial comparing 2 instructional approaches to home exercise instruction following arthroscopic full-thickness rotator cuff repair surgery. *J Orthop Sports Phys Ther* **2002**, *32*, 548-559.

316. 133. Sheps, D.M.; Silveira, A.; Beaupre, L.; Styles-Tripp, F.; Balyk, R.; Lalani, A.; Glasgow, R.; Bergman, J.; Bouliane, M.; (SURGE), S.a.U.E.R.G.o.E., Early active motion versus sling immobilization after arthroscopic rotator cuff repair: A randomized controlled trial. *Arthroscopy* **2019**, *35*, 749-760.e742.

317. 134. Sheps, D.M.; Bouliane, M.; Styles-Tripp, F.; Beaupre, L.A.; Saraswat, M.K.; Luciak-Corea, C.; Silveira, A.; Glasgow, R.; Balyk, R., Early mobilisation following mini-open rotator cuff repair: A randomised control trial. *Bone Joint J* **2015**, *97-B*, 1257-1263.

318. 135. Garofalo, R.; Conti, M.; Notarnicola, A.; Maradei, L.; Giardella, A.; Castagna, A., Effects of one-month continuous passive motion after arthroscopic rotator cuff repair: Results at 1-year follow-up of a prospective randomized study. *Musculoskelet Surg* **2010**, *94 Suppl 1*, S79-83.

319. 136. Klintberg, I.H.; Gunnarsson, A.C.; Svantesson, U.; Styf, J.; Karlsson, J., Early loading in physiotherapy treatment after full-thickness rotator cuff repair: A prospective randomized pilot-study with a two-year follow-up. *Clin Rehabil* **2009**, *23*, 622-638.

320. 137. Raschhofer, R.; Poulios, N.; Schimetta, W.; Kisling, R.; Mittermaier, C., Early active rehabilitation after arthroscopic rotator cuff repair: A prospective randomized pilot study. *Clin Rehabil* **2017**, *31*, 1332-1339.

321. 138. De Carli, A.; Vadalà, A.; Zanzotto, E.; Zampar, G.; Vetrano, M.; Iorio, R.; Ferretti, A., Reparable rotator cuff tears with concomitant long-head biceps lesions: Tenotomy or tenotomy/tenodesis? *Knee surgery, sports traumatology, arthroscopy : official journal of the ESSKA* **2012**, *20*, 2553-2558.

322. 139. Lee, H.J.; Jeong, J.Y.; Kim, C.K.; Kim, Y.S., Surgical treatment of lesions of the long head of the biceps brachii tendon with rotator cuff tear: A prospective randomized clinical trial comparing the clinical results of tenotomy and tenodesis. *J Shoulder Elbow Surg* **2016**, *25*, 1107-1114.

323. 140. Oh, J.H.; Lee, Y.H.; Kim, S.H.; Park, J.S.; Seo, H.J.; Kim, W.; Park, H.B., Comparison of treatments for superior labrum-biceps complex lesions with concomitant rotator cuff repair: A prospective, randomized, comparative analysis of debridement, biceps tenotomy, and biceps tenodesis. *Arthroscopy* **2016**, *32*, 958-967.

324. 141. Zhang, Q.; Zhou, J.; Ge, H.; Cheng, B., Tenotomy or tenodesis for long head biceps lesions in shoulders with reparable rotator cuff tears: A prospective randomised trial. *Knee surgery, sports traumatology, arthroscopy : official journal of the ESSKA* **2015**, *23*, 464-469.

325. 142. Belay, E.S.; Wittstein, J.R.; Garrigues, G.E.; Lassiter, T.E.; Scribani, M.; Goldner, R.D.; Bean, C.A., Biceps tenotomy has earlier pain relief compared to biceps tenodesis: A randomized prospective study. *Knee surgery, sports traumatology, arthroscopy : official journal of the ESSKA* **2019**, *27*, 4032-4037.

326. 143. Hufeland, M.; Wicke, S.; Verde, P.E.; Krauspe, R.; Patzer, T., Biceps tenodesis versus tenotomy in isolated lhb lesions: A prospective randomized clinical trial. *Arch Orthop Trauma Surg* **2019**, *139*, 961-970.

327. 144. Carr, A.; Cooper, C.; Campbell, M.K.; Rees, J.; Moser, J.; Beard, D.J.; Fitzpatrick, R.; Gray, A.; Dawson, J.; Murphy, J.*, et al.*, Effectiveness of open and arthroscopic rotator cuff repair (ukuff): A randomised controlled trial. *Bone Joint J* **2017**, *99-B*, 107-115.

328. 145. Cho, C.H.; Song, K.S.; Jung, G.H.; Lee, Y.K.; Shin, H.K., Early postoperative outcomes between arthroscopic and mini-open repair for rotator cuff tears. *Orthopedics* **2012**, *35*, e1347-1352.

329. 146. Liu, J.; Fan, L.; Zhu, Y.; Yu, H.; Xu, T.; Li, G., Comparison of clinical outcomes in all-arthroscopic versus mini-open repair of rotator cuff tears: A randomized clinical trial. *Medicine (Baltimore)* **2017**, *96*, e6322.

330. 147. van der Zwaal, P.; Thomassen, B.J.; Nieuwenhuijse, M.J.; Lindenburg, R.; Swen, J.W.; van Arkel, E.R., Clinical outcome in all-arthroscopic versus mini-open rotator cuff repair in small to medium-sized tears: A randomized controlled trial in 100 patients with 1-year follow-up. *Arthroscopy* **2013**, *29*, 266-273.

331. 148. Flurin, P.H.; Hardy, P.; Abadie, P.; Desmoineaux, P.; Essig, J.; Joudet, T.; Sommaire, C.; Thelu, C.E.; (SFA), F.A.S., Rotator cuff tears after 70 years of age: A prospective, randomized, comparative study between decompression and arthroscopic repair in 154 patients. *Orthop Traumatol Surg Res* **2013**, *99*, S371-378.

332. 149. Gartsman, G.M.; O'connor, D.P., Arthroscopic rotator cuff repair with and without arthroscopic subacromial decompression: A prospective, randomized study of one-year outcomes. *J Shoulder Elbow Surg* **2004**, *13*, 424-426.

333. 150. Milano, G.; Grasso, A.; Salvatore, M.; Zarelli, D.; Deriu, L.; Fabbriciani, C., Arthroscopic rotator cuff repair with and without subacromial decompression: A prospective randomized study. *Arthroscopy* **2007**, *23*, 81-88.

334. 151. Nam, J.H.; Park, S.; Lee, H.R.; Kim, S.H., Outcomes after limited or extensive bursectomy during rotator cuff repair: Randomized controlled trial. *Arthroscopy* **2018**.

335. 152. Boehm, T.D.; Werner, A.; Radtke, S.; Mueller, T.; Kirschner, S.; Gohlke, F., The effect of suture materials and techniques on the outcome of repair of the rotator cuff: A prospective, randomised study. *J Bone Joint Surg Br* **2005**, *87*, 819-823.

336. 153. Randelli, P.; Arrigoni, P.; Aliprandi, A.; Sdao, S.; Ragone, V.; D'Ambrosi, R.; Randelli, F.; Cabitza, P.; Banfi, G., Repair versus shaving of partial-thickness articular-sided tears of the upper subscapularis tendon. A prospective randomized controlled trial. *Joints* **2015**, *3*, 109-115.

337. 154. Bigoni, M.; Gorla, M.; Guerrasio, S.; Brignoli, A.; Cossio, A.; Grillo, P.; Marinoni, E.C., Shoulder evaluation with isokinetic strength testing after arthroscopic rotator cuff repairs. *J Shoulder Elbow Surg* **2009**, *18*, 178-183.

338. 155. Franceschi, F.; Papalia, R.; Del Buono, A.; Vasta, S.; Costa, V.; Maffulli, N.; Denaro, V., Articular-sided rotator cuff tears: Which is the best repair? A three-year prospective randomised controlled trial. *Int Orthop* **2013**, *37*, 1487-1493.

339. 156. Ko, S.H.; Lee, C.C.; Friedman, D.; Park, K.B.; Warner, J.J., Arthroscopic single-row supraspinatus tendon repair with a modified mattress locking stitch: A prospective, randomized controlled comparison with a simple stitch. *Arthroscopy* **2008**, *24*, 1005-1012.

340. 157. Milano, G.; Grasso, A.; Salvatore, M.; Saccomanno, M.F.; Deriu, L.; Fabbriciani, C., Arthroscopic rotator cuff repair with metal and biodegradable suture anchors: A prospective randomized study. *Arthroscopy* **2010**, *26*, S112-119.
341. 158. Shin, S.J., A comparison of 2 repair techniques for partial-thickness articular-sided rotator cuff tears. *Arthroscopy* **2012**, *28*, 25-33.
342. 159. Mardani-Kivi, M.; Keyhani, S.; Ebrahim-Zadeh, M.H.; Hashemi-Motlagh, K.; Saheb-Ekhtiari, K., Rotator cuff tear with concomitant long head of biceps tendon (lhbt) degeneration: What is the preferred choice? Open subpectoral versus arthroscopic intraarticular tenodesis. *J Orthop Traumatol* **2019**, *20*, 26.
343. 160. Randelli, P.; Stoppani, C.A.; Zaolino, C.; Menon, A.; Randelli, F.; Cabitza, P., Advantages of arthroscopic rotator cuff repair with a transosseous suture technique: A prospective randomized controlled trial. *Am J Sports Med* **2017**, *45*, 2000-2009.
344. 161. Kim, J.H.; Ha, D.H.; Kim, S.M.; Kim, K.W.; Han, S.Y.; Kim, Y.S., Does arthroscopic preemptive extensive rotator interval release reduce postoperative stiffness after arthroscopic rotator cuff repair?: A prospective randomized clinical trial. *J Shoulder Elbow Surg* **2019**, *28*, 1639-1646.
345. 162. Berth, A.; Neumann, W.; Awiszus, F.; Pap, G., Massive rotator cuff tears: Functional outcome after debridement or arthroscopic partial repair. *J Orthop Traumatol* **2010**, *11*, 13-20.
346. 163. Blanchard, T.K.; Bearcroft, P.W.; Constant, C.R.; Griffin, D.R.; Dixon, A.K., Diagnostic and therapeutic impact of mri and arthrography in the investigation of full-thickness rotator cuff tears. *Eur Radiol* **1999**, *9*, 638-642.
347. 164. Capito, N.M.; Cook, J.L.; Yahuaca, B.; Capito, M.D.; Sherman, S.L.; Smith, M.J., Safety and efficacy of hyperosmolar irrigation solution in shoulder arthroscopy. *J Shoulder Elbow Surg* **2017**, *26*, 745-751.
348. 165. Castagna, A.; Borroni, M.; Garofalo, R.; Rose, G.D.; Cesari, E.; Padua, R.; Conti, M.; Gumina, S., Deep partial rotator cuff tear: Transtendon repair or tear completion and repair? A randomized clinical trial. *Knee surgery, sports traumatology, arthroscopy : official journal of the ESSKA* **2015**, *23*, 460-463.
349. 166. Chierichini, A.; Frassanito, L.; Vergari, A.; Santoprete, S.; Chiarotti, F.; Saccomanno, M.F.; Milano, G., The effect of norepinephrine versus epinephrine in irrigation fluid on the incidence of hypotensive/bradycardic events during arthroscopic rotator cuff repair with interscalene block in the sitting position. *Arthroscopy* **2015**, *31*, 800-806.
350. 167. Franceschi, F.; Longo, U.G.; Ruzzini, L.; Rizzello, G.; Maffulli, N.; Denaro, V., No advantages in repairing a type ii superior labrum anterior and posterior (slap) lesion when associated with rotator cuff repair in patients over age 50: A randomized controlled trial. *Am J Sports Med* **2008**, *36*, 247-253.
351. 168. Gervasi, E.; Maman, E.; Dekel, A.; Cautero, E., Fluoroscopy-guided biodegradable spacer implantation using local anesthesia: Safety and efficacy study in patients with massive rotator cuff tears. *Musculoskelet Surg* **2016**, *100*, 19-24.
352. 169. Kim, J.; Chung, J.; Ok, H., Asymptomatic acromioclavicular joint arthritis in arthroscopic rotator cuff tendon repair: A prospective randomized comparison study. *Arch Orthop Trauma Surg* **2011**, *131*, 363-369.
353. 170. Milano, G.; Saccomanno, M.F.; Careri, S.; Taccardo, G.; De Vitis, R.; Fabbriciani, C., Efficacy of marrow-stimulating technique in arthroscopic rotator cuff repair: A prospective randomized study. *Arthroscopy* **2013**, *29*, 802-810.
354. 171. Oh, J.H.; Kim, J.Y.; Choi, J.H.; Park, S.M., Is arthroscopic distal clavicle resection necessary for patients with radiological acromioclavicular joint arthritis and rotator cuff tears? A prospective randomized comparative study. *Am J Sports Med* **2014**, *42*, 2567-2573.

355. 172. Oh, J.H.; Kim, J.Y.; Chung, S.W.; Park, J.S.; Kim, D.H.; Kim, S.H.; Yun, M.J., Warmed irrigation fluid does not decrease perioperative hypothermia during arthroscopic shoulder surgery. *Arthroscopy* **2014**, *30*, 159-164.

356. 173. Osti, L.; Del Buono, A.; Maffulli, N., Microfractures at the rotator cuff footprint: A randomised controlled study. *Int Orthop* **2013**, *37*, 2165-2171.

357. 174. Park, Y.B.; Koh, K.H.; Shon, M.S.; Park, Y.E.; Yoo, J.C., Arthroscopic distal clavicle resection in symptomatic acromioclavicular joint arthritis combined with rotator cuff tear: A prospective randomized trial. *Am J Sports Med* **2015**, *43*, 985-990.

358. 175. Senekovic, V.; Poberaj, B.; Kovacic, L.; Mikek, M.; Adar, E.; Markovitz, E.; Maman, E.; Dekel, A., The biodegradable spacer as a novel treatment modality for massive rotator cuff tears: A prospective study with 5-year follow-up. *Arch Orthop Trauma Surg* **2017**, *137*, 95-103.

359. 176. Torrens, C.; Miquel, J.; Santana, F., Do we really allow patient decision-making in rotator cuff surgery? A prospective randomized study. *J Orthop Surg Res* **2019**, *14*, 116.

360. 177. Nicholson, J.A.; Searle, H.K.C.; MacDonald, D.; McBirnie, J., Cost-effectiveness and satisfaction following arthroscopic rotator cuff repair: Does age matter? *Bone Joint J* **2019**, *101-B*, 860-866.

361. 178. Shibata, Y.; Midorikawa, K.; Emoto, G.; Naito, M., Clinical evaluation of sodium hyaluronate for the treatment of patients with rotator cuff tear. *J Shoulder Elbow Surg* **2001**, *10*, 209-216.

362. 179. Conti, M.; Garofalo, R.; Castagna, A., Does a brace influence clinical outcomes after arthroscopic rotator cuff repair? *Musculoskelet Surg* **2015**, *99 Suppl 1*, S31-35.

363. 180. Hollman, F.; Wolterbeek, N.; Zijl, J.A.C.; van Egeraat, S.P.M.; Wessel, R.N., Abduction brace versus antirotation sling after arthroscopic cuff repair: The effects on pain and function. *Arthroscopy* **2017**, *33*, 1618-1626.

364. 181. Ghandour, T.M.; Ibrahim, A.; Abdelrahman, A.A.; Elgammal, A.; Hammad, M.H., Does the type of shoulder brace affect postoperative pain and clinical outcome after arthroscopic rotator cuff repair? *Arthroscopy* **2019**, *35*, 1016-1023.

365. 182. Tirefort, J.; Schwitzguebel, A.J.; Collin, P.; Nowak, A.; Plomb-Holmes, C.; Ladermann, A., Postoperative mobilization after superior rotator cuff repair: Sling versus no sling: A randomized prospective study. *The Journal of bone and joint surgery. American volume* **2019**, *101*, 494-503.

366.
